# Structure-Guided Design of Proteomimetics Targeting the SARS-CoV‑2 S‑RBD/hACE2 Interface

**DOI:** 10.1021/acs.jmedchem.5c03450

**Published:** 2026-06-15

**Authors:** Sára Ferková, Agathe Fayolle, Olivier Boisvert, Ulrike Froehlich, Marie-Édith Nepveu-Traversy, Pierre Lavigne, Michel Grandbois, Philippe Sarret, Pierre-Luc Boudreault

**Affiliations:** † Department of Pharmacology and Physiology, Faculty of Medicine and Health Sciences, Institut de Pharmacologie de Sherbrooke, 7321 Université de Sherbrooke, 3001 12e Avenue Nord, Sherbrooke, Quebec J1H 5N4, Canada; ‡ Department of Biochemistry and Functional Genomics, Faculty of Medicine and Health Sciences, Université de Sherbrooke, 3001 12e Avenue Nord, Sherbrooke, Quebec J1H 5N4, Canada

## Abstract

The SARS-CoV-2 Spike receptor-binding domain (S-RBD)/hACE2 interaction represents a challenging protein–protein interaction (PPI) target due to its large, shallow binding interface. Here, in silico alanine mutagenesis guided the structure-based design of constrained peptidomimetics that reproduce key hACE2 recognition elements. α1-helix-derived mimetics (Glu23-Ser44) were stabilized using peptide stapling strategies, while antiparallel β-sheet mimetics (Thr347-Leu359) were generated through head-to-tail macrocyclization incorporating a d-Pro/l-Pro motif. Covalent linkage of these two secondary structure mimetics yielded proteomimetic **28**, designed to preserve the spatial organization of the hACE2 binding interface. Compound **28** selectively binds SARS-CoV-2 S-RBD and disrupts the S-RBD/hACE2 interaction in biophysical and cellular assays, inhibiting pseudovirus entry (IC_50_ of 6.6 μM). Importantly, **28** displays high stability in lung epithelial models (*t*_1/2_ > 24 h) and low epithelial permeability (*P*_app_ = 2.03 × 10^–8^ cm·s^–1^), supporting its potential for intranasal antiviral delivery. These findings establish a proof-of-concept for proteomimetics as promising inhibitors of challenging PPIs.

## Graphical abstract

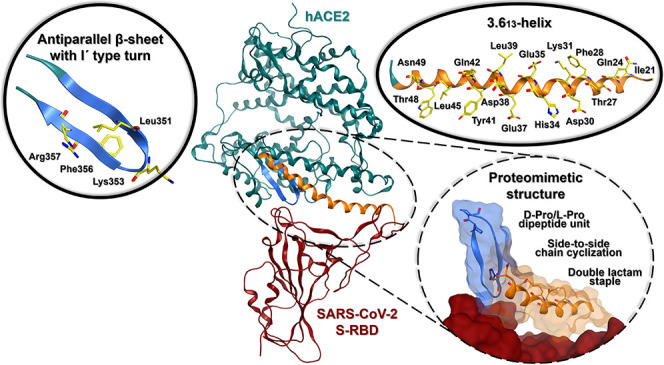

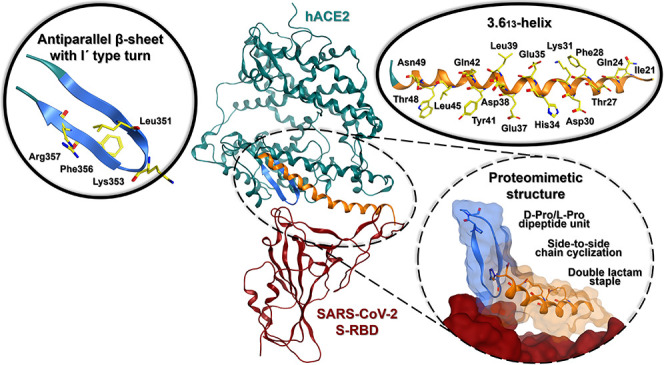

## Introduction

Protein–protein interactions (PPIs)^1^ orchestrate a wide range of essential biological processes, including cellular communication,^2^ signal regulation,^3^ molecular transport^4^ and structural organization.^5^ More recently, interactions between viral and human proteins have been increasingly recognized as critical determinants of viral infection^6–9^ and host immune responses.^10^ The COVID-19 pandemic, driven by the interaction between the severe acute respiratory syndrome coronavirus-2 (SARS-CoV-2) Spike receptor-binding domain (S-RBD) and the human angiotensin-converting enzyme 2 (hACE2), underscored the urgent need for effective PPI inhibitors. To address this challenge, diverse strategies have been developed for the de novo design of protein binders, including empirical screening of large combinatorial libraries,^11–15^ iterative in vitro selections,^16,17^ hotspot-focused computational methods (e.g., molecular docking,^18^ molecular dynamics, machine learning^19–21^ and structure-guided engineering^22^), or finally the repurposing of pre-existing binders, like endogenous soluble ACE2 ^23^ (sACE2), recombinant sACE2,^24,25^ neutralizing antibodies,^26^ and protein scaffolds^27–32^ (Figure 1). All these efforts have been greatly facilitated by the rapid expansion of high-resolution structural,^33–38^ genomic^39–41^ and proteomic^42–44^ data sets, which have transformed previously “undruggable” protein complexes^45–47^ into increasingly tractable therapeutic targets.

**Figure 1:**
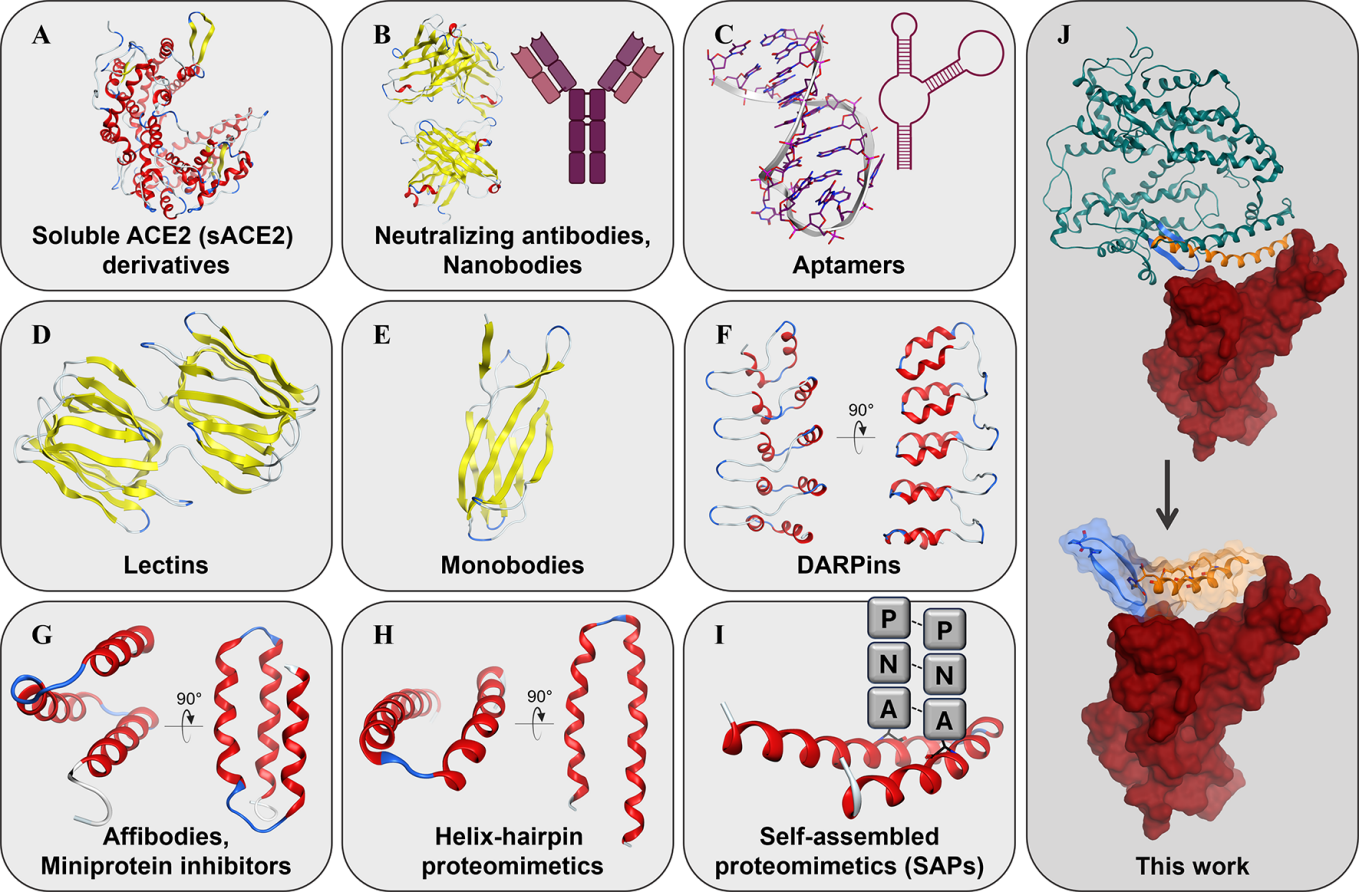
Cartoon representation of diverse scaffolds designed to prevent the SARS-CoV-2/hACE2 PPI. Protein scaffolds are shown as cartoons in white, with helical regions in red, turns in blue, and β-strand/β-sheet motifs in yellow. (A) Soluble ACE2 (sACE2) catalytic domain in open conformation (constructed from PDB ID: 1R42). (B) Class-1 RBD-targeting antibody CB6 ^121^ (constructed from PDB ID: 7C01). (C) An aptamer, a short single-stranded oligonucleotide (DNA or RNA)^16,17^ (constructed from PDB ID: 8TFD). (D) Griffithsin, a dimeric, red algae-derived lectin (constructed from PDB ID: 2NUO). (E) A monobody scaffold derived from the fibronectin type III domain (∼94 amino acids, ∼10 kDa) containing 10 variable positions distributed across two loop regions^15^ (constructed from PDB ID: 3K2M). (F) Designed ankyrin repeat protein (DARPIn) (constructed from PDB ID: 8DW3). DARPin^122^ is a right-handed, solenoid-shaped protein composed of stacked ankyrin repeats (∼33 amino acids, ∼3–4 kDa), whose interaction interface can be engineered to afford high binding affinity without disrupting the tertiary structure of the overall protein. (G) Three-helix bundle miniprotein inhibitor, or affibody (constructed from PDB ID: 8C3E). Affibody^123^ is a three-helix bundle protein commonly derived from the Z-domain of staphylococcal protein A (∼58 amino acids, ∼6–7 kDa), whose two-helix binding surface is engineered at defined positions to create antibody-like molecular recognition. (H) Helix-hairpin (or helix-turn-helix, ∼50 amino acids, ∼5–6 kDa) proteomimetic (constructed from PDB ID: 1BGF). (I) Self-assembled proteomimetic (SAP) (constructed from PDB ID: 1BGF). SAP^15^ is a chimeric peptide–peptide nucleic acids (PNA) conjugate based on a modified two-helix affibody. (J) hACE2-derived proteomimetic (∼30 amino acids, ∼3–4 kDa), mimicking two distinct secondary structures, shown in blue and orange (constructed from PDB ID: 6M0J). Surface of the SARS-CoV-2 S-RBD protein is shown in red, and the N-terminal peptidase domain of hACE2 is depicted in ribbon representation (green).

Surprisingly, various screening assays have identified small-molecule inhibitors (SMIs) capable of disrupting the SARS-CoV-2/hACE2 interaction.^11,14^ However, owing to the extensive and relatively featureless nature of this protein–protein interface, these inhibitors often exhibit limited antiviral potency and specificity, and frequently lack drug-like chemical structures (e.g., organic dyes). PPIs that can be effectively targeted by SMIs are typically classified as class I PPIs,^48,49^ which recognize small protein motifs such as plungingly oriented β-strands, short, disordered protein sequences (3–5 residues), or specific chemical moieties (e.g., phosphotyrosine (pY) or C-terminus).^50^ Class II PPIs,^51^ characterized by the recognition of well-defined secondary structures, are often susceptible to inhibition by conformationally constrained stapled peptides.^52^ In contrast, class III PPIs, such as the SARS-CoV-2 S-RBD/hACE2 interface, feature large, shallow, and discontinuous binding surfaces formed by multiple distal motifs in the primary structure. Effective inhibition of this interface typically requires substantial steric hindrance. Therefore, the most effective approaches to inhibit the SARS-CoV-2/hACE2 interaction rely on large recombinant proteins such as recombinant sACE2 (130 kDa),^53^ neutralizing antibodies (≥140 kDa),^54–58^ nanobodies (∼70 kDa),^26,59,60^ aptamers (∼26 kDa),^16^ lectin Griffithsin (Grft) derivatives (∼12.8 kDa)^27–32^ and macrocycles^61–64^ (Figure 1).

A novel concept of third-class PPI inhibitors has recently emerged in the form of proteomimetics, designed as low- or high-molecular-weight molecules to replicate the structural or functional features of protein surfaces. These compounds mimic the large and complex tertiary structures of proteins while preserving the secondary structures, recognition capabilities, or biological activity of the original protein partner.^65^ Proteomimetics occupy a largely unexplored chemical space that bridges the gap between conventional small molecules and biologics, offering unique opportunities to target challenging PPIs with high affinity and specificity. Several proteomimetics have been reported to target the SARS-CoV-2 S-RBD/hACE2 PPI, including monobody-based inhibitors^66^ (C4-AM2), designed ankyrin repeat proteins^67,68^ (DARPins; SR16m), miniprotein inhibitors^15,69,70^ (AHB1, LCB3), helix-hairpin proteomimetics (SIH-5,^71^ CeSPIACE^22^), and self-assembled proteomimetics^15^ (SAP; SAP_RBD_-5) (Figure 1). These strategies have been validated for their selective target recognition, biological activity in vitro, and promising pharmacological effects in vivo, including the reduction of pulmonary inflammation following SARS-CoV-2 infection in Syrian hamsters.^71^ Collectively, proteomimetics underscore the potential of mimicking natural binding interfaces to achieve high selectivity and neutralization potency. The rational design of next-generation proteomimetics can further benefit from prior advances in structure-guided development of PPI-based inhibitors.

Several research groups have exploited the native α1-helix sequence of hACE2 (Ser19-Gln42), applying peptide stapling strategies to conformationally constrain key hot spot residues in their bioactive α-helix conformation. Early research showed that linear native peptides corresponding to the 6-mer (Glu37-Gn42) and 16-mer (Thr27-Gln42) sequences of the α1-helix displayed only millimolar affinity for the SARS-CoV-2 S-RBD in affinity precipitation assay (K_D_ of 0.53 and 1.36 mM, respectively), and modest antiviral activity in flow cytometry-based inhibition assay (IC_50_ of 2.39 and 1.90 mM, respectively).^72^ Later, systematic *i*, *I* + 7 hydrocarbon stapling of a 23-mer peptide (Ile21-Ser43) resulted in an α-helix mimetic with 72% helicity; however, this construct showed no detectable binding to SARS-CoV-2 S-RBD by fluorescence polarization assay and lacked antiviral activity at concentrations up to 5 mM in pseudovirus inhibition assay.^73^ In contrast, a double *i*, *I* + 4 hydrocarbon-stapled 30-mer peptide (Thr20-Asn49) achieved 80% helicity, demonstrated measurable binding to SARS-CoV-2 S-RBD by surface plasmon resonance (SPR; K_D_ of 2.2 μM) and exhibited antiviral activity in pseudovirus inhibition assays on human lung carcinoma A549 cells (IC_50_ of 2.8 μM).^74^ Finally, studies using single and double triazole-stapled peptides (Gln24-Gln42) suggested that excessive conformational rigidity introduced by double stapling may compromise both binding affinity and antiviral efficacy.^75^

In the present study, an in silico alanine scan (BudeAlaScan) and SPR analyses served as starting points for the structural characterization of the low-affinity SARS-CoV-2 S-RBD/hACE2 PPI. Guided by the spatial distribution and orientation of critical solvent-exposed hotspot residues, we introduced structure-stabilizing elements into two key hACE2-derived sequences – the α1-helix (Glu23-Ser44) and the antiparallel β-sheet (Thr347-Leu359). The weak molecular recognition by the SARS-CoV-2 S-RBD target protein necessitated structural expansion to enhance binding efficiency. Inspired by the nonoverlapping positions, spatial proximity, and complementary orientation of these two well-characterized secondary structures within hACE2, we covalently linked their secondary structure mimetics to generate a rationally designed proteomimetic construct (proteomimetic **28**). To assess its ability to disrupt the SARS-CoV-2 S-RBD/hACE2 interaction, we conducted SPR and bioluminescence-based bioreporter (NanoBiT) assays in parallel. Additionally, a standardized pseudovirus-based neutralization assay was employed to assess inhibitory potency. In summary, our rational de novo design strategy enables the rapid and accurate generation of proteomimetic inhibitors with potential as novel protein binders capable of targeting challenging PPIs.

## Results and Discussion

### Experimental Design, Synthesis, and Structural Analysis

Given that the SARS-CoV-2 S (or SARS-CoV-2 S-RBD) and hACE2 proteins were relatively novel at the time of this study, we first evaluated the functionality of these two naturally interacting proteins by SPR (Figure S1). First, the His-tagged SARS-CoV-2 S protein was immobilized on a Ni-NTA sensor chip, and untagged recombinant hACE2 was injected as the analyte at different concentrations (0.1 μM to 0.00014 μM). The resulting sensorgrams revealed that hACE2 binds to the SARS-CoV-2 S protein with a long residence time, indicative of a low dissociation rate constant and prolonged complex stability. Global 1:1 data fitting yielded an equilibrium dissociation constant (K_D_) of 1.28 nM, with association (*k*_on_ of 8.93 × 10^4^ M^–1^·s^–1^) and dissociation (*k*_off_ of 1.14 × 10^–4^ s^–1^) rate constants, consistent with previously reported values.^76^ These results highlight the inherent challenge of disrupting a PPI governed by such high affinity and structural complementarity, as illustrated by the SARS-CoV-2 S/hACE2 complex.

In silico alanine scan mutagenesis was performed to assess the functional relevance of interfacing residues contributing to the high-affinity interaction between SARS-CoV-2 S-RBD and hACE2 (Figure 2A). Among the 30 identified hotspots, four residues – Asp30, Tyr41, Tyr83, and Lys353 – distributed throughout the interface exhibited binding free energy contributions exceeding 6 kJ·mol^–1^ (Figure 2B). Deep mutational scanning experiments^77^ independently validated several of the hotspot residues identified in our in silico analysis, including Gln24, Phe28, Asp30, Lys31, Asp38, Tyr41, Gln42, Leu45, Lys353, Phe356, and Arg357, providing a strong correlation with our computational prediction. Additional residues (Ile21, Glu37, Trp48, Met82, and Asn330) exhibited weaker but still significant binding contributions^77^, consistent with our results. Based on a predefined threshold (2 kJ·mol^–1^), together with the structural constraints and spatial proximity of the above-mentioned secondary structures (Figure 2C), we focused on constrained peptides mimicking the α1-helix (residues Glu23-Ser44) and the antiparallel β-sheet (Thr347-Leu359) of hACE2. To guide lead optimization, we first synthesized a small library of linear peptides (**1–8**) derived from the well-resolved^36–38^ and functionally characterized^78^ α1-helix of hACE2 (Table S1). Each peptide contained at least four evenly distributed hotspot residues contributing more than 2 kJ·mol^–1^ to the binding free energy, aiming to maximize the probability of disrupting the S-RBD/hACE2 PPI. Far-UV circular dichroism (CD) spectra of the linear compounds **1–8** revealed spectral signatures characteristic of random coil conformations, with helicity values ranging from 4.0% to 18.1% (Figure S2). Although all linear peptides originated from the same hACE2 sequence segment (Ser19-Ser44), variations in length resulted in three-dimensional structural heterogeneity in solution, offering diverse propensities to generate α1-helix mimetics upon subsequent peptide stapling.

**Figure 2:**
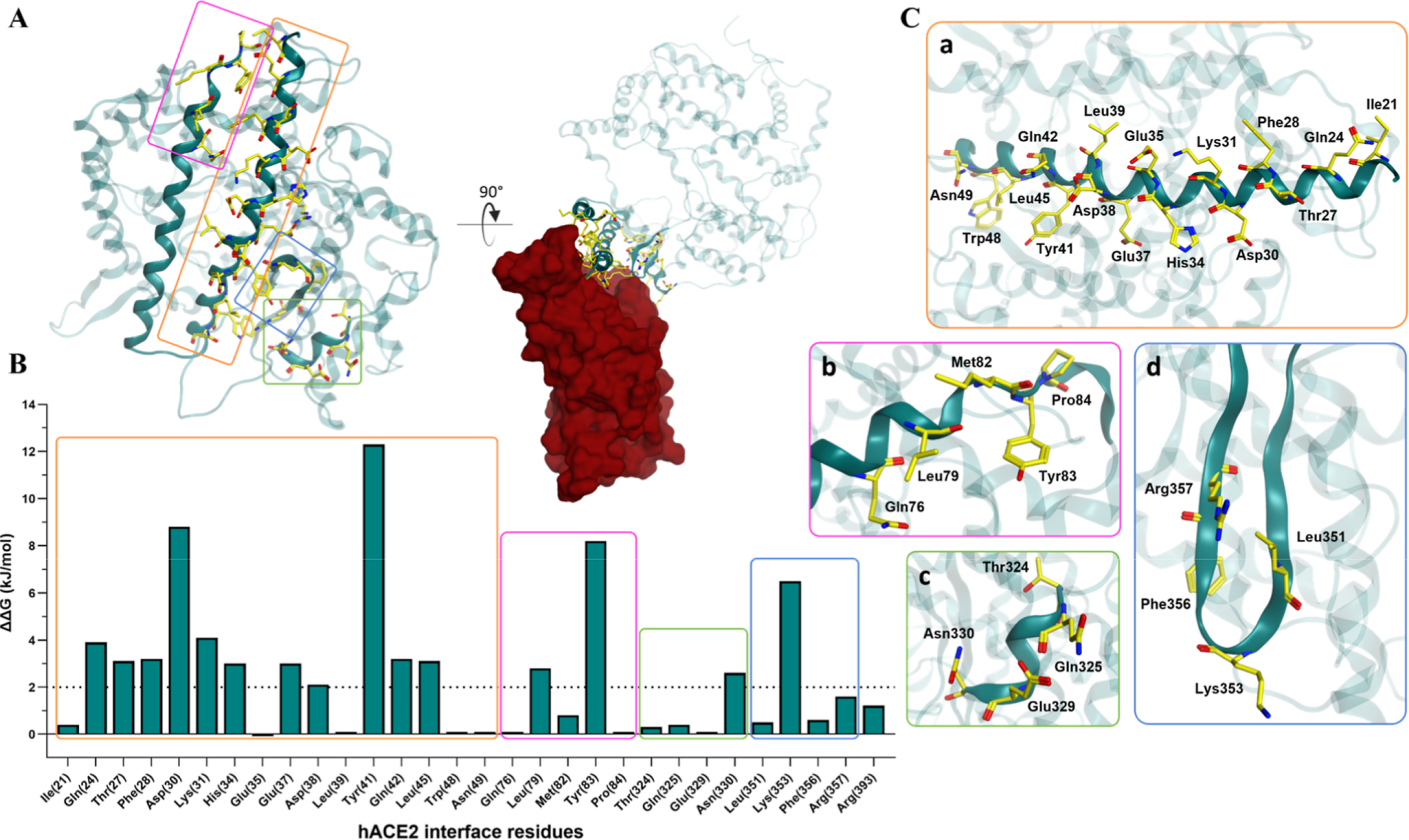
In silico alanine scan (BudeAlaScan^a^) of the hACE2 N-terminal peptidase domain (residues Ser19-Asp615) in complex with the SARS-CoV-2 Spike receptor-binding domain (S-RBD) resulting in four distinct interaction regions. (A) Ribbon representation of the hACE2 N-terminal peptidase domain (green) highlighting the four identified interface secondary structures. A 90-degree rotation of the surface representation of the SARS-CoV-2 S-RBD (red) illustrates its interaction interface with hACE2. (B) Schematic representation of the in silico alanine scan mutagenesis for hACE2 interface residues, showing the binding free energy contribution of each amino acid. Residues corresponding to the four distinct interaction domains are grouped within orange, pink, green, and blue boxes. (C) Close-up views of key interacting residues (yellow) within (a) the hACE2 α1-helix (dark green), (b) the C-terminus of the hACE2 α2-helix (dark green), (c) the hACE2 α3-helix (dark green), and (d) the β3 and β4 sheets (dark green). Structural model based on PDB ID: 6M0J. ^a^ BudeAlaScan is an online alanine scan tool available at https://pragmaticproteindesign.bio.ed.ac.uk/balas/; n.a. not applicable for proteins containing unnatural amino acids.

Guided by the spatial distribution of hotspot residues at the SARS-CoV-2 S RBD/hACE2 interface, potential amino acid substitutions that enhance binding,^77^ and helix-stabilizing side-chain-induced salt bridges (e.g., Glu/Arg pair at the *i*, *i* + 4 positions)^78–80^ while minimizing end effects,^78^ we further explored the hACE2 (23–44) sequence. Peptidomimetics **9–13** were synthesized via *i*, *i* + 4 or *i*, *i* + 7 side-to-side chain cyclizations by olefin ring-closing metathesis (RCM),^81^ lactamization,^82^ or copper­(I)-catalyzed Huisgen 1,3-dipolar azide–alkyne cycloaddition (CuAAC)^83,84^ (Figure 3). These peptide stapling strategies impose conformational constraints that promote α-helical folding, thereby enhancing the ability of the resulting peptidomimetics to disrupt α-helix-mediated PPIs.^83,85,86^ The conformational properties of the synthesized peptidomimetics were assessed by far-UV CD analysis (Figure S3). Helicity values were calculated from the characteristic minimum at 222 nm, as previously described,^78,87^ and the secondary structure assignments were extrapolated using the BestSel online software from the entire far-UV CD spectra. Despite minor differences arising from methodological inputs, both approaches consistently identified compound **9** as the most helical analog, exhibiting up to 84.7% helicity (Figure S3). This observation aligns with our previously reported stapled peptide screening study,^78^ showing that an *i*, *i* + 4 lactam bridge between Lys (N-terminus) and Asp (C-terminus) induces α-helical folding in the hACE2 (30–38) sequence. The peptide sequence was therefore extended to increase the interaction surface, with the goal of improving binding affinity and activity, while promoting additional intramolecular hydrogen bonding to further stabilize the helical conformation.

**Figure 3:**
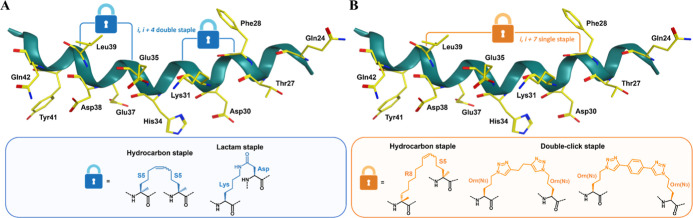
Design of *i*, *i* + 4 double-stapled peptides and *i*, *i* + 7 single-stapled peptides. (A) Schematic representation of *i*, *i* + 4 double-stapled peptides **9** and **10**. The two blue locks indicate the positions of hydrocarbon or lactam staples along the hACE2 α1-helix sequence (Glu23-Ser44; dark green ribbon). Lys and Asp residues were introduced at the N- and C-termini, respectively, to effectively promote the helical conformation, as previously reported.^78^ Four S5 olefinic tethers were used to generate the double-hydrocarbon *i*, *i* + 4 stapled peptide. (B) Schematic representation of *i*, *i* + 7 single-stapled peptides **11**, **12**, and **13**. The orange lock indicates the position of the hydrocarbon or double-click staple along the hACE2 α1-helix sequence (Glu23-Ser44; dark green ribbon). Structural model based on PDB ID: 6M0J. Natural amino acids are abbreviated using their standard three-letter codes. S5 and R8 denote the non-proteinogenic amino acids α-(4-pentenyl)-_L_-Alanine and α-(7′-octenyl)-_D_-Alanine, respectively; Orn­(N_3_) corresponds to δ-azido-_L_-Ornithine.

To design a synthetic β-hairpin mimicking the two antiparallel β3 and β4 strands of hACE2 connected by a well-defined β-turn, we performed a detailed structural analysis of the high-resolution cryo-EM structure of the SARS-CoV-2 S-RBD/hACE2 (PDB ID: 6M0J). This examination enabled structural classification according to previously established criteria^88,89^ (Figure 4A,B). Our analysis showed that the hACE2-derived antiparallel β-sheet, spanning from residues Thr347 (N-terminus) to Leu359 (C-terminus), consists of a polypeptide chain of four and five residues, extending approximately 20 Å in length (Figure 4C). This structure is stabilized by an extensive hydrogen-bonding network between the two nondistorted strands running in opposite directions, which reinforces the β-hairpin conformation and constrains the formation of dihedral angles to 56.5° (Φ_*i*+1_), 38.4° (ψ_*i*+1_), 68.0° (Φ_*i*+2_), and 17.3° (ψ_*i*+2_) (Figure 4D,E). Considering the high intrinsic flexibility of β-turns and the typical variability of φ and ψ dihedral angles up to ±30°,^88,89^ we can assume that the antiparallel β-sheet studied adopts an I′ type β-turn.

**Figure 4:**
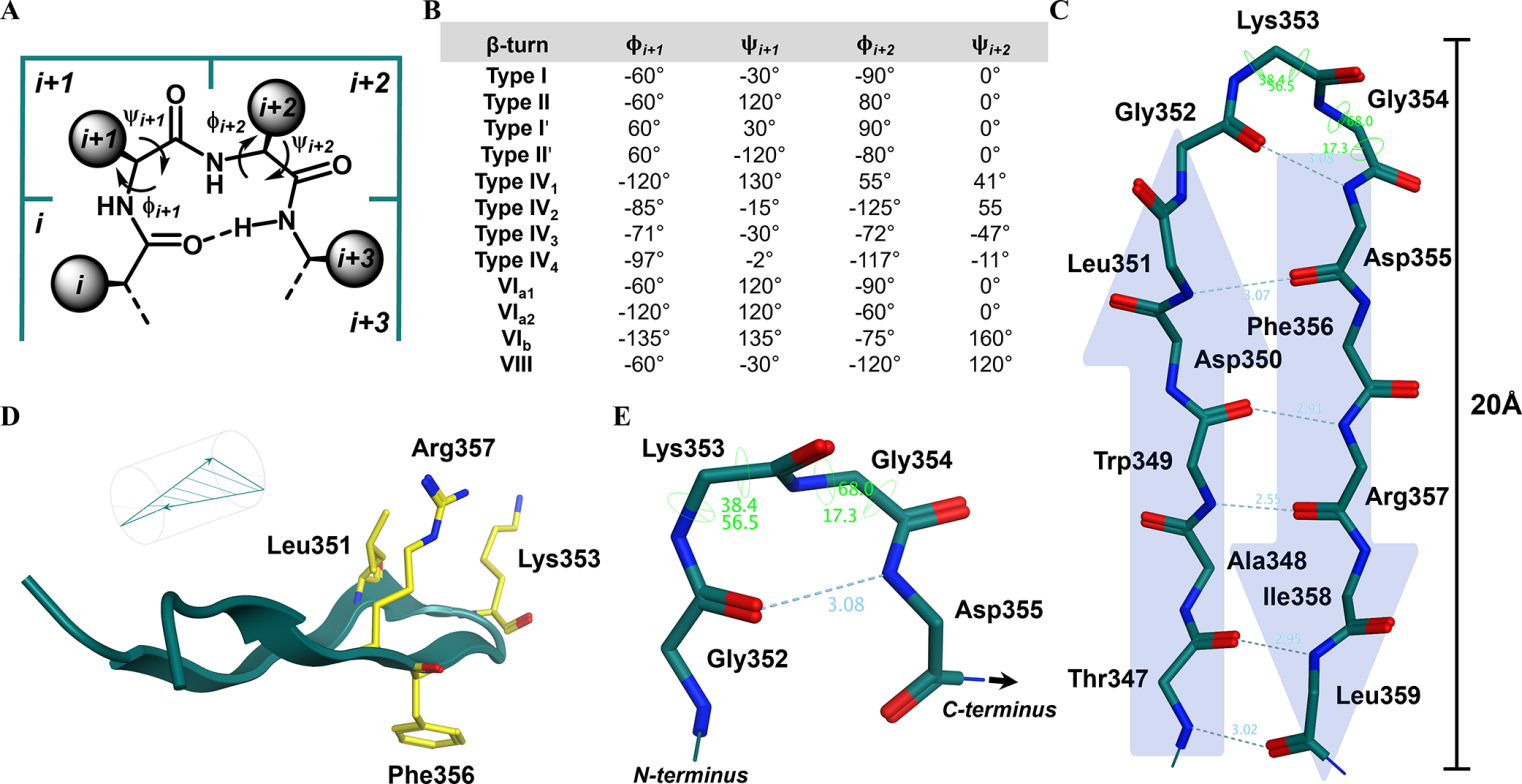
Structural characterization of the hACE2 antiparallel β-sheet (Thr347-Leu359) from the SARS-CoV-2 S-RBD/hACE2 cryo-EM complex. (A) Schematic representation of a hydrogen-bonded β-turn illustrating the four backbone dihedral angles. (B) β-turn classification based on Φ_*i*+1_, ψ_*i*+1_, Φ_*i*+2_, and ψ_*i*+2_ dihedral angles.^88^ (C) Backbone representation of the hACE2 antiparallel β-sheet (Thr347-Leu359) with hydrogen bonds (dashed blue lines) and interatomic distances (Å). The four backbone dihedral angles (degrees) are indicated by green circles. (D) Structure of the right-handed twisted β-sheet conformation of hACE2 with interface residues on the peptide shown in ball-and-stick mode. (E) Close-up view of the β-turn forming the hACE2-derived β-sheet with the highlighted *i*, *i* + 3 hydrogen bond (dashed blue line) and the four dihedral angles (green circles), as determined using MOE software. Structural model based on PDB ID: 6M0J.

Reproducing the hACE2 antiparallel β-hairpin presents a key challenge due to the need to maintain a flexible β-turn with a characteristic hydrogen-bond pattern between the backbone carbonyl group of Gly352 at position *i* and the backbone amide of Asp355 at position *i* + 4 (Figure 4E). Substituting the entire peptide backbone within the β-turn with a traditional small-molecule scaffold, such as benzodiazepine or glucose,^90^ would likely compromise peptide recognition by SARS-CoV-2, particularly at Lys353, which is one of the highest energetic contributors at this position. To address this limitation, we incorporated turn-inducing amino acids (d-Pro/l-Pro)^91^ into various ACE2-derived linear sequences to promote β-hairpin formation, combined with head-to-tail macrocyclization on the opposite side of the scaffold (Figure 5 and Scheme 1). Specifically, the d-Pro/l-Pro nucleating turn motif was positioned away from the native β-turn region, unlike previous designs,^92^ in order to maintain rigid antiparallel strand alignment while preserving the native turn formed by residues Gly352, Lys353, Gly354, and Asp355.

**Scheme 1:**
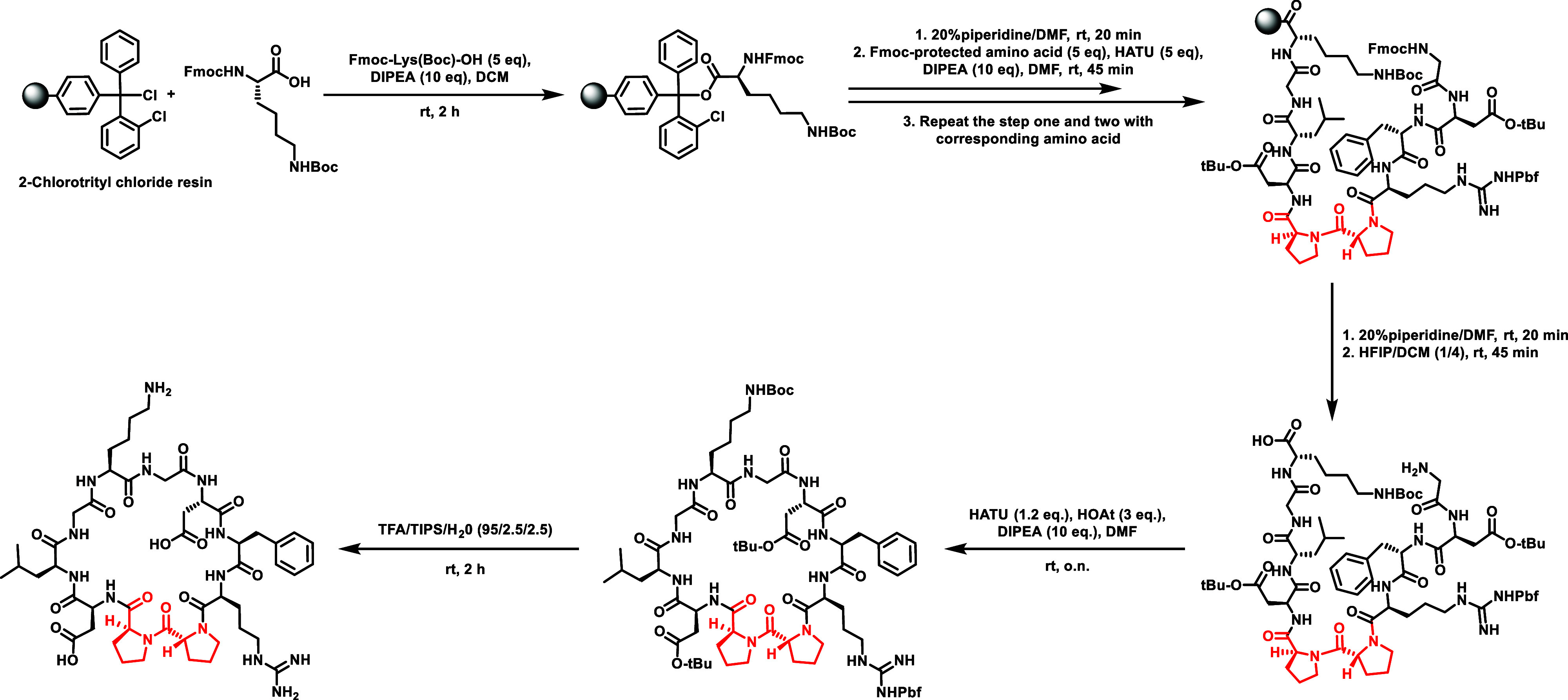
Synthesis of the β-Hairpin Mimicking Peptide **14** ^a^

a Fmoc-Lys­(Boc)–OH (Nα-fluorenylmethoxycarbonyl-Nε-tert-butoxycarbonyl- *l* -lysine), DIPEA (*N,N*-diisopropylethylamine), DCM (dichloromethane), DMF (dimethylformamide), HATU (*O*-(7-azabenzotriazol-1-yl)-*N*,*N*,*N*′,*N*′-tetramethyluronium hexafluorophosphate), HFIP (hexafluoroisopropanol), HOAt (1-hydroxy-7-azabenzotriazole), TFA (trifluoroacetic acid), TIPS (triisopropylsilane).

As a first step, linear peptide precursors were synthesized on 2-chlorotrityl chloride resin via standard *N*-(9-fluorenylmethoxycarbonyl) (Fmoc) solid-phase chemistry. The incorporation of a heterochiral d-Pro/l-Pro unit promotes β-hairpin formation by constraining the backbone and bringing the N- and C-termini into close proximity; therefore, solid phase synthesis was initiated at the opposite side of the template, at the Gly–Lys junction for peptide **14**. Second, head-to-tail macrocyclization was performed in solution, followed by side-chain deprotection and peptide purification, yielding a small library of β-hairpin mimetics **14–22** with diversified sequences (Figure S4). Far-UV CD spectra of these peptides were analyzed using the BestSel online software to quantify secondary structure content. To selectively mimic a right-handed twisted β-sheet with an I′ type turn while limiting disordered conformations (>50%), only macrocycles **14** and **16** were considered for further proteomimetic design. A second generation of β-hairpin mimetics **23–26** was synthesized by substituting Leu351 in the native sequence with l- or d-propargylglycine. The second-generation compounds **25** and **26** display CD features characteristic of β-sheet structures, including a positive band near 195 nm and a broad negative band in the 210–220 nm region^93^ (Figure S5A). In contrast, the CD spectra of macrocycles **23** and **24** indicate a mixture of β-sheet and β-turn conformations, consistent with the fact that eight of the ten residues participate in turn formation (Figure S5A). Nevertheless, the CD spectrum of **23** closely resembles that of a 10-mer gramicidin S analog,^94^ providing strong evidence that **23** adopts a β-hairpin conformation. BestSel analysis further supports that **23** retains the ability to form a right-handed twisted β-sheet conformation (Figure S5B).

**Figure 5:**
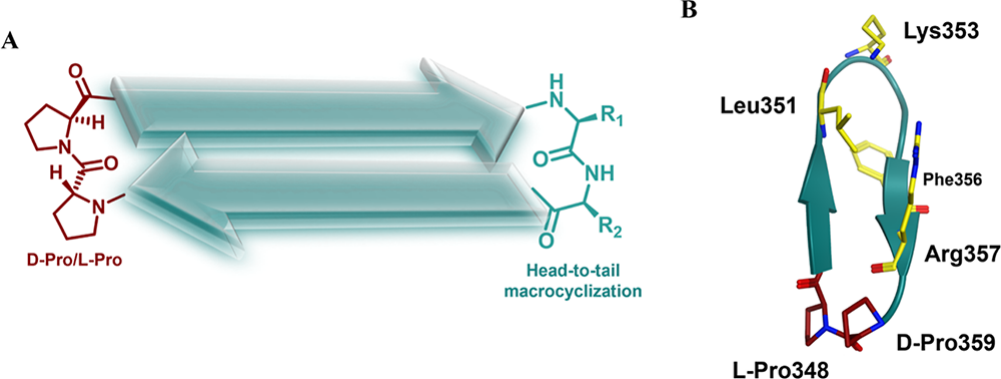
β-hairpin structure-based design for hACE2-derived macrocyclic peptides. (A) Schematic representation of an antiparallel β-sheet mimetic employing a d-Pro/l-Pro dipeptide unit and a head-to-tail macrocyclization technique. R_1_ and R_2_ denote variable amino acid residues situated within the β-turn region of the native protein. (B) Molecular modeling of the macrocyclic peptide **16** derived from the native hACE2 sequence (Ala348-Leu359). Structural model based on PDB ID: 6M0J.

To further validate the β-sheet content of analog **23**, 1D and 2D total correlation spectroscopy (TOCSY) nuclear magnetic resonance (NMR) spectra were recorded, and proton chemical shifts were assigned (Figure S6). The chemical shift index was subsequently used to estimate the β-strand propensity of individual residues. The average amide proton chemical shifts were shifted downfield relative to random coil reference values.^95^ In addition, residues Phe, Arg^4^, Asp^7^, and propargylglycine^8^ of **23** displayed positive Hα chemical shift deviations compared with random coil values,^95^ consistent with significant β-sheet content in this region. The β-hairpin character progressively increases for residues located closer to the nucleating turn formed by the d-Pro/l-Pro motif. In contrast, residues located at the opposite side (Gly^1^, Gly^9^, and Lys^10^) exhibit chemical shifts closer to random coil reference values.^95^ This increased flexibility in solution, as indicated by the recorded NMR data in water, may facilitate the adoption of the bioactive conformation upon binding to the SARS-CoV-2 S-RBD interface. Based on these structural characteristics, **23** was selected as the lead β-hairpin mimetic for subsequent proteomimetic design.

**Figure 6:**
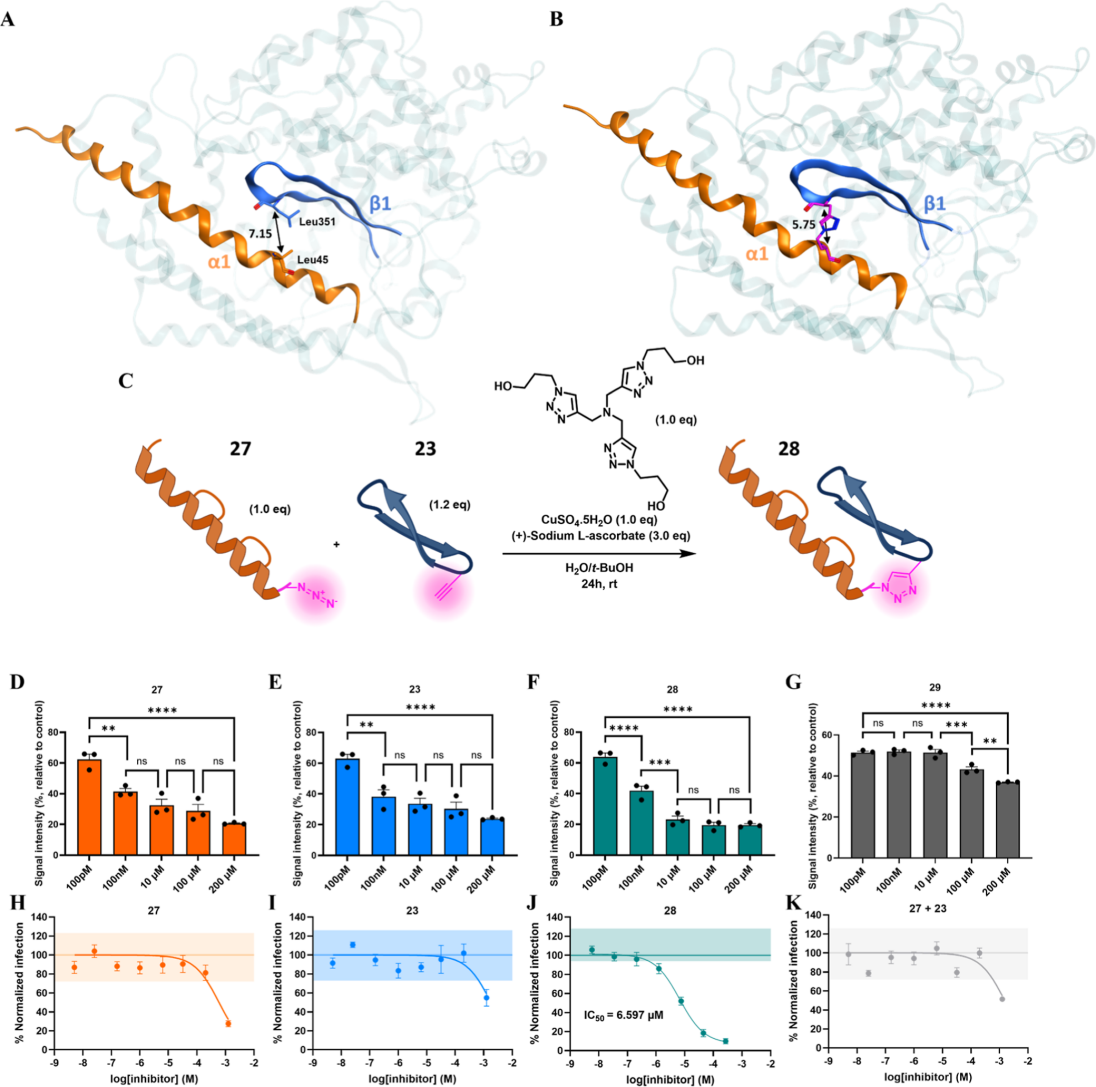
Rational design, synthesis, and in vitro biological activity of the proteomimetic 28, compared with its parent subunits (27 and 23). (A) Structural proximity between the α1-helix (orange) and the antiparallel β1-sheet (blue) of hACE2 (transparent green) before covalent linkage. Ribbon representation of the α1-helix and the antiparallel β1-sheet within the peptidase domain of hACE2, highlighting the interstructural distance (in Å). (B) Energy-minimized ribbon representation of the same region following covalent conjugation, showing the α1-helix (orange), antiparallel β1-sheet (blue), and triazole linkage (bold pink). (C) Application of the CuAAC bioorthogonal reaction used to synthesize proteomimetic **28**. Parent compounds **27** (α-helix mimetic, orange) and **23** (β-hairpin mimetic, blue) are shown with their respective azide and alkyne functional groups (pink). Detailed synthetic procedures are provided in the Materials and Methods section. Quantification of SARS-CoV-2 S-RBD/hACE2 interaction disruption by **27** (α-helix mimetic, D), **23** (β-hairpin, E), **28** (proteomimetic, F) and **29** (scrambled proteomimetic, G) using the NanoBiT assay. Bars represent mean values from three independent experiments (*n* = 3), each performed in triplicate; individual means are shown as circles. Data are represented as mean ± standard error of the mean (SEM). Statistical analyses were performed using one-way ANOVA with Tukey’s multiple comparison correction. For all analyses, significance levels: ****p < 0.0001; ***p < 0.001; **p < 0.01; n.s., not significant (*p* ≥ 0.05). Dose–response curves generated from increasing concentrations of **27** (H), **23** (I), **28** (J), and an equimolar mixture of **27** and **23** (K) in the pseudovirus-based neutralization assay. Experiments for panels H, I, and K were conducted in duplicate (*n* = 2), and those for panel J in triplicate (*n* = 2). Data are expressed as mean ± SEM (error bars). CuSO_4_·5H_2_O (copper­(II)­sulfate pentahydrate), *tert*-butanol (*t*-BuOH).

### Proteomimetic Design and Activity Assessment

The proteomimetic design was guided by structural analysis of the SARS-CoV-2 S-RBD/hACE2 complex (PDB ID: 6M0J). The structural proximity of 7.15 Å between the amide backbone of the α1-helix and the antiparallel β1-sheet of hACE2 allowed the design of a side-to-side chain cyclization between two nonproteinogenic amino acids bearing size-restricted side-chains, strategically positioned relative to the interfacial residues (Figure 6A). A CuAAC reaction was selected for covalent linkage due to its orthogonality, biocompatibility, and ability to constrain the two peptidomimetic components within an approximate spatial proximity of 5.75 Å (Figure 6B). The parent compounds **27** (derived from the α-helix mimetic compound **9**) and **23** (derived from the β-hairpin mimetic compound **14**), generated by rational substitution at Ser44→L-Dap­(N_3_) and Leu351→l-Propargylglycine, were used to synthesize the novel proteomimetic **28**, preserving high structural similarity to the corresponding secondary structures of hACE2 (Figure 6C). Moreover, these residues occupy positions distinct from the key binding hotspots (e.g., Asp30, Lys31, Tyr41, and Lys353), thereby minimizing interference with critical intermolecular interactions. Far-UV CD spectra of **27** featured a characteristic maximum near 195 nm and two minima at 208 and 222 nm, consistent with an α-helical structure and reaching a helicity of 50.8%, whereas **23** exhibited spectral features of a right-twisted β-sheet with a β-structure content of 18.5% (Figures S5 and S7). The CD spectrum of peptidomimetic **28** exhibits only a slight rightward shift to those of parent compounds **27** and **23**, indicating overall preservation of their secondary structural features. Notably, compound **28** displays spectral characteristics consistent with α-helical content, including a maximum between 190 and 195 nm and two negative minima at approximately 208 and 222 nm, as previously reported.^96^ The more pronounced second minimum also suggests the presence of turn formation, which in peptidomimetic systems typically appears as a broad negative band around 230 nm.^97^ Interestingly, the far-UV CD spectrum of the scrambled linear sequence **29** of proteomimetic **28** showed a strong minimum at 206 nm, indicative of a random coil conformation, with a calculated helicity of 10.8% (Figure S7).

Subsequently, the NanoBiT-based biosensor assay evaluating the ability of compounds **27**, **23**, and **28** to disrupt the SARS-CoV-2 S-RBD/hACE2 interaction at 200 μM demonstrated a significant reduction in luminescence signal compared to the positive control (Figure S8). The modest differences in inhibitory potency observed among the compounds likely reflect their structural diversity. To further characterize these effects, a dose–response NanoBiT assay was conducted across a concentration range of 200 μM, 100 μM, 10 μM, 100 nM, and 100 pM (Figure 6D–G). Both parent compounds (**27** and **23**) and the peptidomimetic **28** exhibited significant disruption of S-RBD/hACE2 interaction even at 100 pM, while at 200 μM, the loss of reporter signal attained less than 25%. In contrast, the scrambled proteomimetic **29** showed neither comparable potency nor a discernible dose-dependent effect within the same concentration range. These dose-dependent results support that **28** specifically disrupts the SARS-CoV-2 S-RBD/hACE2 interaction, with minimal influence from steric hindrance due to its molecular size (MW of 3.82 kDa for **29** vs 3.78 kDa for **28**).

The proteomimetic compound **28**, along with its corresponding peptidomimetic components **27**, **23**, and the scrambled derivative **29**, was further characterized using SPR. A concentration range of 0.14 μM–300.00 μM (3-fold serial dilution) of each compound was flowed over sensor chip surfaces immobilized with three different viral surface proteins: the SARS-CoV-2 (2019-nCoV) S-RBD-Fc recombinant protein, the SARS-CoV-2 S-RBD-Fc recombinant protein bearing the L452R and E484Q mutations, and the Influenza B hemagglutinin (HA2 Subunit) (Figures S9–S14). The HA2 Subunit, used as a negative control, showed no increase in binding signal for any of the compounds, indicating selective interaction with SARS-CoV-2 S-RBD (Figures S10C,F, S11C,F, S12C,F, S13C,F). Among the four tested compounds, only **27** and **28** exhibited a dose-dependent binding response to the immobilized SARS-CoV-2 S-RBD. Although the relatively short target occupancy governed by a high dissociation constant (*k*_off_), and the variability observed across 24 replicate injections limited kinetic analysis, the reproducible dose-dependent binding profiles obtained in two independent experiments support a specific and consistent interaction between compounds **27** or **28** and the immobilized SARS-CoV-2 S-RBD (Figures S10–S13).

Compounds **27**, **23**, and **28** were then assessed for their neutralization capacity against the SARS-CoV-2 pseudovirus encoding a green fluorescent protein (GFP) reporter, enabling fluorescence-based quantification of infection. Compared with the NanoBiT assay, which measures a soluble binary interaction between engineered proteins, the pseudovirus assay offers a more physiologically relevant assessment of antiviral activity by capturing the multivalent and conformational contributions of the trimeric S protein during viral entry. Under biosafety level 2 (BSL-2) containment, the pseudovirus was incubated with ACE2-expressing HEK293T cells transfected with transmembrane serine protease 2 (TMPRSS2), allowing viral entry via the plasma membrane pathway without subsequent replication. Variability in infection levels is represented as a shaded area around the 100% infection baseline (Figure 6H–K). Neutralization potency was determined from normalized pseudoviral inhibition response curve and expressed as the half-maximum inhibitory concentration (IC_50_), as previously described.^78^ A commercially available monoclonal anti-SARS-related coronavirus 2 S-RBD-mFc fusion protein (S-RBD-mFc; IC_50_ = 0.15 nM) and camostat mesylate were included as positive controls (Figure S15). Compounds **27** and **23** exhibited measurable inhibitory activity only at the highest concentration tested, achieving 72 and 45% inhibition, respectively. Importantly, the equimolar mixture of both subunits corresponding to the proteomimetic **28** did not result in a synergistic antiviral effect beyond 51% inhibition (Figure 6K). The lack of additive or equivalent response suggests that the two subunits fail to reach the spatial arrangement required for synergistic binding and likely engage the same antiviral mechanism, resulting in competitive steric hindrance. In contrast, the covalently linked peptidomimetic **28** effectively inhibited SARS-CoV-2 pseudovirus entry in a concentration-dependent manner, with an IC_50_ of 6.6 μM and reaching 90% inhibition at the highest concentration tested. These findings indicate that, without covalent linkage, the individual subunits exhibit excessive spatial freedom, preventing simultaneous engagement with the target. Collectively, these results support the potential of compound **28** as a direct and potent inhibitor of the SARS-CoV-2 S-RBD/hACE2 interaction.

### In Vitro Pharmacokinetic Characterization

Vaccines, antibodies,^54–58^ mini-proteins,^69,98^ and other emerging COVID-19 therapeutics^26,59,60^ are high-molecular weight biomolecules with a large number of cleavable peptide bonds that generally exhibit low membrane permeability and limited oral bioavailability, whereas smaller protein- or peptide-based agents may additionally suffer from relatively short half-lives (*t*_1/2_). As a result, these agents typically require parenteral administration. Recently, substantial progress has been made toward intranasal delivery approaches, including the development of advanced inhalation devices^99^ and formulation strategies^100^ designed to minimize systemic side effects while maximizing local target exposure. Due to its high-molecular weight (MW > 3.5 kDa), large topological polar surface area (TPSA of 1648.64 Å^2^, donors = 45, acceptors = 57), and conformational rigidity, compound **28** was identified as a promising candidate for noninvasive pulmonary administration. However, for nasally administered therapeutics intended to act locally, diffusion across the lung epithelium and subsequent systemic exposure are not desired. Therefore, we performed an in vitro pharmacokinetic permeability study to evaluate the permeability of compound **28** using a standardized in cellulo air–liquid interface (ALI) model of the pulmonary epithelium,^101–103^ along with assessments of its stability in plasma and human lung adenocarcinoma epithelial (Calu-3) cells (Figures S16 and S17).

The stability of the compounds was first evaluated using the Calu-3 cell line, which closely mimics the human airway epithelium^104,105^ (Figure S16A). The results indicated that compounds **27**, **23**, **28**, and **29** all exhibited *t*_1/2_ exceeding 24 h, suggesting slow degradation by proteases expressed in Calu-3 cells^106^ (Table 1).

**Table 1:** Comparative Analysis of in Vitro Pharmacokinetics, Including Calu-3 Cell-Based Stability, Permeability and Plasma Stability^a^

compound	calu-3 stability *t*_1/2_ (h)	permeability P_app_ × 10^–6^ (cm·s^–1^)	plasma stability *t*_1/2_ (h)
**27**	>24	BQL^*a*^	9.2 ± 2.0
**23**	>24	5.8 ± 0.4	7.4 ± 1.2
**28**	>24	0.02 ± 0.01	8.7 ± 1.2
**29**	>24	0.8 ± 0.1	3.6 ± 0.7

a BQL = below the lower limit of quantification.

To maximize lung exposure while minimizing systemic absorption, we next evaluated the permeability of the compounds across the airway epithelial barrier using an in cellulo ALI model^105,107^ (Figure S17). This model provides a physiologically relevant environment characterized by mucus-secreting Calu-3 monolayers and less restrictive tight junctions compared to the liquid–liquid interface (LLI) configuration.^101,102^ Interestingly, the proteomimetic **28** demonstrated high lung stability (*t*_1/2_ > 24 h) and very low apparent permeability (*P*_app_ = 2.03 × 10^–8^ cm·s^–1^) relative to the control propranolol (*P*_app_ = 2.71 × 10^–4^ cm·s^–1^). Additionally, **28** displayed lower permeability than its β-hairpin parent **23** (*P*_app_ = 5.8 × 10^–6^ cm·s^–1^) and the scrambled compound **29** (*P*_app_ = 7.8 × 10^–7^ cm·s^–1^). It is worth noting that extended residence time in the pulmonary environment could potentially promote peptide aggregation, which may impair biological activity and decrease airway flow.^108^ Although peptide **28** exhibited low permeability, the integrity of the Calu-3 monolayer pulmonary epithelium was maintained for **28** and all other tested compounds, as indicated by the low intra- and intercompound variability in transepithelial electrical resistance (TEER) measurements before and after experiments (Figure S17C). For the double-stapled α-helix mimetic **27**, concentrations below the limit of quantification (BLQ) were detected in the basolateral compartment 2 h postdeposition, consistent with very low permeability and possible surfactant requirements to avoid aggregates or deposition in the larger airways.

Since an optimal therapeutic effect requires a balance between prolonged local exposure and controlled pulmonary epithelial diffusion, we further assessed their systemic stability. Resistance to proteolysis in blood was measured by incubating the compounds in rat plasma and recording peptide degradation over time (Figure S16B). The α-helix mimetic **27** exhibited a *t*_1/2_ of 9.2 h, while the β-hairpin analog **23** demonstrated a shorter *t*_1/2_ of 7.4 h (Table 1). The resulting proteomimetic **28** displayed intermediate stability (*t*_1/2_ of 8.7 h), whereas the scrambled linear peptide **29** had the lowest plasma stability (*t*_1/2_ of 3.6 h). These trends suggest that increased conformational rigidity enhances proteolytic resistance, consistent with prior reports demonstrating the improved stability of stapled peptidomimetics compared to their proteinogenic counterparts.^109,110^

Overall, the lead proteomimetic **28** demonstrates low metabolic transformation by Calu-3 cell–expressed proteases, high plasma stability and low permeability in the in cellulo model, collectively supporting its potential for prolonged local retention and effective target exposure within the lung environment.

## Conclusion

The COVID-19 pandemic continues to represent a public health emergency of international concern (PHEIC), with the World Health Organization (WHO) maintaining close surveillance of emerging SARS-CoV-2 subvariants and ongoing global efforts to develop effective treatments and prophylactics. Several studies have emphasized the critical role of hACE2 in mediating viral entry across SARS-CoV-2 variants, highlighting that natural viral evolution disrupting the SARS-CoV-2 S-RBD/hACE2 complex is unlikely, as it would entail a detrimental reduction in virulence.^111^ Moreover, numerous SARS-CoV-2 mutations are well tolerated or even enhance hACE2 binding,^112^ suggesting that emerging variants may acquire increased affinity for hACE2 and, by extension, for hACE2-derived proteomimetics. Therefore, therapeutics that exploit the high-affinity interaction between SARS-CoV-2 and hACE2 are of significant interest,^113,114^ particularly given the extensive high-resolution structural insights obtained early in the pandemic.^34,37,115–117^

In this study, BudeAlaScan analysis was performed to identify key hot-spot residues contributing most strongly to the SARS-CoV-2/hACE2 interaction (ΔΔ*G* up to 12.5 kJ·mol^–1^). The spatial arrangement of these residues guided the design of a proteomimetic aimed at reproducing the essential secondary structure elements of hACE2 involved in viral recognition. Structure-constraining strategies, such as RCM, lactamization, and CuAAC, among others,^78^ were selected based on their synthetic feasibility, well-established reaction conditions, and high-yielding purification profiles. This approach led to the identification of a double-lactam-stapled peptide **9**, exhibiting high helicity (84.7%), which was subsequently converted into the proteomimetic parent compound **27** via rational substitution of Ser44 with L-Dap­(N_3_).

To mimic the antiparallel β-sheet structure of the hACE2 fragment (Thr347-Leu359), a d-Pro/l-Pro dipeptide unit was incorporated to facilitate efficient head-to-tail macrocyclization, yielding β-hairpin mimetic **23**, which maintained the desired right-twisted β-sheet conformation (18.5%). Finally, CuAAC was employed to generate potent proteomimetic **28**, whose initial binding to the SARS-CoV-2 S-RBD was confirmed by SPR. The compound’s size-independent activity was demonstrated by concentration–response curves in the NaNoBit assay and its antiviral potency in a pseudovirus assay (IC_50_ of 6.6 μM). Notably, proteomimetic **28** demonstrated strong potential for IN administration, showing high stability under a lung-like environment (*t*_1/2_ > 24 h) and low apparent permeability across the airway epithelial barrier (*P*_app_ = 2.03 × 10^–8^ cm·s^–1^). Although proteomimetic **28** has a higher molecular weight than orally administered antivirals, IN delivery could overcome this limitation while providing a more flexible and versatile dosing strategy.

Altogether, this proof-of-concept study highlights the promise of proteomimetics as an emerging therapeutic modality to target challenging PPIs. In this context, compound **28** represents a compelling lead candidate for further optimization toward the development of next-generation antiviral agents enabled by innovative peptidomimetic medicinal chemistry approaches. Future efforts will focus on optimizing these proteomimetics and evaluating their antiviral activity against authentic SARS-CoV-2 under BSL-3 conditions.

## Experimental Section

### Reagents

The TentaGel S RAM resin with a loading capacity of 0.23 mmol/g was purchased from Rapp Polymere (Tübingen, Germany). All commercially available *N*-(9-fluorenylmethoxycarbonyl)-(Fmoc-)­protected amino acids and coupling reagents were purchased from Combi Blocks (San Diego, CA, USA), Chem-Impex (Wood Dale, IL, USA), or Matrix Innovation (Quebec, QC, Canada) at the highest purity available. (*S*)-*N*-Fmoc-α-(4-pentenyl)­alanine (S5), (*R*)-*N*-Fmoc-2-(7′-octenyl)­alanine (R8), and Grubbs first-generation catalyst were obtained from Sigma-Aldrich (St. Louis, MO, USA). Trifluoroacetic acid (TFA) and *N*,*N*-diisopropylethylamine (DIPEA) were purchased from Chem-Impex (Wood Dale, IL, USA). Palladium (*O*)­tetrakis­(triphenyphosphine) (Pd­(PPh_3_)_4_) was purchased from Strem Chemicals (Newburyport, MA, USA). *N*,*N*-Dimethylformamide (DMF) and diethyl ether (Et_2_O) were purchased from Fisher Scientific (Hampton, NH, USA) and used as received. All other reagents and solvents, including acetonitrile (ACN), dichloromethane (DCM), methanol (MeOH), were purchased from Sigma-Aldrich (St. Louis, MO, USA). Monoclonal anti-SARS-related coronavirus 2 S-RBD-mFc fusion protein (produced in vitro) was obtained from Sino Biological (Beijing, China; catalog no. 40592-MM57, NR-53796). Camostat mesylate was purchased from APExBIO (Houston, TX, USA; catalog no. B2082). All cell culture media and supplements used for cell maintenance and for cell-based experiments including bioreporter-based neutralization, pseudovirus-based neutralization, in vitro stability, plasma stability, and in vitro permeability were obtained from Life Technologies Ltd. (Paisley, UK), unless otherwise stated. The human lung adenocarcinoma epithelial (Calu-3) cell line was purchased from American Type Culture Collection (catalog no. HTB-55). For the SPR assay, SARS-CoV-2 S recombinant protein with His tag (catalog no. 10549-CV), SARS-CoV-2 (2019-nCoV) S-RBD recombinant protein with Fc tag (catalog no. 40592-V02H, LC14MC2602) and SARS-CoV-2 S-RBD (L452R, E484Q) protein with Fc tag (catalog no. 40592-V02H, LC15JU1601) were purchased from Sino Biological (Beijing, China). Influenza B (B/Florida/4/2006) Hemagglutinin Protein (HA2 Subunit) with Fc tag (Catalog No. 11053-V01H2, LCL07OC2803) was also obtained from Sino Biological (Beijing, China) and used as a negative control.

### Synthesis of α-Helix Mimetics

#### Solid Phase Peptide Synthesis (SPPS)

All linear peptides were synthesized on a 0.1 mmol scale using a Symphony X Peptide Synthesizer from GYROS PROTEIN Technologies (Uppsala, Sweden). The TentaGel S RAM resin with a loading capacity of 0.23 mmol/g was directly weighed into 45 mL plastic reaction vessel. Coupling of proteinogenic Fmoc-protected amino acids (5.0 equiv) was achieved by treatment with *N*,*N′*-diisopropylcarbodiimide (DIC; 5 equiv) and Oxyma Pure (5.0 equiv) at room temperature for 45 min. Nonproteinogenic Fmoc-protected acids S5 and R8 were coupled twice for 1 h. The Fmoc protecting group was removed using 20% piperidine in DMF for 10 min. This step was repeated twice. Following each coupling and deprotection cycle, the resin was subjected to five washes with DMF. Agitation of the resin, reagents, and solvents was achieved by nitrogen bubbling. Upon completion of the linear sequence, the peptides were washed five times with DCM according to the standard procedure recommended by the Symphony X Peptide Synthesizer, promoting resin swelling and facilitating recovery from the 45 mL reaction vessels.

#### N-Terminal Acetylation

Following Fmoc deprotection with 20% piperidine in DMF, on-resin acetylation was performed using acetic anhydride (3.0 equiv) and DIPEA (4.5 equiv) in DMF (7 mL for 0.1 mmol of resin) for 20 min on an orbital shaker operating at 120 rpm. The resin was then washed with DMF (3 × 5 mL), DCM (3 × 5 mL), iPrOH (1 × 2 mL), and DMF (3 × 5 mL) prior to other reactions or peptide cleavage and global deprotection.

#### Lactam Staple Formation on Resin

Peptides containing Fmoc-Lys­(Alloc)–OH and Fmoc-Asp­(OAll)–OH underwent on-resin selective removal of allyl-based protecting groups prior to side-to-side chain lactamization. Deprotection was performed with Pd­(PPh_3_)_4_ (0.1 equiv) and *N*,*N*-dimethylbarbituric acid (4.0 equiv) in anhydrous DCM (2 mL for 0.1 mmol of resin) at 25 °C for 1 h, with repetition as required based on UPLC-MS analysis of resin-cleaved aliquots. The resin was then washed with DCM (2 × 5 mL), DMF (2 × 5 mL), 0.5% diethyldithiocarbamate in DMF (2 × 5 mL) and DMF (2 × 5 mL). Lactam staple formation was performed on-resin using BOP (1.5 equiv) and DIPEA (2.0 equiv) in DMSO/NMP (2:8, v/v; 2 mL per 0.1 mmol of resin). The reaction progression was monitored by using the Kaiser test.^118^ BOP (1.5 equiv) and DIPEA (5.0 equiv) in DMSO/NMP (2:8, v/v; 2 mL per 0.1 mmol of resin) were added to the resin when we observed a positive (i.e., blue) Kaiser test; the resin was then agitated overnight at room temperature. If the Kaiser test was negative (i.e., yellow), the resin was washed with DMF (3 × 5 mL), DCM (3 × 5 mL), iPrOH (1 × 1 mL), DCM (3 × 5 mL) and DMF (3 × 5 mL); consequent peptide synthesis or peptide cleavage/global deprotection, purification and characterization were then conducted according to the procedures described below.

#### Hydrocarbon Staple Formation on Resin

Peptides containing S5 or R8 residues were stapled on-resin via ring-closing metathesis (RCM). Following SPPS and N-terminal acetylation, the resin was suspended in anhydrous DCE, filtered, followed by addition of Grubbs first-generation catalyst (0.2 equiv), resuspended in DCE, and heated at 70 °C for 1 h under microwave irradiation. Reaction progress was monitored by UPLC-MS after cleavage of a resin aliquot. When an acetylated linear peptide was detected, the resin was washed with DCE (3 mL) and a second round of RCM was performed with fresh catalyst. The resin was then washed with DMF (2 × 5 mL), DCM (2 × 5 mL), iPrOH (1 × 5 mL), DCM (2 × 5 mL) and DMF (2 × 5 mL) prior to further peptide synthesis or peptide cleavage/global deprotection, purification, and characterization.

#### Double Triazole Staple Formation on Resin

Double triazole stapling was performed on-resin via copper­(I)-catalyzed Huisgen 1,3-dipolar azide–alkyne cycloaddition according to a modified literature procedure.^119^ The peptide-resin was preswelled with DMF and treated with copper­(I) iodide (CuI; 3.0 equiv), DIPEA (3.0 equiv), and a dialkynyl linker (50.0 equiv) in ACN. After stirring the solution in a nitrogen atmosphere at room temperature for 24 h, additional CuI (3.0 equiv) and DIPEA (3.0 equiv) were added, and the reaction mixture was stirred overnight or until completion. Reaction progress was monitored by UPLC-MS after cleavage of a resin aliquot. The resin was then washed with DMF (2 × 5 mL), DCM (2 × 5 mL), iPrOH (1 × 5 mL), DCM (2 × 5 mL), and DMF (2 × 5 mL) prior to peptide cleavage/global deprotection, purification, and characterization.^120^

#### Peptide Cleavage and Side-Chain Deprotection

Peptides were cleaved from the resin with TFA/TIPS/H_2_O (95:2.5:2.5, v/v/v; 1 mL for 30 mg of resin) for 2 h on an orbital shaker at 120 rpm. The resin was subsequently filtered, and the crude peptide was precipitated in cold Et_2_O. The suspension was centrifuged at 4 °C for 10 min at 2000 rpm in an Allegra TM 6R Centrifuge (Beckman Coulter Life Sciences, Indianapolis, IN, USA). The supernatant was discarded, and the remaining white solid was dissolved in a mixture of H_2_O and ACN containing 0.1% TFA and lyophilized overnight in a freeze-dryer (LABCONCO, Kansas City, MO, USA).

### Synthesis of β-Sheet Mimetics

#### Solid Phase Peptide Synthesis (SPPS)

Peptides were synthesized using SPPS on 2-chlorotrityl chloride resin (100–200 mesh, dry, 1.0 mmol/g) using standard Fmoc chemistry. Accordingly, the coupling of the first amino acid was performed in DCM using an Fmoc-protected amino acid (5.0 equiv) and DIPEA (10.0 equiv) for 2 h at room temperature. Subsequently, the resin was capped with 3 mL of DCM/MeOH/DIPEA (7:2:1, v/v/v) for 20 min to limit unreacted sites. The Fmoc protecting group was removed by filtration upon mixing the resin with 20% piperidine in DMF for 10 min. This step was repeated twice. Subsequent couplings of proteinogenic Fmoc-protected amino acids (5.0 equiv) were carried out using *O*-(7 azabenzotriazol-1-yl)-*N*,*N*,*N*′,*N*′-tetramethyluronium hexafluorophosphate (HATU; 5.0 equiv) and DIPEA (10.0 equiv) in DMF at room temperature for 45 min. After each coupling and deprotection step, the resin was washed with DMF (2 × 5 mL), DCM (2 × 5 mL), iPrOH (1 × 5 mL), DCM (2 × 5 mL), and DMF (2 × 5 mL). Upon completing the linear sequence, the resin was dried under vacuum. A small amount of resin was then cleaved with TFA for 10 min, and the peptide was analyzed by UPLC-MS.

#### Peptide Cleavage

Peptides were cleaved from the resin using a 30% solution of hexafluoroisopropanol (HFIP) in DCM (1 mL for 30 mg of resin) while shaken on an orbital shaker at 120 rpm for 45 min. The mixture was subsequently filtered, and the solution was evaporated in vacuo on a rotary evaporator. If necessary, this step was repeated once more for 45 min using the same cleavage solution, followed by a thorough wash of the resin with DCM (10 mL for 1 g of resin). The cleaved resin was discarded, and the solution was evaporated in vacuo on a rotary evaporator once again. The remaining acidic solution not removed on rotary evaporator was evaporated using a SpeedVac Savant SC250EXP concentrator (SpectraLab Scientific Inc., Markham, ON, Canada).

#### Peptide Cyclization in Solution and Side-Chain Deprotection

Cyclization of linear β-sheet mimicking peptides bearing standard protecting groups (1.0 equiv) was achieved in nonstandard coupling conditions using HATU (1.2 equiv), HOAt (3.0 equiv) and DIPEA (10.0 equiv) in DMF. After overnight agitation at room temperature, the reaction mixture was subjected to UPLC-MS analysis to confirm the head-to-tail macrocyclization. Upon completion, DMF was removed in vacuo, and acid-labile side-chain protecting groups were deprotected with TFA/TIPS/H_2_O (95:2.5:2.5, v/v/v) at room temperature for 2 h. Following evaporation of the cleavage solution, the resulting orange slurry was dissolved in a mixture of H_2_O and ACN depending on peptide solubility, filtered through a 0.22 μm PTFE chromatography syringe filter, and purified according to the described protocol.

### Synthesis of Proteomimetic **28**

For proteomimetic synthesis, the copper­(I)-catalyzed Huisgen 1,3-dipolar azide–alkyne cycloaddition was performed to covalently link the α-helix and the β-sheet mimetics. A solution of purified azido-peptide (1.0 equiv) and purified alkyne-peptide (1.2 equiv) in a 1:1 H_2_O/*t*-BuOH ratio (1 mL/mg peptide) was degassed with nitrogen for 15 min, followed by the addition of CuSO_4_·5H_2_O (1.0 equiv), tris­(3-hydroxypropyltriazolylmethyl)­amine (1.0 equiv), and sodium l-ascorbate (3.0 equiv). After stirring the solution in a nitrogen atmosphere at room temperature for 24 h, the reaction mixture was subjected to UPLC-MS analysis to confirm completion of the reaction. If unreacted azido-peptide was detected, CuSO_4_·5H_2_O (1.0 equiv) was added to the mixture and stirred until completion. Then, the reaction mixture was lyophilized and purified via preparative HPLC-MS to yield the final proteomimetic. If the analytical UPLC exhibited a broad peak near the expected proteomimetic peak, ethylenediaminetetraacetic acid (EDTA; 1.0 equiv) was added to the lyophilized crude prior to purification.

### Peptide Purification

The previously lyophilized peptide was dissolved in a 1.8 mL mixture of H_2_O and ACN depending on peptide solubility and filtered through a 0.22 μm PTFE chromatography syringe filter. The peptides were purified using Waters preparative HPLC-MS (column XSELECTTM CSHTM Prep C18 (19 × 100 mm) packed with 5 μm particles, UV detector 2998, MS SQ Detector 2, Sample manager 2767, and a binary gradient module). The purified intermediate or the final product was lyophilized overnight in a freeze-dryer (LABCONCO, Kansas City, MO, USA). Final peptides were isolated with a purity exceeding 95% for use in subsequent experiments (Tables S1–S6).

### Peptide Characterization and Data Analysis

All of the data were analyzed using GraphPad Prism (version 10.3.1) (San Diego, CA, USA). All the peptides were analyzed using a Waters UPLC-MS system (column Acquity UPLC CSHTM C18 (2.1 × 50.0 mm) (Agilent Technologies, Santa Clara, CA, USA) packed with 1.7 μm particles). A 5–95% gradient of ACN and H_2_O containing 0.1% TFA was used over the course of 2.5 min at a flow rate of 1 mL/min at 25 °C. Purity of all compounds was ≥95% by UPLC-MS using UV chromatogram peak integration. High-resolution mass spectra were acquired on a maXis 3G orthogonal mass spectrometer (ESI-QqTOFMS) (Bruker Daltonik; Bremen, Germany) using electrospray ionization in positive (or negative) ion mode. Each compound was dissolved in DCM (or MeOH) and then diluted 10-fold in MeOH. Each solution was then infused individually into the ESI-QqTOFMS using a syringe pump at a flow rate of 5 μL/min. Mass spectra were recorded over a range of *m*/*z* 50–1200, and external calibration was performed using a 0.5 mM sodium formate solution.

### Circular Dichroism

Circular dichroism measurements were carried out following the protocol previously reported.^78^

### Molecular Modeling

Molecular modeling studies were conducted using the Molecular Operating Environment (MOE) software from the Chemical Computing Group, version MOE2022.02. The crystal structure of the SARS-CoV-2 S-RBD bound with ACE2 was obtained from the Protein Data Bank (PDB ID: 6M0J). Prior to molecular modeling, all loaded 3D coordinates were corrected. First, polar hydrogens and partial charges were added using the “Protonate 3D” function of MOE. For the protonation process, a temperature of 300 K, a concentration of 0.1 mol/L salt in the solvent, and a pH of 7 were specified. Second, any missing heavy atoms, alternate geometries, or other crystallographic artifacts were corrected using the “QuickPrep” function. “Measure distance” tool of MOE allowed measurement of distances (in Angstroms (Å)) between backbone atoms. “Measure dihedrals” function was used to measure Φ_*i*+1_, ψ_*i*+1_, Φ_*i*+2_, and ψ_*i*+2_ dihedral angles. To construct a schematic representation of the antiparallel β-sheet mimetics featuring the d-Pro/l-Pro unit and a head-to-tail macrocyclization, the ligand was generated in ChemDraw Ultra (CambridgeSoft, MA, USA) and imported into the MOE platform from its simplified molecular input line entry system (SMILES) notation. Subsequent energy minimization using the AMBER10.Extended Hückel Theory (EHT) force field was applied to optimize the ligand’s geometry, yielding a stable, low-energy conformation suitable for structural representation and analysis. The proteomimetic compound was generated using the “Builder” tool, including the triazole moiety as a covalent attachment between the α1-helix and the antiparallel β1-sheet of hACE2.

### Nuclear Magnetic Resonance (NMR) Measurements and Data Analysis

The NMR sample was prepared in 10% deuterium oxide (D_2_O) (Sigma-Aldrich, Poole, UK), 40 mM dipotassium phosphate (K_2_HPO_4_) pH 6.8, 40 mM potassium chloride (KCl), and 0.2% sodium azide (NaN_3_). 2D ^1^H–^1^H TOCSY (mixing time 50 ms) and 1D ^1^H spectra were acquired at 25 °C on a Bruker Avance operating at a ^1^H frequency of 600 MHz and equipped with a cryogenic probe (TCI H&F–C/N-D-05 2 XT) and *Z*-axis pulsed-field gradients. The NMR data were processed using Topspin 3.6.2 (Bruker) and analyzed using CcpNmr software (version 3.3.2.2) (Leicester, UK). Chemical shifts (δ) are reported in parts per million (ppm).

### Surface Plasmon Resonance (SPR)

#### Evaluation of SARS-CoV-2 S + hACE2 Binding

His-tagged 2019-nCoV S protein was immobilized onto an Ni-NTA sensorchip (GE Healthcare, Chicago, IL, USA) to a level of ∼800 response units (RUs) using a Biacore X100 (GE Healthcare, Chicago, IL, USA) and a running buffer composed of 10 mM 2-[4-(2-hydroxyethyl)­piperazin-1-yl]­ethanesulfonic acid (HEPES) pH 8.0, 150 mM sodium chloride (NaCl), and 0.05% Tween 20. The serial dilutions of purified and untagged ACE2 were injected, ranging in concentration from 0.00014 nM to 0.1 nM. The surface was regenerated using 5 mM sodium hydroxide (NaOH; 30 s, 30 μL/min). A stabilization period of 240 s in running buffer was maintained between consecutive analyte injections to ensure consistent baseline levels. The sensorgrams were double-referenced by subtracting the reference signal and the reference sensorgram in the absence of protein. The resulting sensorgrams were fitted based on a 1:1 binding model using the “single-curve analysis” function in the Anabel 2.0 software, and resulting values and standard errors of fit were averaged from duplicates.

#### SPR Kinetic Determination in Concentration-Dependent Response Assay for Compounds **27**, **23**, **28**, and **29**

SPR anti-Fc antibody was immobilized to an SPR chip HC_30 M (serial number: 02022023.11a, Expiration date: 2026/10/31) to an average coupling response unit of 1814.8 RUs using the Carterra LSA (Salt Lake City, UT, USA) (Figure S9B). To the resulting anti-Fc antibody, SARS-CoV-2 (2019-nCoV) S-RBD recombinant protein with Fc tag, SARS-CoV-2 S mutated (L452R, E484Q) RBD recombinant protein with Fc tag, and Influenza B hemagglutinin (HA2 Subunit) with Fc tag were noncovalently captured (Figure S9A), with average couplings reported in Figures S9C,D,E. Fc-tag proteins did not give concentration-dependent responses, suggesting saturating conditions (RUs > 500). These ligands were subsequently distributed into two identical 96-well plates using an automated pipetting system programmed specifically for this experiment. Ligand distribution was based on a design sheet to ensure the random allocation of ligands across the plate. Peptide samples were prepared at a concentration of 0.005 μg/μL in 1 × HBSTE supplemented with 0.5 mg/mL bovine serum albumin (BSA). Then, samples underwent serial dilutions ranging from 0.1372 μM, 0.4115 μM, 1.2346 μM, 3.7037 μM, 11.1111 μM, 33.3333 μM, 100.0000 μM, to 300.0000 μM. Each analyte was prepared in duplicate for replicate injections. Prior to analyte injection, the sensor chip surface was conditioned with running buffer (1 × HBSTE + 0.5 mg/mL BSA). Each binding cycle included three main steps: ligand capture (15 min), analyte injection (8 concentrations with 8 min association and 8 min dissociation per concentration), and surface regeneration (2 × 30 s). A stabilization period of 1 min in running buffer was maintained between consecutive analyte injections to ensure consistent baseline levels. Between immobilizations, no significant loss of capture efficiency was detected, confirming the integrity of the chip across all binding cycles. Kinetic information could not be extracted from the data obtained, due to variability. Two representative sensorgrams from two independent experiments (n = 2) for each immobilized surface and for each tested peptide sample are represented in Figures S10–S14.

### Bioreporter-Based Neutralization Assay (NanoBiT)

Cell maintenance, generation of biosensors, and neutralization assay protocols specific to the NanoBiT neutralization assay were performed according to our previously reported protocols.^78^

#### Bioreporter-Based Inhibition Assay Data Analysis

The inhibitory data were plotted in RLUs using GraphPad Prism (version 10.3.1). Each result represents three independent experiments, measured in triplicate each time, and the data are expressed as means ± SEM (error bars). The means of more than two groups were compared using one-way ANOVA with Tukey’s multiple-comparison correction. For all bioreporter-based inhibition assay analyses, ****p < 0.0001; ***p < 0.001; **p < 0.01; n.s., not significant (*p* ≥ 0.05).

### Pseudovirus-Based Neutralization Assay

Lentiviral vector design, cell maintenance, generation of pseudoviral particles, and the pseudovirus-based neutralization assay were performed according to our previously reported protocols.^78^

#### Pseudovirus-Based Inhibition Assay Data Analysis

The fluorescence data (RFUs) were converted to percentages by normalizing them to the control without infection (DMEM), set as 0%, using GraphPad Prism (version 10.3.1); we presumed that the pseudovirus-only samples represented maximum infection (100%). Experiments represented in Figures 6H,I,K and S15B were replicated in duplicate (*n* = 2), and the experiments in Figures 6J and S15A were repeated in triplicate (*n* = 2). The data are expressed as means ± standard error of the mean (SEM) (error bars).

### In Vitro Stability Assay

#### In Vitro Calu-3–Based Stability Assay

The Calu-3 cells were maintained in Eagle’s minimum essential medium (EMEM) supplemented with 10% fetal bovine serum (FBS), 1% penicillin/streptomycin, and 1% GlutaMAX Supplement 100X. All the cells were cultured at 37 °C under a humid atmosphere containing 5% carbon dioxide (CO_2_). The cell culture medium was changed three times per week. After reaching 80–90% confluence in the culture flasks, the cells were detached by treatment with trypsin/EDTA 0.25%, seeded in 96-well plates and grown to confluence. Before performing the in vitro stability assay, cells were washed twice with phosphate-buffered saline (PBS), then incubated in duplicate with 100 μM of the inhibitor in EMEM supplemented with 0.1% BSA. Cell media were collected at five time points i.e., 0, 30 min, 1, 6, and 24 h, followed by the addition of 75% v/v ACN in H_2_O to the samples at a sample-to-solvent ratio of 1:1.11. Samples were vortexed for 10 s and then centrifuged at 13,000 rpm for 20 min at 4 °C. The supernatant was collected and stored at −80 °C. UV spectra of the samples were analyzed using a PDA eλ UV–vis detector on a Waters UPLC-MS system (column Acquity UPLC CSHTM C18 (2.1 × 50.0 mm) (Agilent Technologies, Santa Clara, CA, USA) packed with 1.7 μm particles). A 5–95% gradient of ACN and H_2_O containing 0.1% TFA was used over the course of 2.5 min at a flow rate of 1 mL/min at 25 °C. Data from 3 independent experiments (*n* = 3), each performed in triplicate, are expressed as means ± SEM (error bars).

#### Plasma Stability Assay

Plasma stability assays were performed according to our previously reported protocol.^78^ Rat plasma was obtained from animals in accordance with protocols approved by the Animal Care Committee of the Université de Sherbrooke (2022–3440) and in compliance with Canadian Council on Animal Care policies and ARRIVE guidelines. Time-dependent plasma stability profiles of compounds **27**, **23**, **28**, and **29**, are presented in Figure S16B, with angiotensin II (AngII) and apelin-13 used as reference controls, and corresponding in vitro plasma half-lives (*t*_1/2_) reported in Table 1. Data from 3 independent experiments (*n* = 3), each performed in triplicate, are expressed as means ± SEM (error bars).

### In Vitro Lung Permeability Assay

#### Cell Maintenance for in Vitro Permeability Assay

The Calu-3 cells were maintained in EMEM supplemented with 10% FBS, 1% penicillin/streptomycin, and 1% GlutaMAX Supplement 100X. All cells were cultured at 37 °C under a humid atmosphere containing 5% CO_2_. The cell culture medium was changed three times per week. After reaching 80–90% confluence in the culture flasks, the cells were detached by treatment with trypsin/EDTA 0.25% and seeded at a density of 4 × 10^5^ cells/cm^2^ on polycarbonate membranes (0.4 μm pores, 0.12 cm diameter inserts) in Costar 12-well cell culture plates. Sufficient volumes of cell culture medium were added to the apical (500 μL medium/insert) and basolateral compartments (1500 μL medium/feeder tray) on the day of seeding the cells on membranes. The cell culture medium in the apical compartment was changed 8 h postseeding, and the cell culture medium was changed every 3 days. To establish an air–liquid interface (ALI) interface, the medium from the apical compartment was removed 9 days postseeding.

#### Transepithelial Electrical Resistance (TEER) Experiments

The TEER experiments were carried out using a Millicell-ERS 2 Epithelial Volt-Ohm Meter (Sigma-Aldrich, ON, CA). The TEER of Calu-3 monolayers grown on Costar 12-well cell culture plates was measured using the chopstick method. Baseline resistance was determined only in wells containing inserts without cells. The baseline resistance was subtracted from the experimentally determined values in wells with cells and expressed in Ω cm^2^. The TEER evolution was plotted from the means of independent TEER measurements for each 12-wells of a Costar transwell plate, measured in duplicate (*n* = 2). TEER was monitored pre- and postdelivery of compounds in the permeability assay to analyze TEER recovery. Results were expressed in columns that represent an average ±SD of independent TEER measurements for each 12-wells of a Costar transwell plate, measured in duplicate. Comparison between pre- and postexperimental values was conducted using one-way ANOVA with Tukey’s multiple comparison correction. For all analyses, n.s., not significant.

#### In Vitro Permeability Assay

Prior to conducting the permeability assay, the cell layers were rinsed twice with assay buffer (Hanks’ Balanced Salt Solution (HBSS) containing 0.01 M HEPES), prewarmed at 37 °C, followed by TEER measurement (Millicell ERS-2 Voltohmmeter, Sigma-Aldrich, ON, CA). TEER values superior to 1000 Ω cm^2^, measured on the day of the experiment were considered suitable for the assay. The permeability of peptide compounds was assessed by pipetting 500 μL of the diluted compound solution to the apical compartment, while 1200 μL of freshly prewarmed assay buffer were added to the basolateral side. The positive control was carried out in parallel by adding 500 μL of propranolol solution to the apical compartment, instead of the peptide solution. The negative control consisted of adding 0.5 μL of DMSO to the acceptor well containing 500 μL of prewarmed assay buffer. The 12-well cell culture plates with Calu-3 cells were incubated at 37 °C in a humidified 5% CO_2_ atmosphere throughout the experiment. Samples (100 μL) were withdrawn from the acceptor and/or donor wells at predetermined time points (0, 5, 15, 30, and 120 min) and the withdrawn volume was replaced with fresh assay buffer. The collected samples were centrifuged at 2500 rpm for 5 min at 4 °C and the supernatants were then subjected to UPLC-MS. A 5–95% gradient of ACN and H_2_O containing 0.1% TFA was used over 2.5 min at a flow rate of 1 mL/min at 25 °C. The amount of each compound in the sample was quantified as the ratio of the value at *t = x* relative to the value at *t* = 0 for the peptide. Following sample collection, the cells were gently washed with assay buffer, and the TEER was measured as previously described. TEER values superior to 1000 Ω cm^2^ at the end of the experiment were considered sufficient for maintained barrier integrity. The pre- and postexperiment TEER measurements were plotted on a graph to detect any potential disruption of the cell layer.

### Human Studies

This research did not involve human participants.

## Supplementary Material





## Notes

The Supporting Information is available free of charge at Supporting Information data include results for test compounds and corresponding controls, and demonstrate the establishment and validation of the respective assay models. SPR binding kinetics of recombinant hACE2 to captured His-tagged SARS-CoV-2 S protein (Figure S1); far-UV CD spectra (Figure S2–S5 and **S7**); TOCSY characterization data of β-hairpin mimetic **23** (Figure S6); NanoBiT-based biosensor assay responses at 200 μM (Figure S8); SPR-based concentration–response assay establishment on Carterra LSA, including schematic, anti-Fc capture validation, and Fc-tagged protein immobilization (Figure S9); SPR sensorgrams showing ligand injections over SARS-CoV-2 S-RBD-Fc (WT and L452R/E484Q) and Influenza B HA2-Fc immobilized surfaces (Figure S10–S14); neutralization potency of control compounds in a pseudovirus-based assay, including dose–response analysis of an anti-SARS-related coronavirus 2 S-RBD-mFc fusion protein and single-dose inhibition by camostat mesylate (Figure S15); Calu-3 cell-based and plasma stability curves (Figure S16); permeability data from the human bronchial epithelial cell model (Figure S17); sequences and characterization data of linear native hACE2-derived peptides, α1-helix staple peptides, β-hairpin mimetics, proteomimetic **28**, and scrambled control derivative (Table S1–S6); UPLC-MS and NMR spectra. These data present results for the test compounds and corresponding controls, and demonstrate the establishment and validation of the respective assay models (PDF)
Molecular formulas and biological information on compounds (CSV) Accession Codes. PDB ID: 6M0J (CSV)

## Abbreviations

Papp: apparent permeability
ALI: air–liquid interface
BSL-2: biosafety level 2
BSL-3: biosafety level 3
BudeAlaScan: in silico alanine scan
Calu-3: human lung adenocarcinoma epithelial cell line
CD: circular dichroism
COVID-19: coronavirus disease 2019
CuAAC: copper­(I)-catalyzed Huisgen 1,3-dipolar azide–alkyne cycloaddition
cryo-EM: cryogenic electron microscopy
DARPIn: designed ankyrin repeat protein
DIPEA: *N*,*N*-diisopropylethylamine
DCM: dichloromethane
DMF: dimethylformamide
Fmoc-Lys­(Boc)–OH: Nα-fluorenylmethoxycarbonyl-Nε-*tert*-butoxycarbonyl-l-lysine
GFP: green fluorescent protein
Grft: Griffithsin
HA: influenza hemagglutinin
hACE2: human angiotensin-converting enzyme 2
HATU: hexafluorophosphate azabenzotriazole tetramethyl uronium
HFIP: hexafluoroisopropanol
HOAt: 1-hydroxy-7-azabenzotriazole
IN: intranasal administration
*K*_D_: equilibrium dissociation constant
L-Dap­(N_3_): β-azido-L-Alanine
LLI: liquid–liquid interface
L-Orn­(N_3_): δ-azido-L-Ornithine
L-Pra: L-Propargylglycine
MW: molecular weight
NanoBiT: bioluminescence-based bioreporter
NMR: nuclear magnetic resonance
PHEIC: public health emergency of international concern
pY: phosphotyrosine
PPIs: protein–protein interactions
RCM: ring-closing metathesis
S-RBD: Spike receptor-binding domain protein
S-RBD-mFc: monoclonal anti-SARS-related coronavirus 2 S-RBD-mFc fusion protein
SAP: self-assembled proteomimetic
SMIs: small-molecule inhibitors
sACE2: soluble ACE2
SARS-CoV-2: severe acute respiratory syndrome coronavirus-2
SARS-CoV-2 S: severe acute respiratory syndrome coronavirus-2 Spike
SPR: surface plasmon resonance
*t*_1/2_: half-live
*t*-BuOH: *tert*-butanol
TEER: transepithelial electrical resistance
TMPRSS2: transmembrane serine protease 2
TFA: trifluoroacetic acid
TIPS: triisopropylsilane
TPSA: topological polar surface area
WHO: world health organization
