# Supplementary material for: Structure-Guided Design of Proteomimetics Targeting the SARS-CoV‑2 S‑RBD/hACE2 Interface
Source: J Med Chem. 2026 Jun 15;69(12):14156–73. doi: 10.1021/acs.jmedchem.5c03450 (PMC13313058; doi:10.1021/acs.jmedchem.5c03450)

Supporting Information

Structure-Guided Design of Proteomimetics Targeting the SARS-CoV-2 S-RBD/hACE2 Interface

Sára Ferková[a], Agathe Fayolle[a], Olivier Boisvert[b], Ulrike Froehlich[a], Marie-Édith Nepveu-Traversy[a], Pierre Lavigne[b], Michel Grandbois[a], Philippe Sarret[a*], and Pierre-Luc Boudreault[a*]

[a]Department of Pharmacology and Physiology, Faculty of Medicine and Health Sciences, Institut de Pharmacologie de Sherbrooke, Université de Sherbrooke, 3001 12e Avenue Nord, Sherbrooke QC, J1H 5N4, Canada

[b]Department of Biochemistry and Functional Genomics, Faculty of Medicine and Health Sciences, Université de Sherbrooke, 3001 12e Avenue Nord, Sherbrooke, QC, J1H 5N4, Canada

[*]Corresponding authors:
Philippe Sarret (Philippe.Sarret@USherbrooke.ca)
Pierre-Luc Boudreault (Pierre-Luc.Boudreault@USherbrooke.ca)

Contents

1. Supplementary figures **S4**

Figure S1. Surface plasmon resonance (SPR) sensorgram showing the binding kinetics for recombinant hACE2 with captured His-tagged SARS-CoV-2 S protein. S4

Figure S2. Helicity assessment of native hACE2 α1-helix-derived linear compounds. S5

Figure S3. Helicity assessment of *i, i + 4* and *i, i + 7* hACE2 α1-helix-derived stapled peptides. S6

Figure S4. β-hairpin mimetics of the hACE2 antiparallel β-sheet (Thr347-Leu359) with secondary structure assignments. S7

Figure S5. β-hairpin mimetics of the hACE2 antiparallel β-sheet (Thr349-Ile358) with secondary structure assignments. S8

Figure S6. Total correlation spectroscopy (TOCSY) nuclear magnetic resonance (NMR) spectral regions recorded for β-hairpin mimetic **23**. S9

Figure S7. Helicity assessment (%) of the proteomimetic parent compound **27** (α-helix mimetic), the proteomimetic **28**, and the scrambled sequence **29** of proteomimetic **28**. S10

Figure S8. SARS-CoV-2 S-RBD/hACE2 NanoBiT-based biosensor assay for comparative analysis of PPI disruption capacities. S11

Figure S9. SPR-based concentration-response assay performed on the Carterra LSA. S12

Figure S10. SPR sensorgrams for compound **27**. S13

Figure S11. SPR sensorgrams for compound **23**. S14

Figure S12. SPR sensorgrams for compound **28**. S15

Figure S13. SPR sensorgrams for **29**. S16

Figure S14. SPR sensorgrams generated upon buffer chip conditioning. S17

Figure S15. Neutralization potency of control agents in the pseudovirus-based neutralization assay. S18

Figure S16. Calu-3 cell-based and plasma stability of α-helix mimetic **27**, β-hairpin **23**, proteomimetic **28**, and scrambled proteomimetic **29**. S19

Figure S17. Human bronchial epithelial cell permeability model. S20

2. Peptide characterization **21**

Table S1. Number, name, sequence, formula, exact calculated mass, observed mass, and purity of linear native hACE2-derived peptides. S21

Table S2. Number, name, sequence, formula, exact calculated mass, observed mass, and purity of *i, i + 4* and *i, i + 7* hACE2 α1-helix-derived staple peptides. S21

Table S3. Number, name, sequence, formula, exact calculated mass, observed mass, and purity of β-hairpin mimetics. S21

Table S4. Number, name, sequence, formula, exact calculated mass, observed mass, and purity of parent compound **27**... S21

Table S5. Number, name, sequence, formula, exact calculated mass, observed mass, and purity of proteomimetic **28**. S21

Table S6. Number, name, sequence, formula, exact calculated mass, observed mass, and purity of the scrambled proteomimetic used as negative control. S21

2.1 Supplementary UPLC-MS spectra S22

2.2 Supplementary NMR spectra S51

1. Supplementary figures

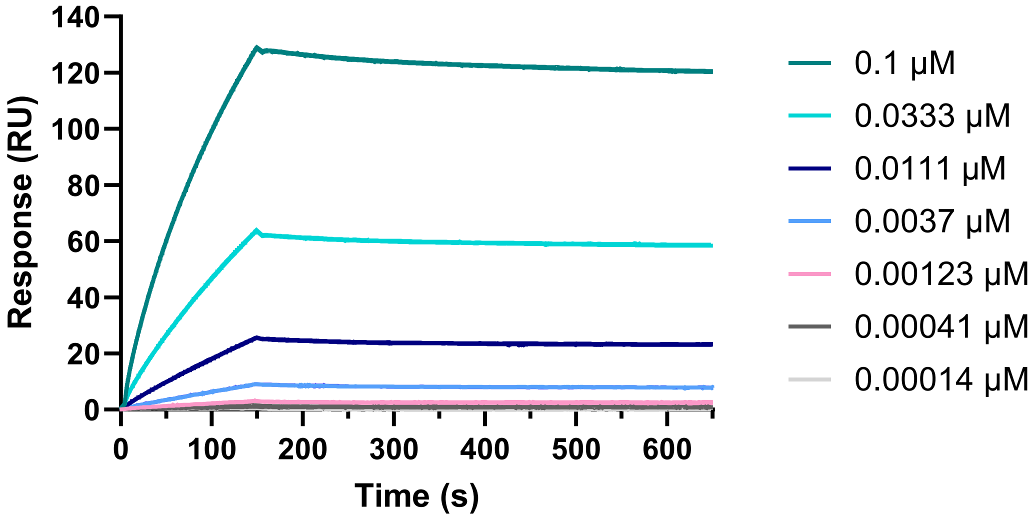

Figure S1. Surface plasmon resonance (SPR) sensorgram showing the binding kinetics for recombinant hACE2 with captured His-tagged SARS-CoV-2 S protein. Increasing concentrations of hACE2 were injected over the surface. Contact time and dissociation time were kept at 150 and 500 s, respectively. The highest response reached about 127.6 RUs at 0.1 µM, while the deviation of duplicate injections did not exceed 2 RUs.

A

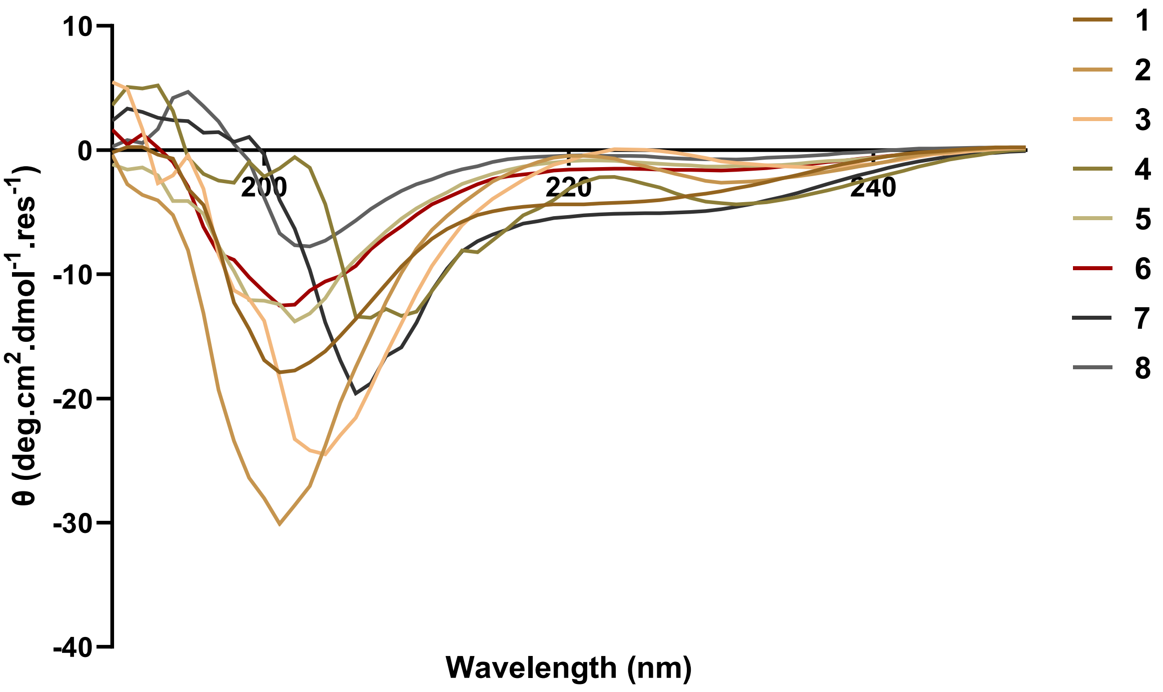

B

Cmpd	Sequence	Derived from	Helicity[a] (at 222 nm, %)
**1**	Ac-NH-STIEEQAKTFLDK-CO-NH$_2$	hACE2 (19-31)	17.5
**2**	Ac-NH-DKFNHED-CO-NH$_2$	hACE2 (30-38)	5.3
**3**	Ac-NH-HEAEDLFYQ-CO-NH$_2$	hACE2 (34-42)	4.7
**4**	Ac-NH-DKFNHEDLFYQ-CO-NH$_2$	hACE2 (30-42)	10.8
**5**	Ac-NH-QAKTFLDKFNHEAEDLFYQ-CO-NH$_2$	hACE2 (24-42)	5.3
**6**	Ac-NH-EQAKTFLDKFNHEAEDLFYQSS-CO-NH$_2$	hACE2 (23-44)	7.3
**7**	Ac-NH-IEEQAKTFLDKFNHEAEDLFYQS-CO-NH$_2$	hACE2 (21-43)	18.1
**8**	Ac-NH-EQAKTFLDKFNHEAEDLFYQS-CO-NH$_2$	hACE2 (23-43)	4.0

Figure S2. Helicity assessment of native hACE2 α1-helix-derived linear compounds. (**A**) Far-UV CD spectra of linear native hACE2-derived peptides measured at 100 µM in 10 mM sodium phosphate buffer at pH = 7.4 and 25 °C. (**B**) Table summarizing the linear peptide sequences derived from the native hACE2 helical motif and their corresponding estimated helicity values[a] (%). Natural amino acids are abbreviated using their standard one-letter codes.

[a]Helicity (at 222 nm, %) was calculated from far-UV CD spectral data, as described in the Materials and Methods section.

A

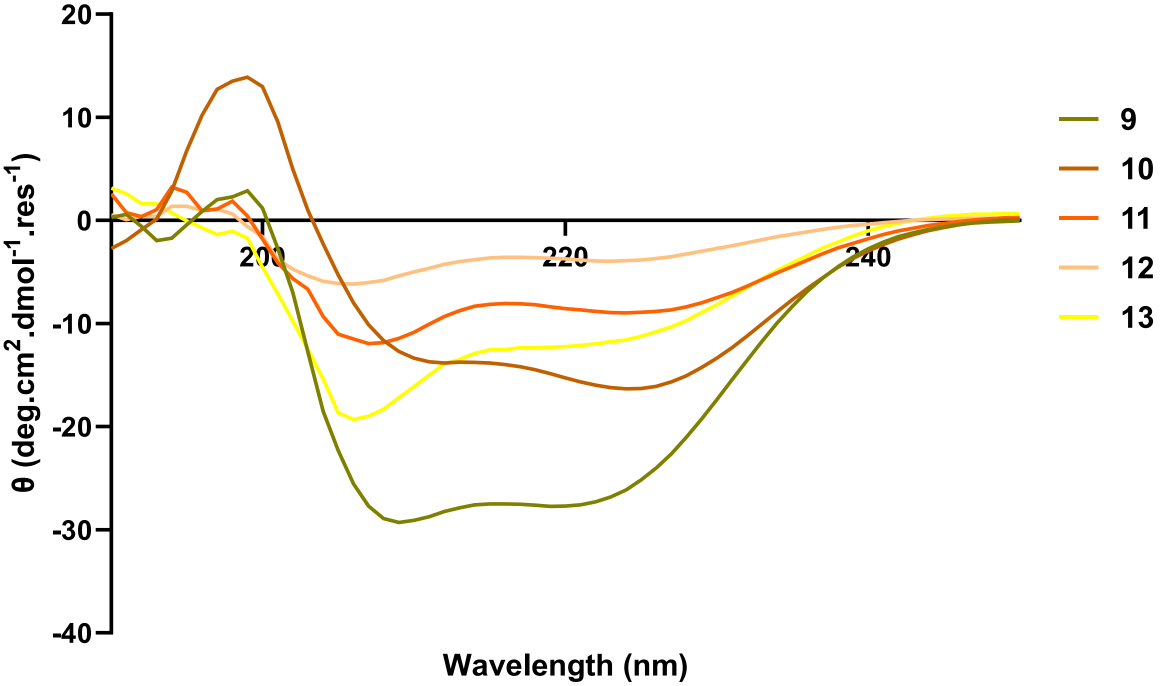

B

Cmpd	Sequence	Helicity[a] (at 222 nm, %)	Helix type[b] Regular helix (%)	Helix type[b] Distorted helix (%)	Other[b] (%)
**9**	Ac-NH-EQARTF[KDKFD]HE[KEDLD]YQSS-CO-NH_2	84.7	53.4	6.4	40.1
**10**	Ac-NH-EQARTF[S5-DKF-S5]HE[S5-EDL-S5]YQSS-CO-NH_2	50.0	19.9	12.9	67.1
**11**	Ac-NH-EQAKTFLDK[R8-NHEAED-S5]FYQSS-CO-NH_2	28.8	0.0	0.0	100
**12**	Ac-NH-EQARTFLDK[L-Orn(N_3)-NHEAED-L-Orn(N_3)]FYQSS-CO-NH_2	14.3	0.0	1.3	98.7
**13**	Ac-NH-EQARTFLDK[L-Orn(N_3)-NHEAED-L-Orn(N_3)]FYQSS-CO-NH_2	38.6	17.5	10.9	71.7

Figure S3. Helicity assessment of *i, i + 4* and *i, i + 7* hACE2 α1-helix-derived stapled peptides. (**A**) Far-UV CD spectra of stapled hACE2-derived peptides measured at 100 µM in 10 mM sodium phosphate buffer at pH = 7.4 and 25 °C. (**B**) Table summarizing stapled peptide sequences, calculated helicity values[a] (%), and secondary structure extrapolation[b], using the BestSel online analytic tool. Natural amino acids are abbreviated using their standard one-letter codes. S5 and R8 denote the non-proteinogenic amino acids α-(4-pentenyl)-L-Alanine and α-(7′-octenyl)-D-Alanine, respectively; L-Orn(N_3) denotes δ-azido-L-Ornithine.

[a]Helicity (at 222 nm, %) was calculated from far-UV CD spectral data, as described in the Materials and Methods section.

[b]Secondary structure assignments were extrapolated from far-UV CD spectral data using BestSel. The "other" classification category includes minor conformations, such as parallel structures and other disordered configurations.

A

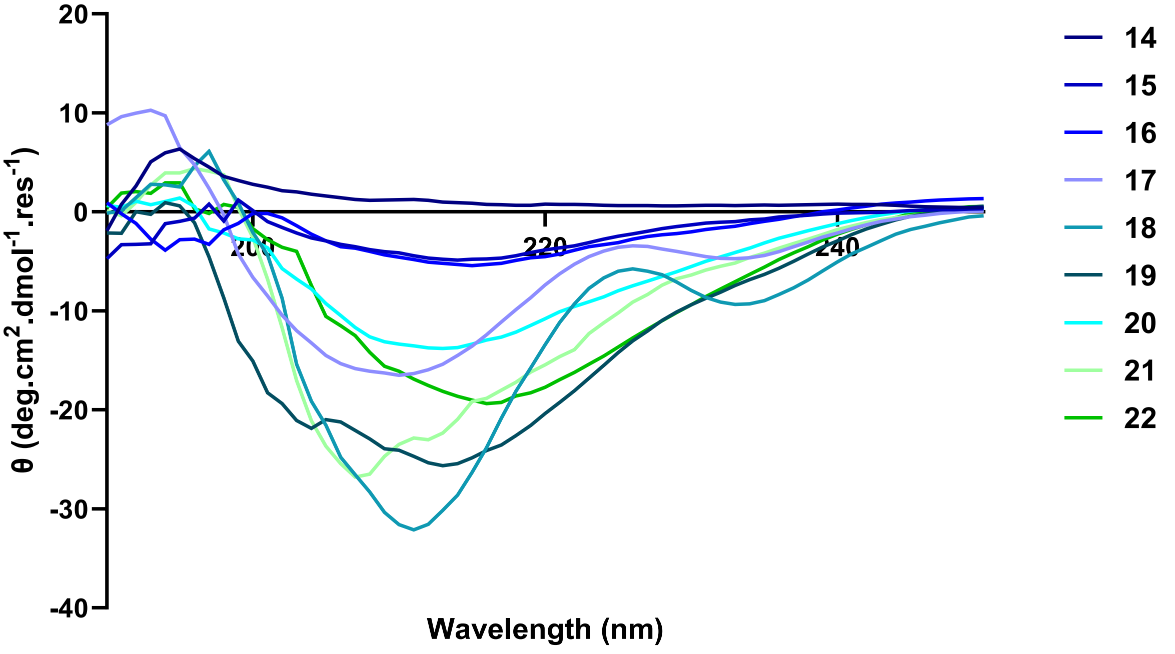

B

Cmpd	Sequence	Antiparallel β-sheet[a]			Turn[a]	Other[a]
		Left-twisted	Relaxed	Right-twisted		
**14**	[GDFRpPDLGK]	0.0	23.9	17.2	17.5	41.5
**15**	[KGDFpPWDLG]	0.0	16.1	16.2	13.9	53.8
**16**	[GDFRIpPWDLGK]	3.5	10.2	25.8	15.8	44.7
**17**	[KGDFRpPAWDLG]	0.0	0.0	11.8	8.0	76.6
**18**	[GDFRpPWDLGK]	0.0	0.0	29.5	0.0	70.5
**19**	[GDFRIpPAWDLGK]	1.3	0.0	21.6	1.2	61.7
**20**	[GDFRILpPTAWDLGK]	0.0	0.0	14.4	12.2	73.5
**21**	[GDFRILpPAWDLGK]	0.0	0.0	22.3	4.8	72.9
**22**	[GDFRIpPTAWDLGK]	0.0	0.0	17.0	9.1	74.0

Figure S4. β-hairpin mimetics of the hACE2 antiparallel β-sheet (Thr347-Leu359) with secondary structure assignments. (**A**) Far-UV CD spectra of β-hairpin mimetics recorded at 100 µM in 10 mM sodium phosphate buffer (pH 7.4) at 25 °C. (**B**) Table summarizing the β-hairpin peptide sequences and their corresponding secondary structure specifications[a]. Natural amino acids are abbreviated using their standard one-letter codes, while D-stereochemistry is indicated by lowercase notation; i.e., P denotes L-Proline and p denotes D-Proline.

[a]Secondary structure assignments were extrapolated from far-UV CD spectral data using BestSel. The "other" classification category includes minor conformations, such as helices, parallel structures, and other disordered configurations.

A

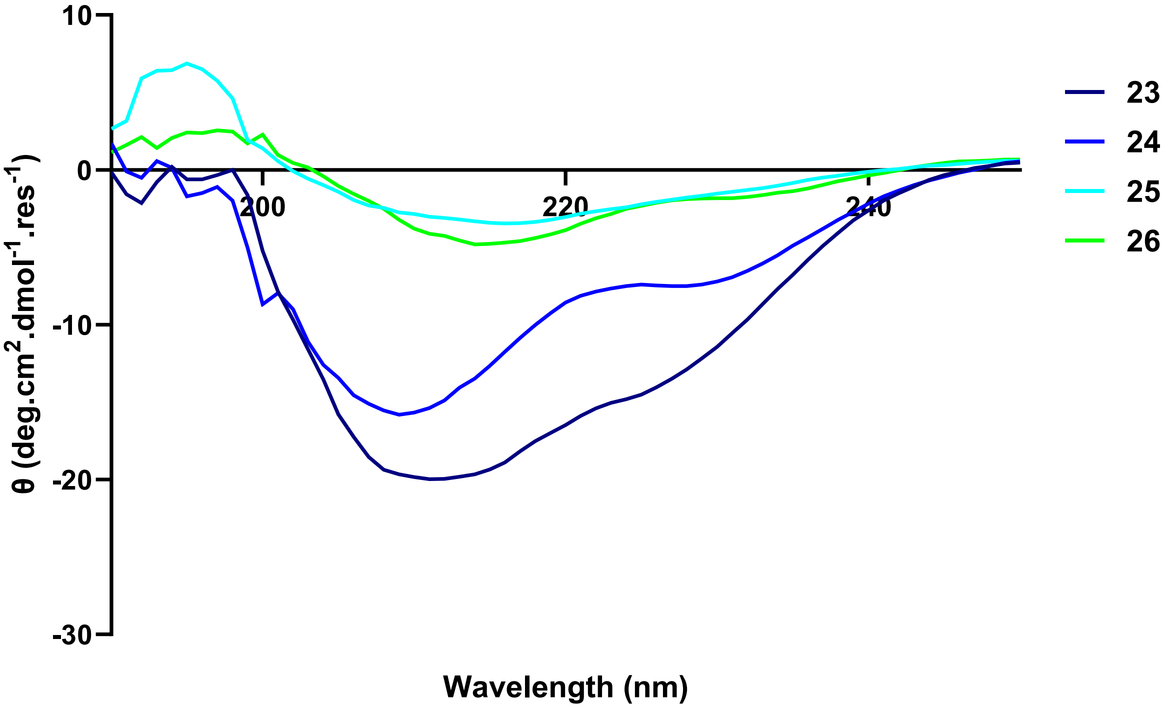

B

Cmpd	Sequence	Antiparallel β-sheet[a] Left-twisted	Relaxed	Right-twisted	Turn[a]	Other[a]
**23**	[GDFRpPD-L-Pra-GK]	0.0	0.0	18.5	8.4	73.1
**24**	[GDFRpPD-D-Pra-GK]	0.0	0.0	16.1	11.8	72
**25**	[GDFRIpPWD-L-Pra-GK]	4.7	14.9	16.6	16.0	47.7
**26**	[GDFRIpPWD-D-Pra-GK]	6.4	18.3	16.3	11.7	47.3

Figure S5. β-hairpin mimetics of the hACE2 antiparallel β-sheet (Thr349-Ile358) with secondary structure assignments. (**A**) Far-UV CD spectra of β-hairpin mimetics recorded at 100 µM in 10 mM sodium phosphate buffer (pH 7.4) at 25 °C. (**B**) Table summarizing the β-hairpin peptide sequences and their corresponding secondary structure specifications[a]. The three-letter abbreviations L-Pra and D-Pra stand for L-Propargylglycine and D-Propargylglycine, respectively. Natural amino acids are designated using their standard one-letter codes, while D-stereochemistry is indicated by lowercase notation; i.e., P denotes L-Proline and p denotes D-Proline.

[a]Secondary structure assignments were extrapolated from far-UV CD spectral data using BestSel. The "other" classification category includes minor conformations, such as helices, parallel structures, and other disordered configurations.

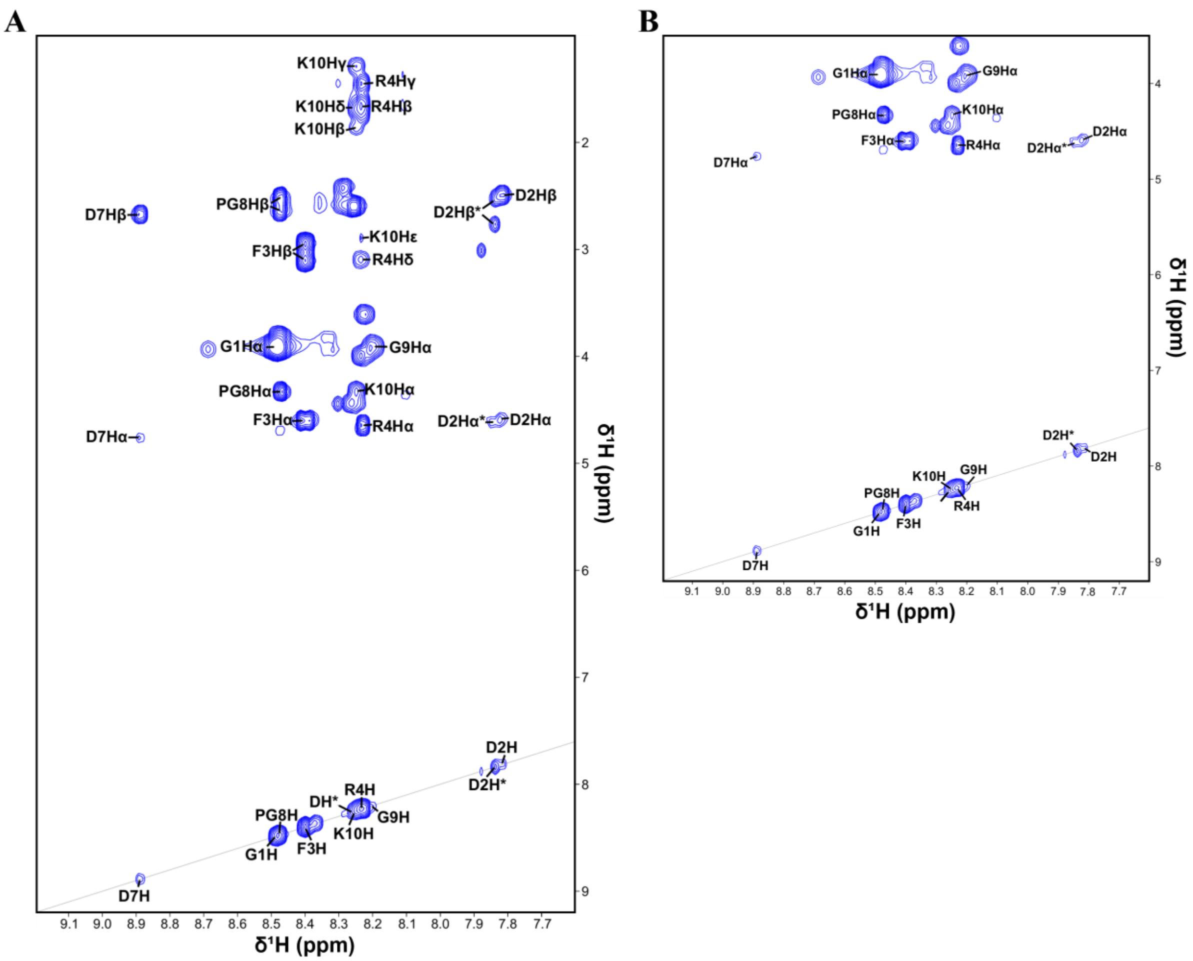

Figure S6. Total correlation spectroscopy (TOCSY) nuclear magnetic resonance (NMR) spectral regions recorded for β-hairpin mimetic 23. (**A**) Fingerprint region of 2D TOCSY spectrum displaying intra-residue scalar-coupled correlations within the side-chain spin systems of the peptide **23** residues, i.e., residues annotated Gly^1, Asp^2, Phe^3, Arg^4, D-Pro^5, L-Pro^6, Asp^7, L-Pra^8, Gly^9, Lys^{10}. The Asp residue marked with an asterisk (*) corresponds to a minor conformational population. (**B**) Amide (HN) region highlighting HN-Hα correlations used for residue-specific spin-system identification and assignment.

A

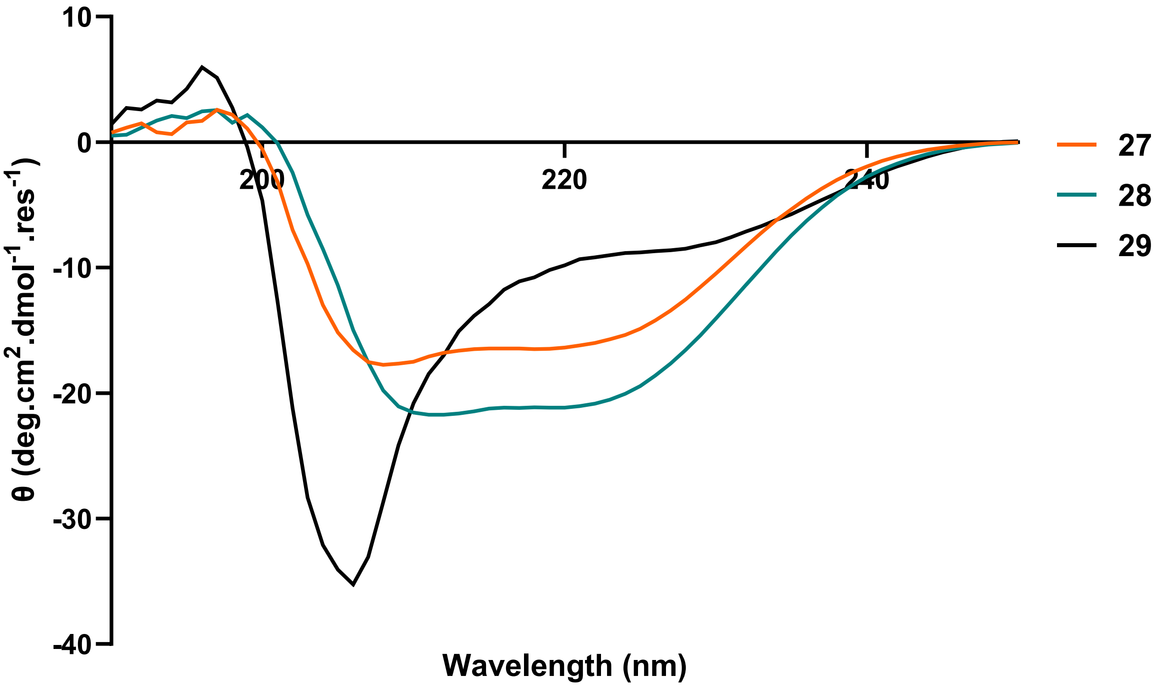

B

Cmpd	Helicity[a] (at 222 nm, %)	Helix type[b] Regular helix (%)	Helix type[b] Distorted helix (%)	Antiparallel β-sheet[b] Left-twisted (%)	Antiparallel β-sheet[b] Relaxed (%)	Antiparallel β-sheet[b] Right-twisted (%)	Turn[b] (%)	Other[b] (%)
**27**	50.8	81.2	0	-	-	-	-	18.8
**28**	61.9	35.3	8.2	4.4	0	21.0	8.9	30.4
**29**	10.8	-	-	-	-	-	-	-

Figure S7. Helicity assessment (%) of the proteomimetic parent compound 27 (α-helix mimetic), the proteomimetic 28, and the scrambled sequence 29 of proteomimetic 28. (**A**) Far-UV CD spectra of **27**, **28**, and **29** measured at 100 µM in 10 mM sodium phosphate buffer at pH = 7.4 and 25 °C. (**B**) Table summarizing estimated helicity values[a] (%), and secondary structure extrapolation[b], determined using the BestSel online analytic tool.

[a]Helicity (at 222 nm, %) was calculated from far-UV CD spectral data, as described in the Materials and Methods section.

[b]Secondary structure assignments were extrapolated from far-UV CD spectral data using BestSel. The "other" classification category includes minor conformations, such as parallel structures and other disordered configurations.

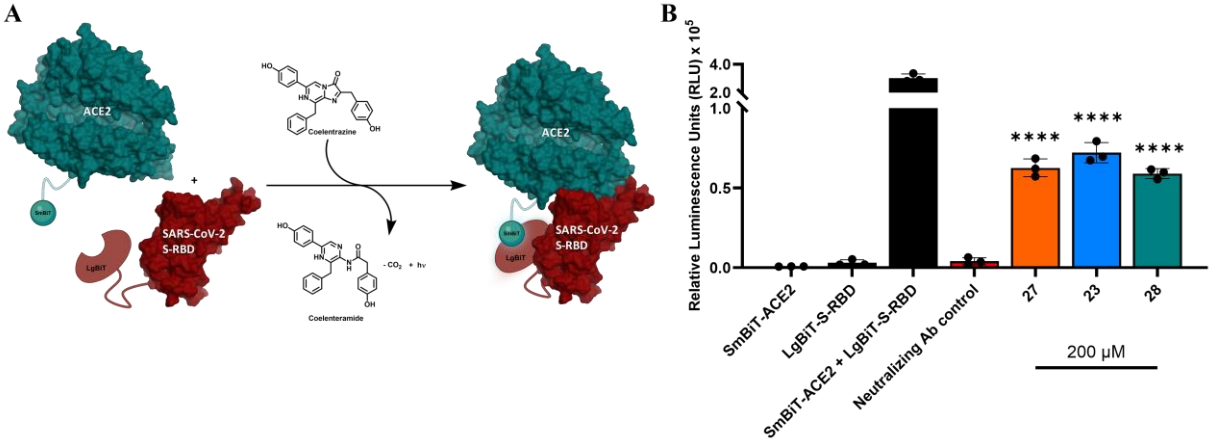

Figure S8. SARS-CoV-2 S-RBD/hACE2 NanoBiT-based biosensor assay for comparative analysis of PPI disruption capacities. (**A**) Schematic representation of the NanoBiT-based biosensor assay. The Nanoluciferase binary technology (NanoBiT) is a structural complementation reporter system composed of a Large BiT (LgBiT) and a Small BiT (SmBiT), each fused to the proteins of interest, the SARS-CoV-2 S-RBD domain and the humanACE2 ectodomain, respectively. When the two fusion proteins interact, the LgBiT and SmBiT fragments reconstitute an active NanoLuc enzyme that catalyzes the oxidation of coelenterazine, resulting in the emission of a bright luminescent signal due to photon release. Structural model based on PDB ID: 6M0J. (**B**) NanoBiT-based biosensor assay performed with compounds **27**, **23**, and **28**. The SmBiT-ACE2 + LgBiT-RBD positive control was treated with DMSO at a concentration equivalent to that used for 200 µM compound treatments. Bars represent mean values from three independent experiments, performed in triplicate. Individual experimental means are shown as circles. Error bars represent standard deviations. Relative Luminescence units (RLU) were compared to the SmBiT-ACE2 + LgBiT-RBD positive control using one-way analysis of variance (ANOVA) followed by Tukey's multiple comparison correction. For all analyses, ****$p < 0.0001$.

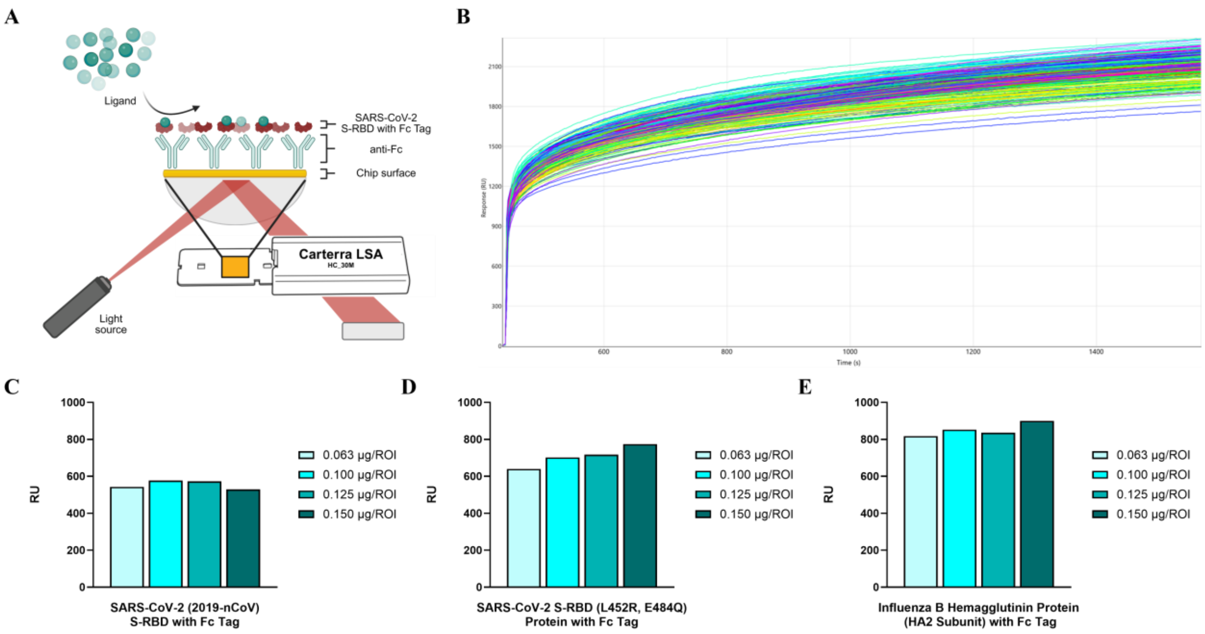

Figure S9. SPR-based concentration-response assay performed on the Carterra LSA. (**A**) Schematic representation of SPR-based assay. Fc-tagged proteins were immobilized on a SPR HC_30M chip via an anti-Fc antibody, to evaluate the binding capacity of ligands to the SARS-CoV-2 S-RBD. (**B**) Assessment of anti-Fc antibody coupling efficiency for SPR K_D determination. Non-covalent capture of SARS-CoV-2 (2019-nCoV) S-RBD (**C**), SARS-CoV-2 S-RBD (L452R, E484Q) (**D**), and Influenza B Hemagglutinin (HA2 Subunit) (**E**) Fc tagged proteins to the anti-Fc lawn to establish optimal Fc-tag protein capture concentrations.

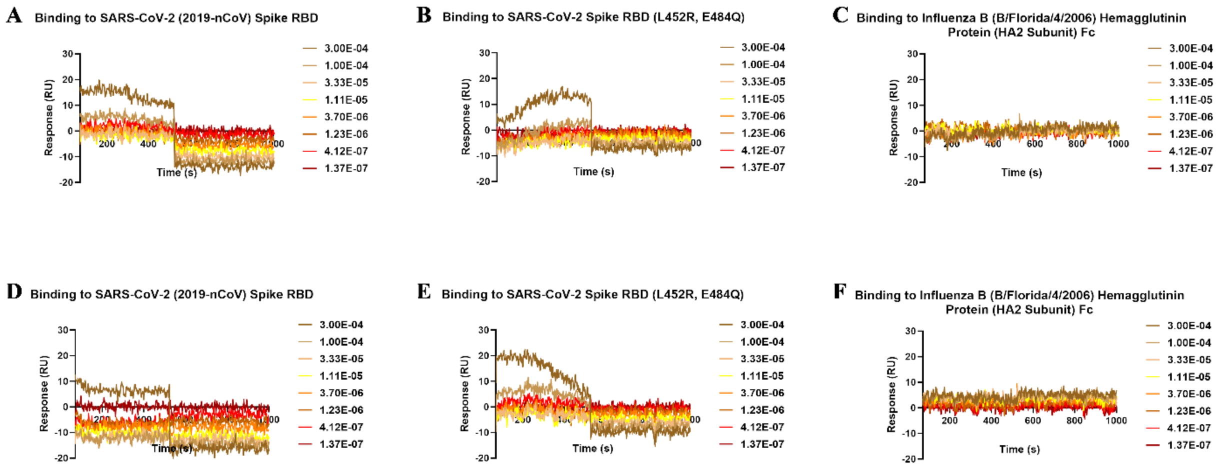

Figure S10. SPR sensorgrams for compound 27. Increasing concentrations of **27** were injected over sensor chip surfaces immobilized with SARS-CoV-2 (2019-nCoV) S-RBD-Fc (**A** and **D**), SARS-CoV-2 S-RBD-Fc (L452R, E484Q) mutant (**B** and **E**), and Influenza B (B/Florida/4/2006) Hemagglutinin Protein (HA2 Subunit)-Fc protein (**C** and **F**). Two representative sensorgrams from independent experiments are shown for each immobilized surface.

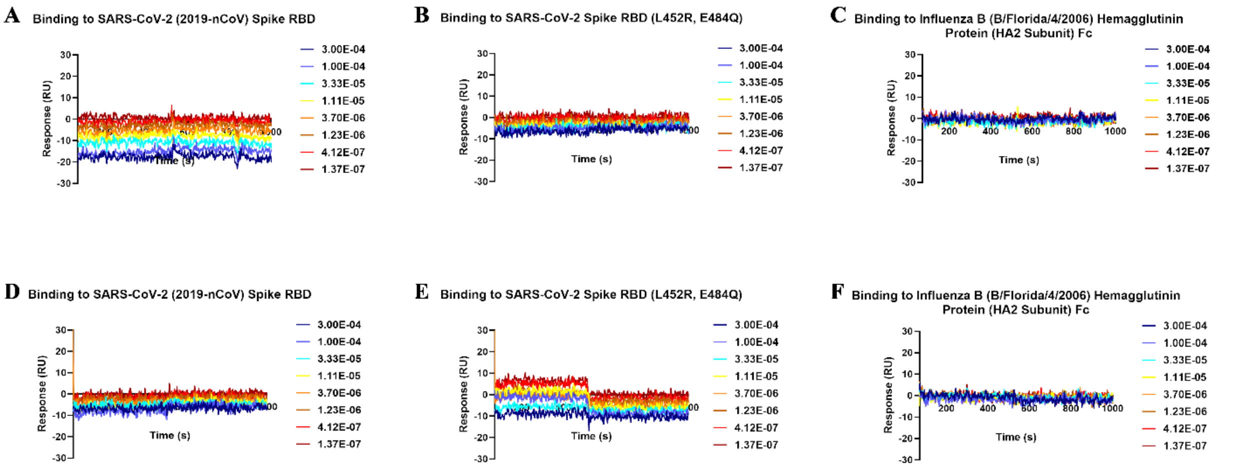

Figure S11. SPR sensorgrams for compound 23. Increasing concentrations of **23** were injected over sensor chip surfaces immobilized with SARS-CoV-2 (2019-nCoV) S-RBD-Fc (**A** and **D**), SARS-CoV-2 S-RBD-Fc (L452R, E484Q) mutant (**B** and **E**), and Influenza B (B/Florida/4/2006) Hemagglutinin Protein (HA2 Subunit)-Fc protein (**C** and **F**). Two representative sensorgrams from independent experiments are shown for each immobilized surface.

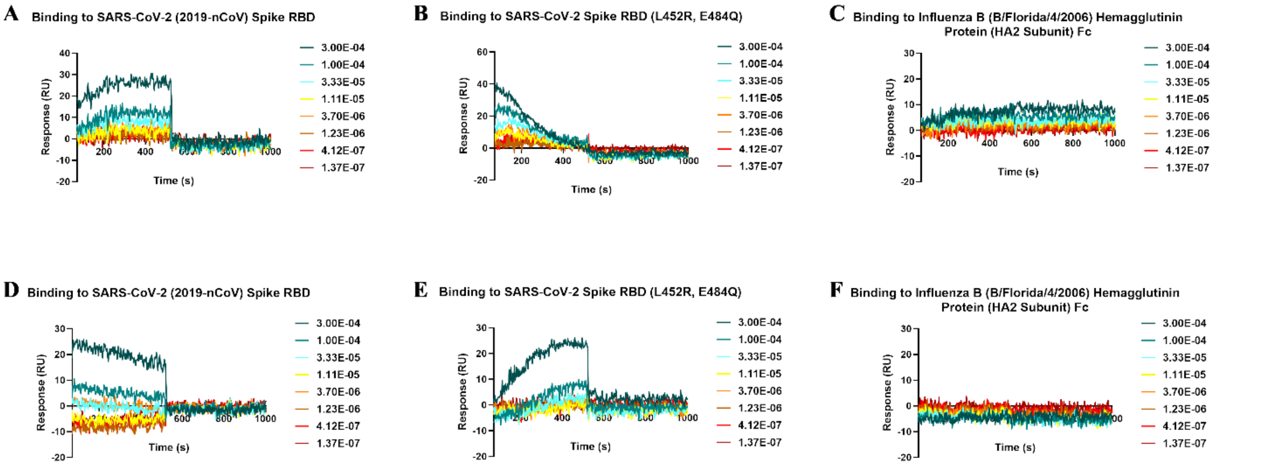

Figure S12. SPR sensorgrams for compound 28. Increasing concentrations of **28** were injected over sensor chip surfaces immobilized SARS-CoV-2 (2019-nCoV) S-RBD-Fc (**A** and **D**), SARS-CoV-2 S-RBD-Fc (L452R, E484Q) mutant (**B** and **E**) and Influenza B (B/Florida/4/2006) Hemagglutinin Protein (HA2 Subunit)-Fc protein (**C** and **F**). Two representative sensorgrams from independent experiments are shown for each immobilized surface.

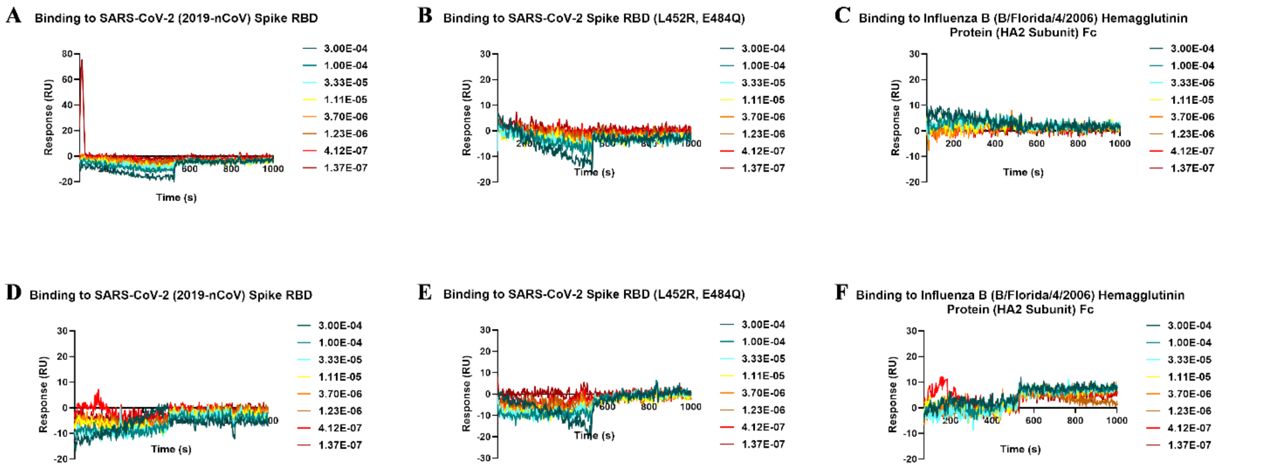

Figure S13. SPR sensorgrams for 29. Increasing concentrations of **29** were injected over sensor chip surfaces immobilized SARS-CoV-2 (2019-nCoV) S-RBD-Fc (**A** and **D**), SARS-CoV-2 S-RBD-Fc (L452R, E484Q) mutant (**B** and **E**) and Influenza B (B/Florida/4/2006) Hemagglutinin Protein (HA2 Subunit)-Fc protein (**C** and **F**). Two representative sensorgrams from independent experiments are shown for each immobilized surface.

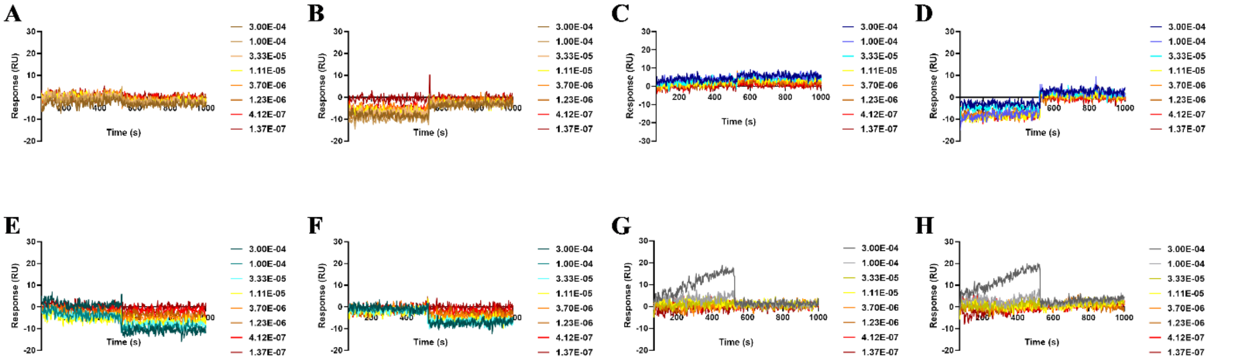

Figure S14. SPR sensorgrams generated upon buffer chip conditioning. Increasing concentrations of compounds **27** (**A** and **B**), **23** (**C** and **D**), **28** (**E** and **F**), and **29** (**G** and **H**) were injected over a protein-free SPR sensor chip. Two representative sensorgrams from independent experiments are shown for each immobilized surface.

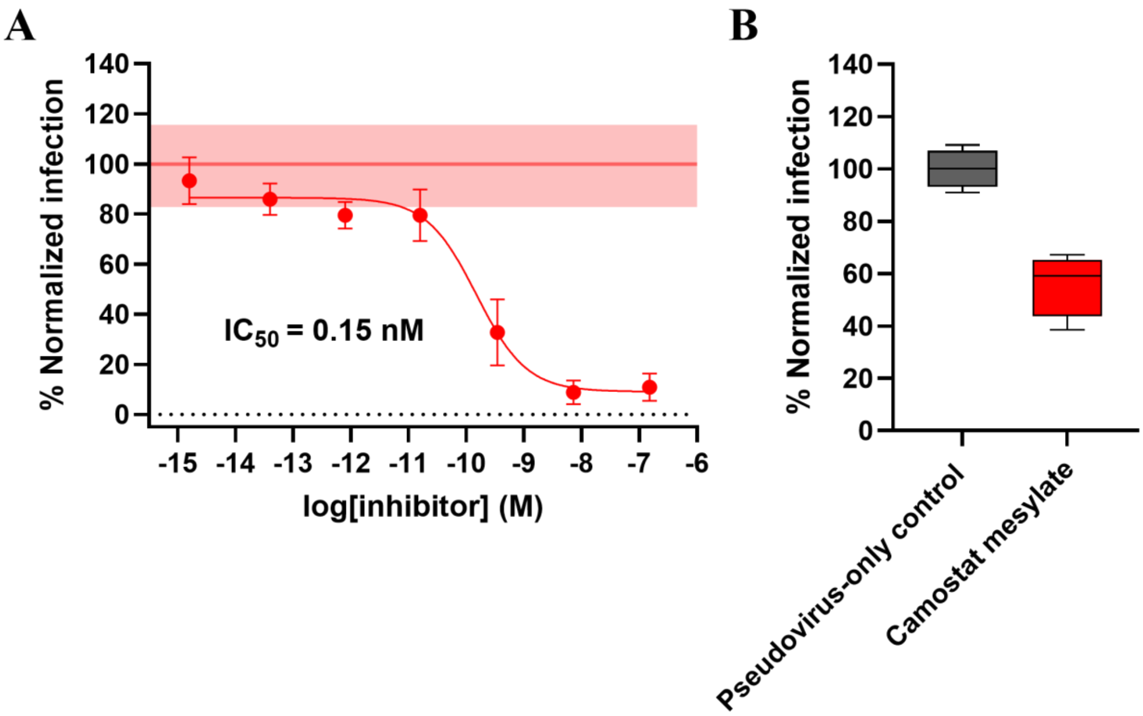

Figure S15. Neutralization potency of control agents in the pseudovirus-based neutralization assay. (**A**) Representative dose-response curve for the monoclonal anti-SARS-related coronavirus 2 S-RBD-mFc fusion protein. A representative dose-response curve was generated using increasing concentrations of the monoclonal anti-SARS-related coronavirus 2 S-RBD-mFc fusion protein (Sino Biological Catalog No. 40592-MM57). Experiment was replicated in triplicate (n = 2). (**B**) Camostat mesylate-mediated reduction of infection at 8 μM. Experiment was replicated in duplicate (n = 2). Fluorescence data (RFUs) were normalized to the infection-free control (Dulbecco's Eagle Medium; DMEM) set as 0% infection; and to the pseudovirus-only control, representing 100% infection. Data analysis and curve fitting were performed using GraphPad Prism software (version 9.3.1). Mean values and standard error of the mean (SEM) are shown, derived from two independent experiments (n = 2), each performed in duplicate or triplicate.

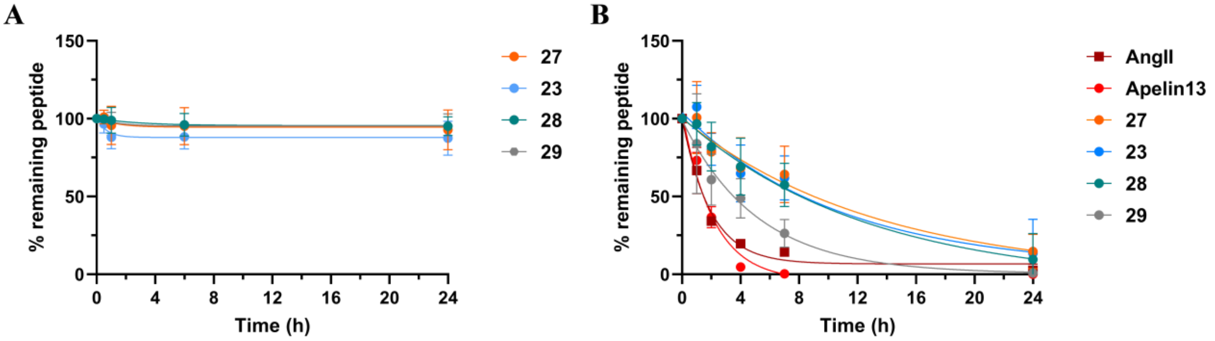

Figure S16. Calu-3 cell-based and plasma stability of α-helix mimetic 27, β-hairpin 23, proteomimetic 28, and scrambled proteomimetic 29. (**A**) Calu-3 cell-based stability of compounds **27**, **23**, **28**, and **29**, represented as the percentage of residual peptide over time. Peptide degradation was monitored using UPLC-MS. Data are plotted as means ± SEM from three independent experiments (n = 3), each performed in triplicate. (**B**) Proteolytic stability of **27**, **23**, **28**, and **29** in rat plasma, measured as the percentage of residual peptide over 24 h of incubation at 37 °C. Peptide degradation was monitored using UPLC-MS. Angiotensin II (AngII) and Apelin13 were included as reference controls due to their known short *in vitro* plasma half-lives ($t_{1/2}$). Data represent means ± SEM from three independent experiments (n = 3), each performed in triplicate.

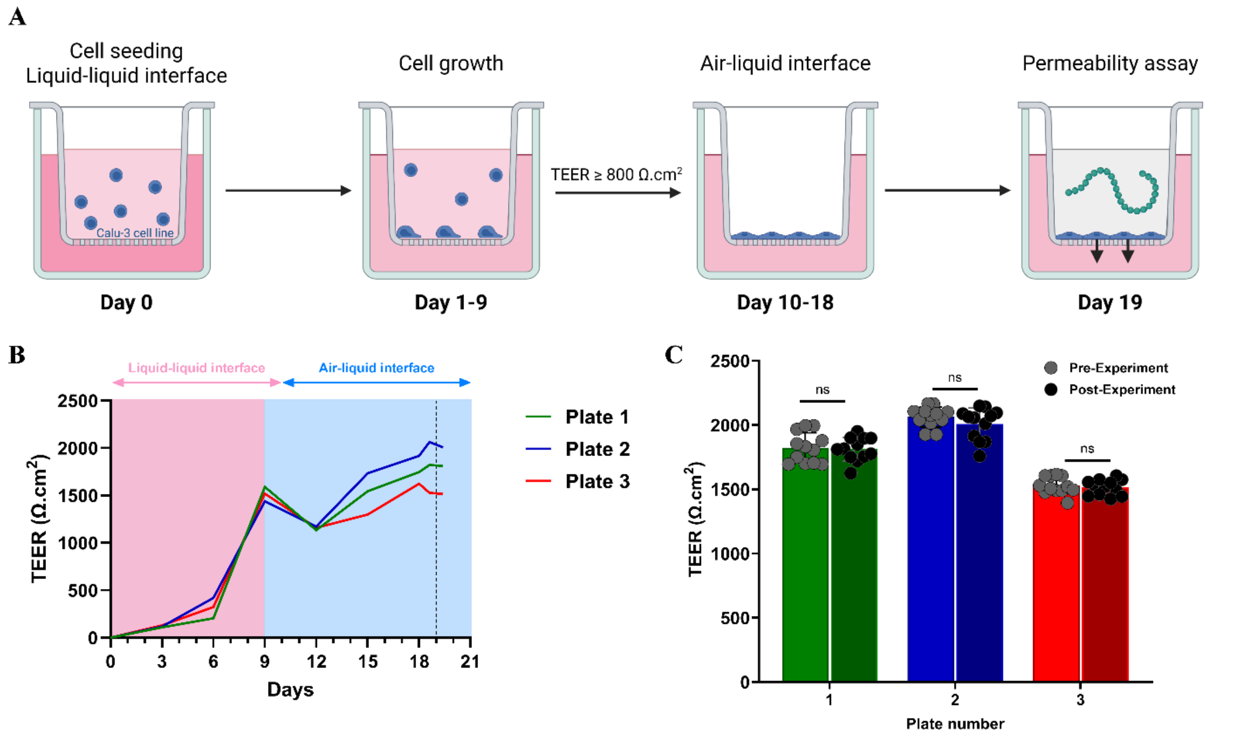

Figure S17. Human bronchial epithelial cell permeability model. (**A**) Schematic representation of the experimental protocol for the permeability assay using the Calu-3 cell line. (**B**) Time-dependent evolution of transepithelial electrical resistance (TEER) in Calu-3 cells cultured under liquid-liquid and air-liquid interface conditions over 19 days. Fitted lines from three separate independent experiments (Plates 1-3) are shown and represent the mean TEER value from 12-wells of a Costar® transwell plate, measured in duplicate. (**C**) Comparison of TEER values pre- and post-permeability assay. Columns represent the average difference in TEER across 12-wells of a Costar® transwell plate, measured in duplicate. Error bars represent standard deviation (SD).

2. Peptide characterization

N°	Name	Sequence	Formula	Mass (Da)			Purity (%)
				Calculated	UPLC-MS	HRMS	
1	SF03-13	Ac-NH-STIEEQAKTFLDK-CO-NH_2	C68H111N17O24	1550.73	776.06	775.9080	96
2	SF03-01	Ac-NH-DKFNHED-CO-NH_2	C48H68N14O19	1145.15	1045.90	573.2477	99
3	SF03-02	Ac-NH-HEAEDLFYQ-CO-NH_2	C54H73N13O18	1192.25	1193.19	596.7688	100
4	SF03-03	Ac-NH-DKFNHEDLFYQ-CO-NH_2	C77H105N19O25	1696.80	849.43	848.8860	95
5	SF03-04	Ac-NH-QAKTFLDKFNHEAEDLFYQ-CO-NH_2	C110H157N27O33	2385.62	1193.83	795.7219	95
6	SF03-12	Ac-NH-EQAKTFLDKFNHEAEDLFYQSS-CO-NH_2	C121H174N30O40	2688.89	897.20	896.7556	95
7	SF03-33	Ac-NH-IEEQAKTFLDKFNHEAEDLFYQS-CO-NH_2	C129H187N31O42	2844.09	948.79	948.4555	95
8	SF03-35	Ac-NH-EQAKTFLDKFNHEAEDLFYQS-CO-NH_2	C118H169N29O38	2601.82	868.00	868.4098	98

Table S1. Number, name, sequence, formula, exact calculated mass, observed mass, and purity of linear native hACE2-derived peptides. Natural amino acids are abbreviated using their standard one-letter codes.

N°	Name	Sequence	Formula	Mass (Da)			Purity (%)
				Calculated	UPLC-MS	HRMS	
9	SF03-37	Ac-NH-EQARTF[KDKFD]HE[KEDLD]YQSS-CO-NH_2	C119H173N31O41	2693.87	899.0	898.4203	100
10	SF03-34	Ac-NH- EQARTF[S5-DKF-S5]HE[S5-EDL-S5]YQSS-CO-NH_2	C123H182N30O40	2720.98	907.44	907.4443	95
11	SF03-38	Ac-NH-EQAKTFLDK[R8-NHEAED-S5]FYQSS-CO-NH_2	C123H182N32O40	2748.99	917.28	916.7797	95
12	SF03-68	Ac-NH-EQARTFLDK[L-Orn(N_3)-NHEAED- L-Orn(N_3)]FYQSS-CO-NH_2	C126H176N38O40	2863.02	954.77	954.7722	100
13	SF03-52	Ac-NH-EQARTFLDK[L-Orn(N_3)-NHEAED- L-Orn(N_3)]FYQSS-CO-NH_2	C122H176N38O40	2814.98	939.23	704.3273	96

Table S2. Number, name, sequence, formula, exact calculated mass, observed mass, and purity of *i, i + 4* and *i, i + 7* hACE2 α1-helix-derived staple peptides. Natural amino acids are abbreviated using their standard one-letter codes. S5 and R8 denote the non-proteinogenic amino acids α-(4-pentenyl)-L-Alanine and α-(7′-octenyl)-D-Alanine, respectively; L-Orn(N_3) denotes δ-azido-L-Ornithine.

N°	Name	Sequence	Formula	Mass (Da)			Purity (%)
				Calculated	UPLC-MS	HRMS	
14	SF04-01	[GDFRpPDLGK]	C49H74N14O14	1083.22	1084.12	542.2838	96
15	SF04-02	[KGDFpPWDLG]	C54H72N12O14	1113.24	1114.15	557.2726	97
16	SF04-03	[GDFRIpPWDLGK]	C66H95N17O16	1382.59	1383.48	691.8653	100
17	SF04-04	[KGDFRpPAWDLG]	C63H89N17O16	1340.51	1341.44	670.8424	100
18	SF04-07	[GDFRpPWDLGK]	C60H84N16O15	1269.43	635.75	635.3226	100
19	SF04-08	[GDFRIpPAWDLGK]	C69H100N18O17	1453.67	727.78	727.3836	97
20	SF04-09	[GDFRILpPTAWDLGK]	C79H118N20O20	1667.93	834.91	834.4502	97
21	SF04-10	[GDFRILpPAWDLGK]	C75H111N19O18	1566.83	784.36	783.9242	100
22	SF04-11	[GDFRIpPTAWDLGK]	C73H107N19O19	1554.77	778.10	777.9057	95
23	SF03-45	[GDFRpPD-L-Pra-GK]	C48H68N14O14	1065.16	1065.68	533.2591	96
24	SF04-05	[GDFRpPD-D-Pra-GK]	C48H68N14O14	1065.16	1066.33	533.2613	96
25	SF04-06	[GDFRIpPWD-L-Pra-GK]	C65H89N17O16	1364.53	683.07	682.8408	95
26	SF04-12	[GDFRIpPWD-D-Pra-GK]	C65H89N17O16	1364.53	682.98	682.8404	98

Table S3. Number, name, sequence, formula, exact calculated mass, observed mass, and purity of β-hairpin mimetics. Natural amino acids are abbreviated using their standard one-letter codes, while D-stereochemistry is indicated by lowercase notation; i.e., P denotes L-Proline and p denotes D-Proline. The three-letter abbreviations L-Pra and D-Pra stand for L-Propargylglycine and D-Propargylglycine, respectively.

N°	Name	Sequence	Formula	Mass (Da)			Purity (%)
				Calculated	UPLC-MS	HRMS	
27	SF03-46	Ac-NH-EQAKTF[KDKFD]HE[KEDLD]YQS-L-Dap(N_3)-CO-NH_2	C119H172N34O40	2718.88	907.41	906.7582	95

Table S4. Number, name, sequence, formula, exact calculated mass, observed mass, and purity of parent compound 27. Natural amino acids are abbreviated using their standard one-letter codes. L-Dap(N_3) denotes β-azido-L-Alanine.

N°	Name	Formula	Mass (Da)			Purity (%)
			Calculated	UPLC-MS	HRMS	
28	SF03-47 (SF03-46 + SF03-45)	C167H240N48O54	3784.04	1892.31	757.3544	97

Table S5. Number, name, sequence, formula, exact calculated mass, observed mass, and purity of proteomimetic 28.

N°	Name	Sequence	Formula	Mass (Da)			Purity (%)
				Calculated	UPLC-MS	HRMS	
29	SF03-47 Scrambled	Ac-NH-KY-L-Dap(N_3)-DEKSEDAHPGDEDRF-L-Pra-KELQFKKF-CO-NH_2	C167H244N48O56	3820.07	637.67	764.5633	98

Table S6. Number, name, sequence, formula, exact calculated mass, observed mass, and purity of the scrambled proteomimetic used as negative control. Natural amino acids are abbreviated using their standard one-letter codes The three-letter abbreviations L-Dap(N_3) and L-Pra stand for β-azido-L-Alanine and L-Propargylglycine, respectively.

2.1 Supplementary UPLC-MS spectra

SF03-13: UPLC-MS

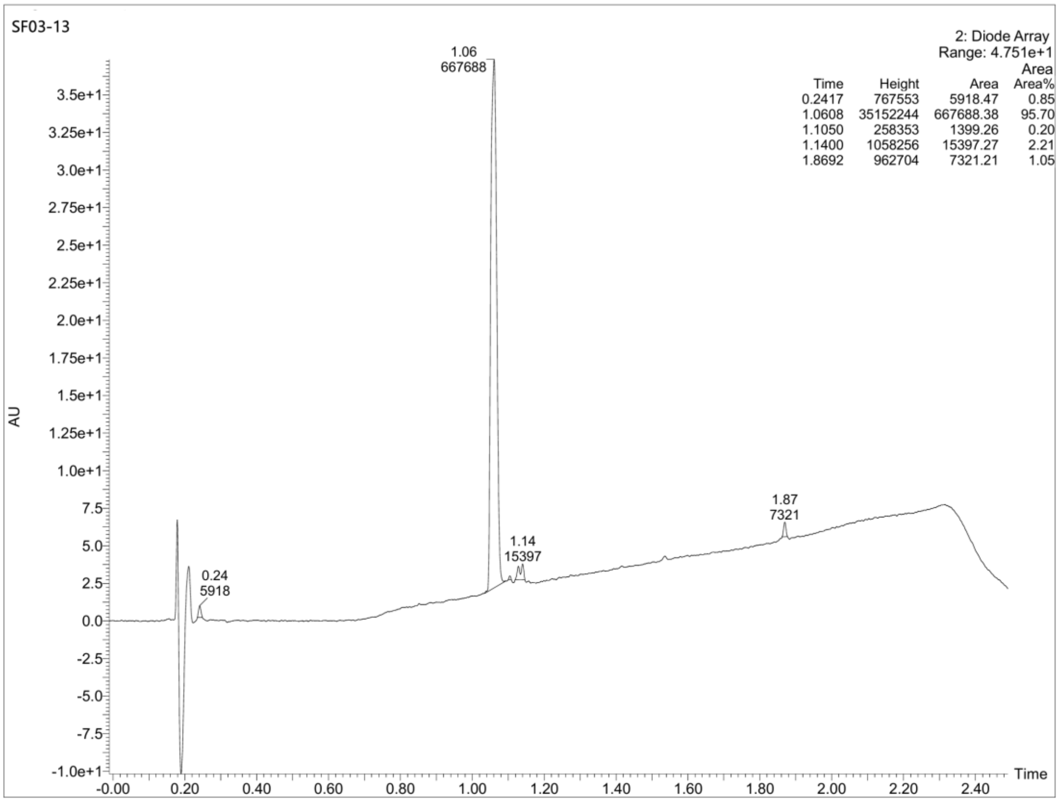

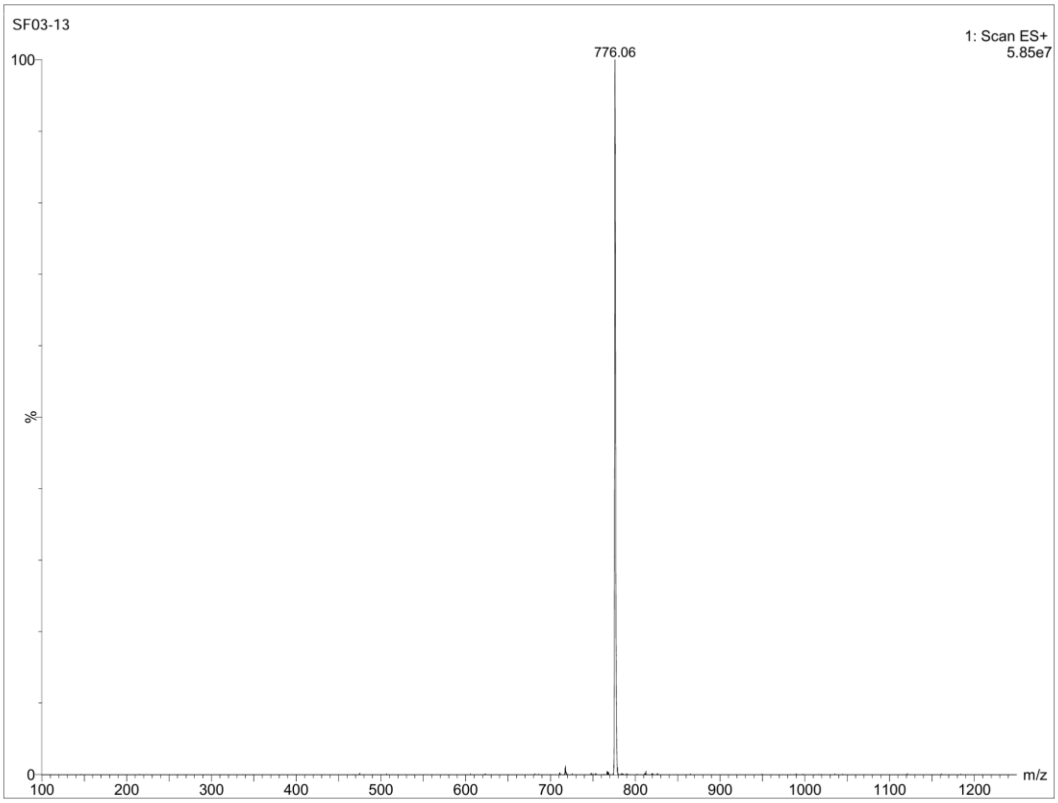

SF03-01: UPLC-MS

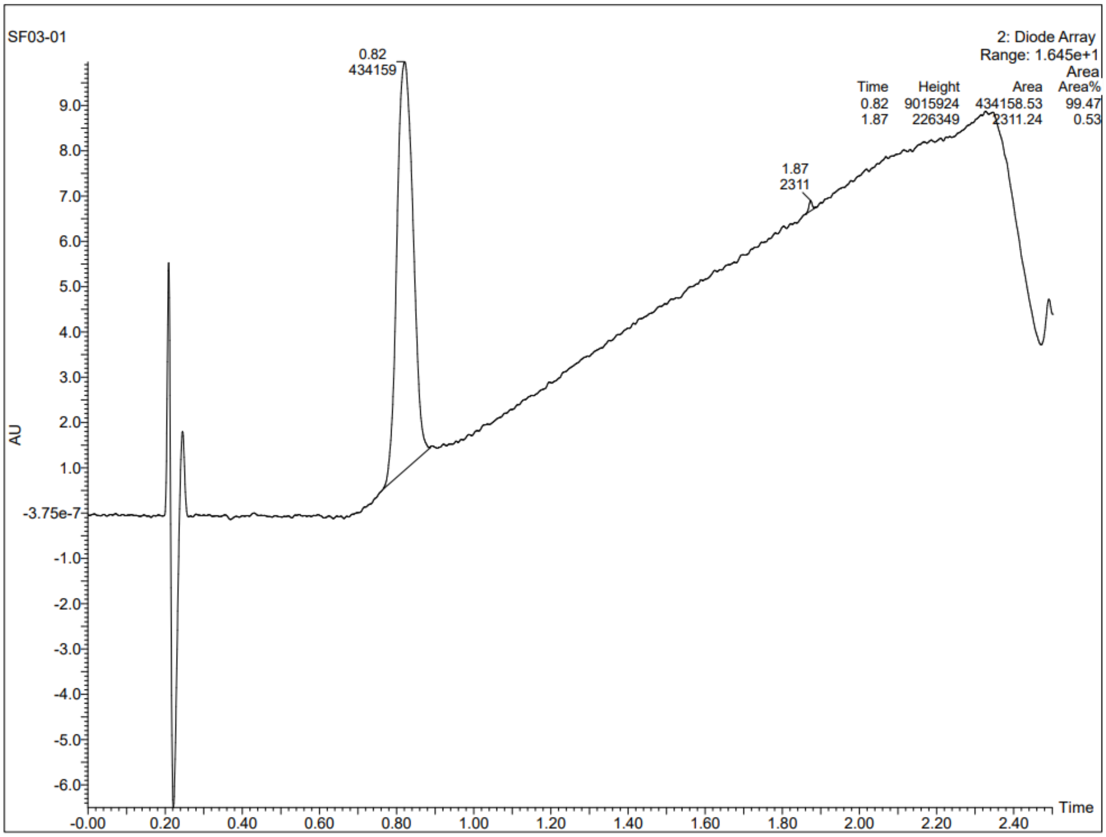

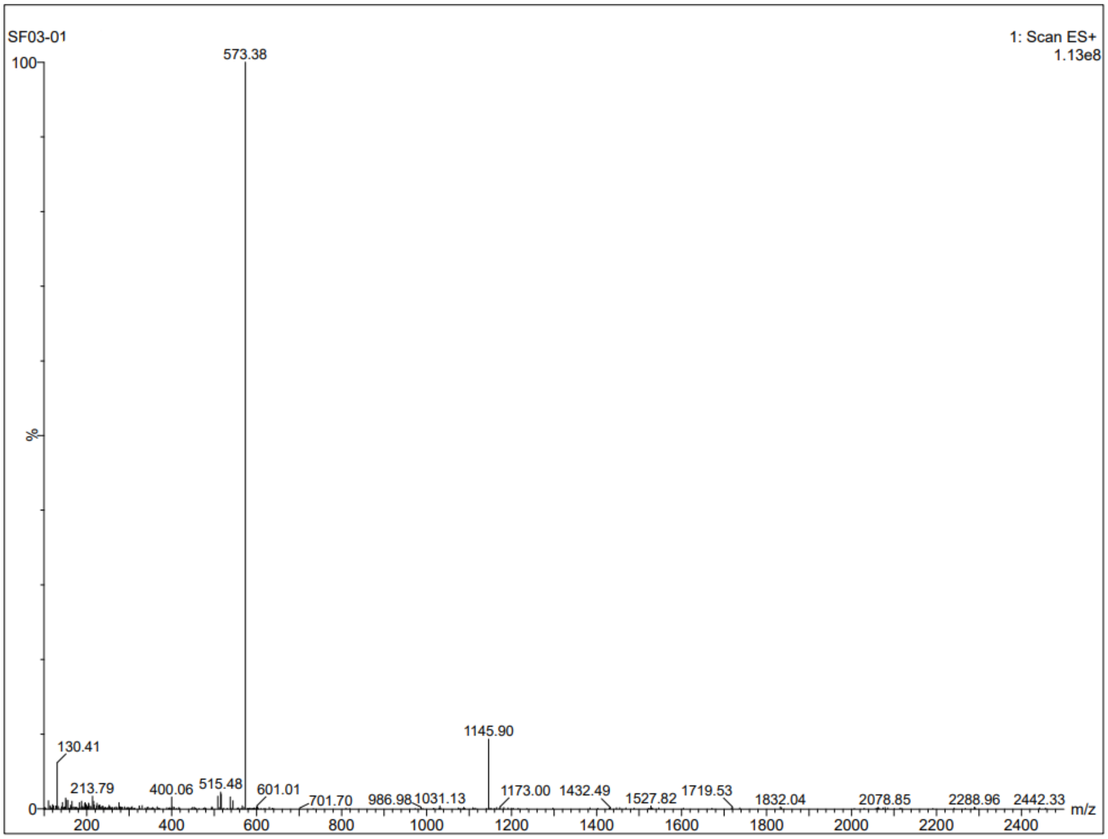

SF03-02: UPLC-MS

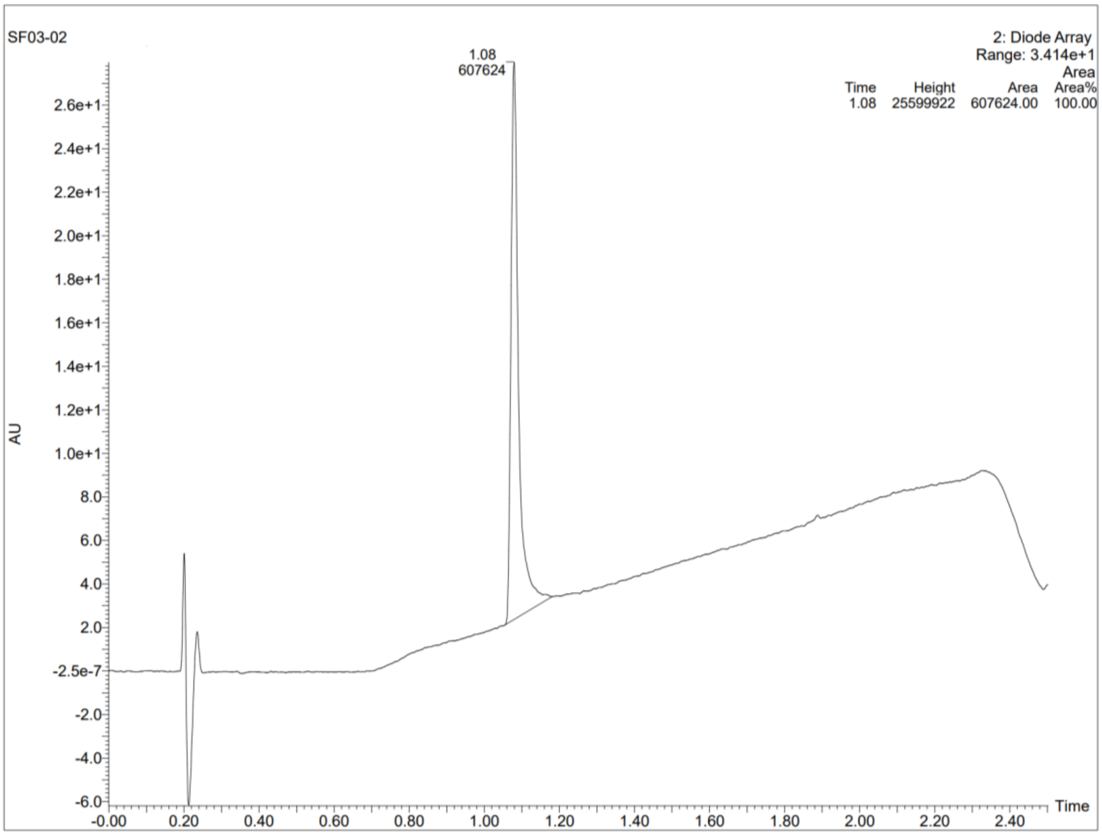

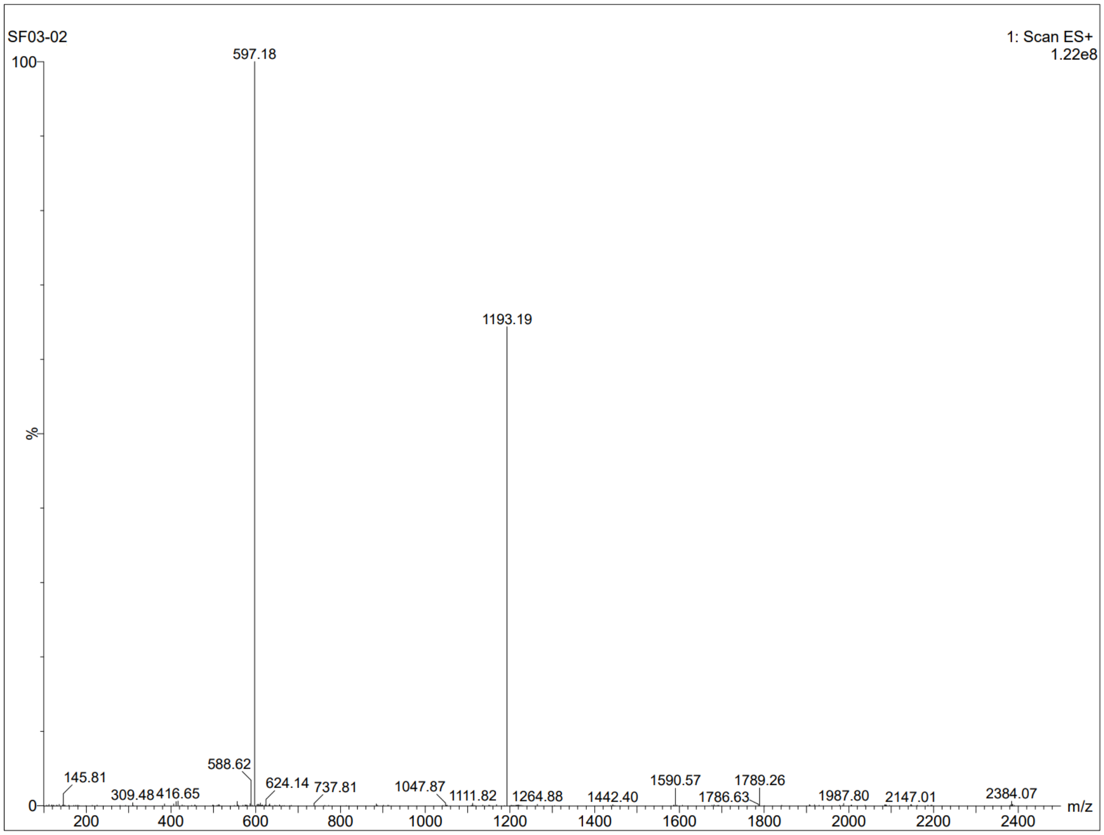

SF03-03: UPLC-MS

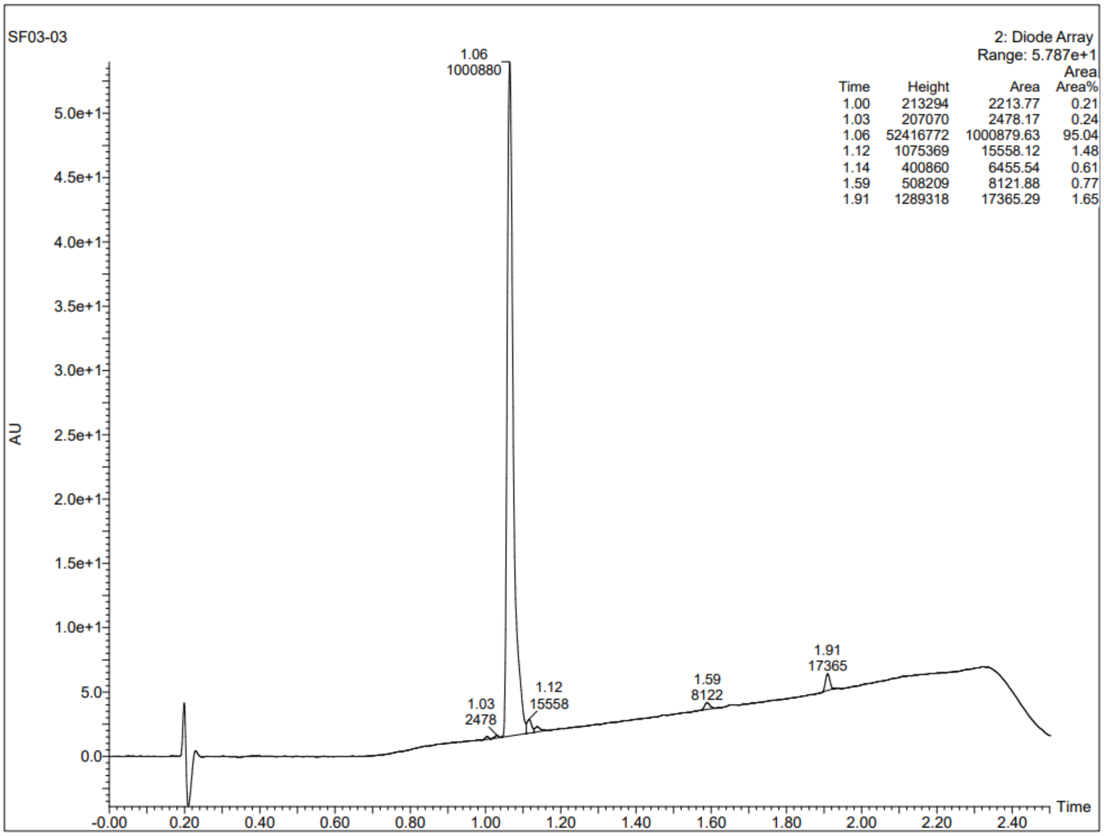

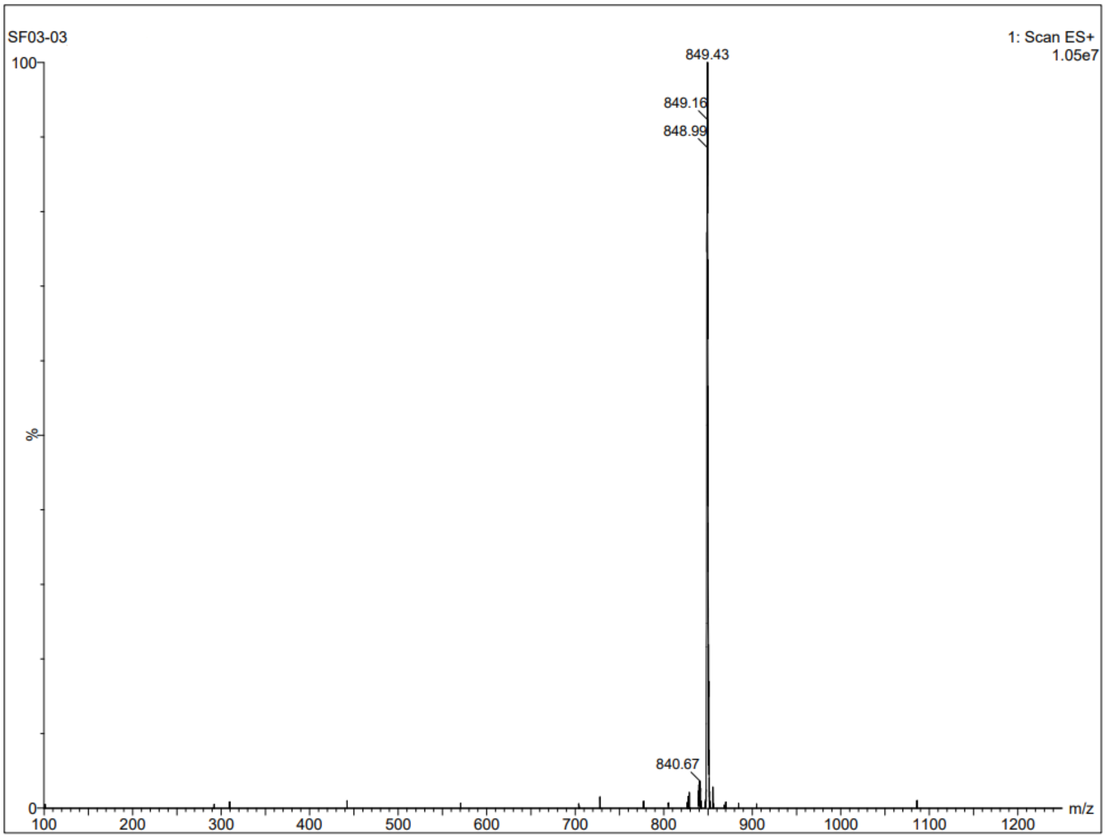

SF03-04: UPLC-MS

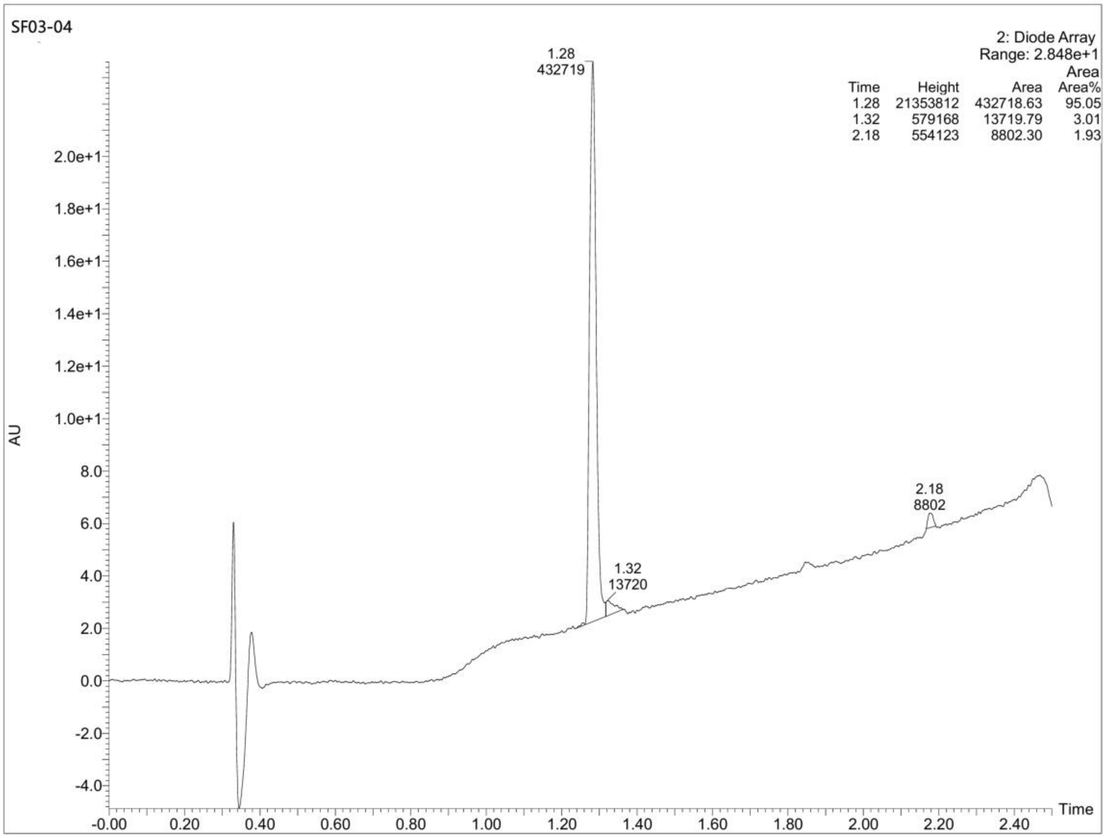

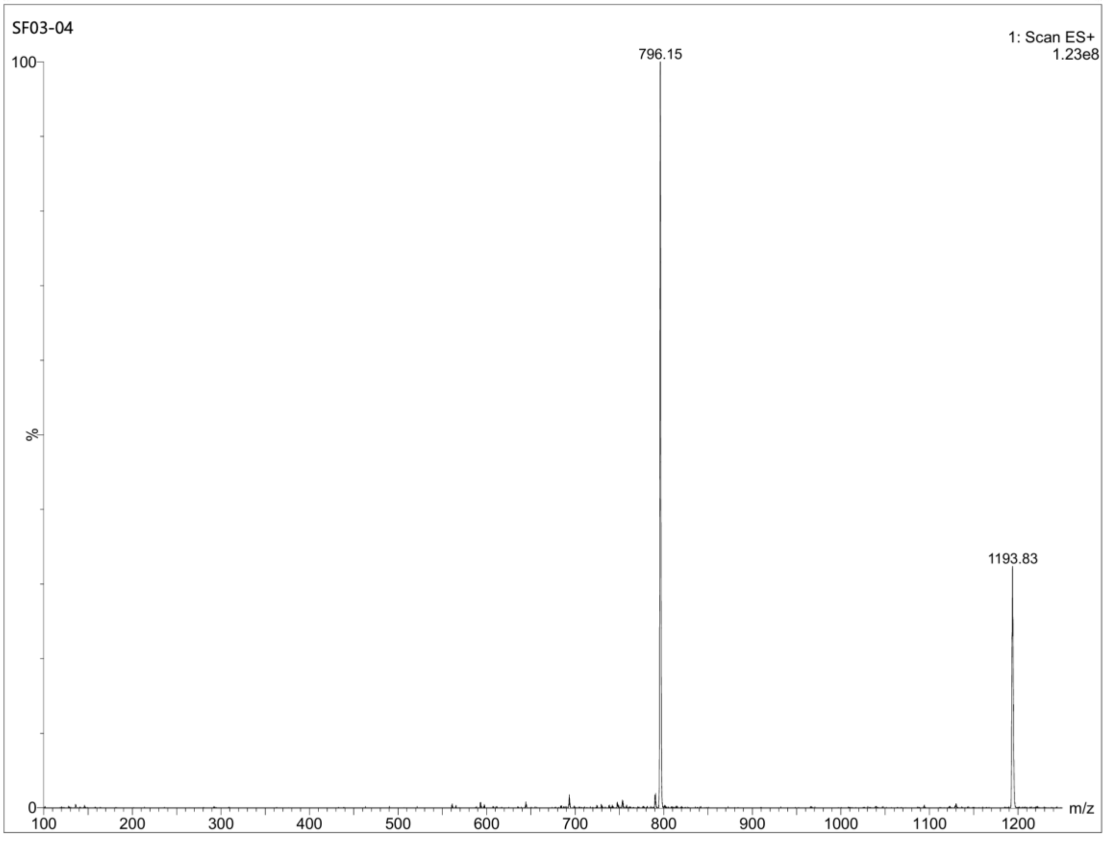

SF03-12: UPLC-MS

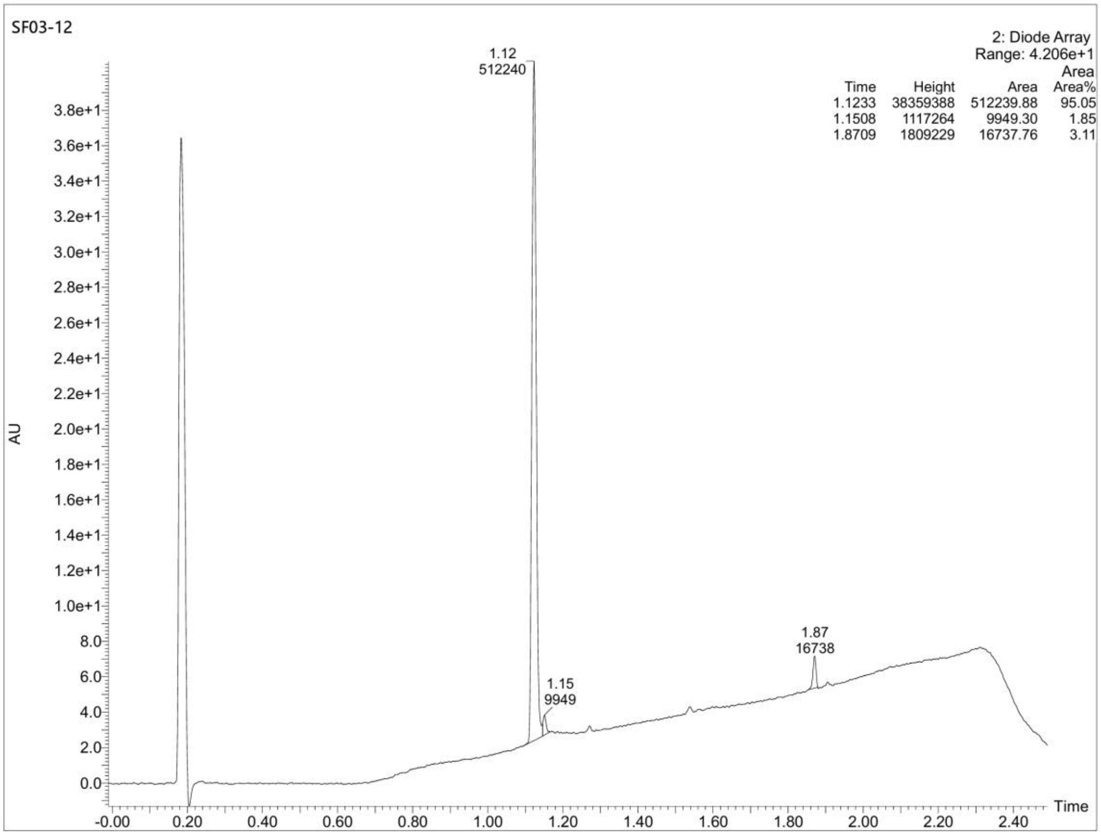

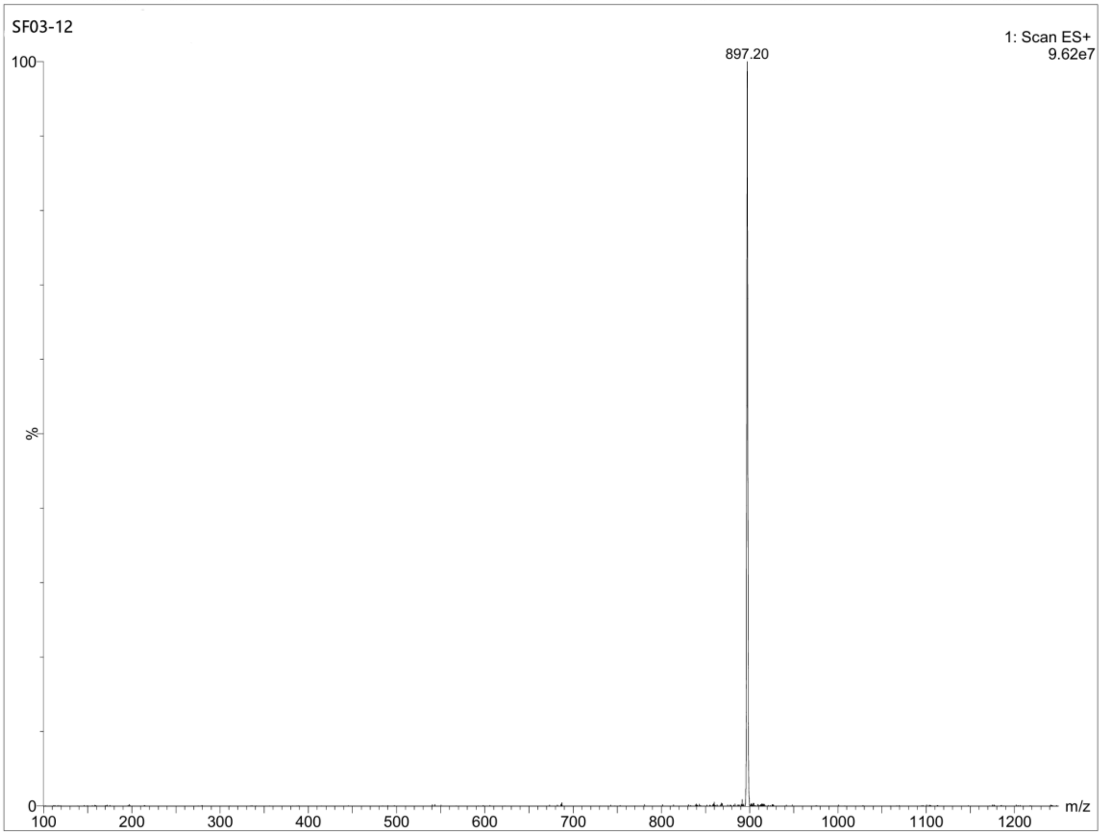

SF03-33: UPLC-MS

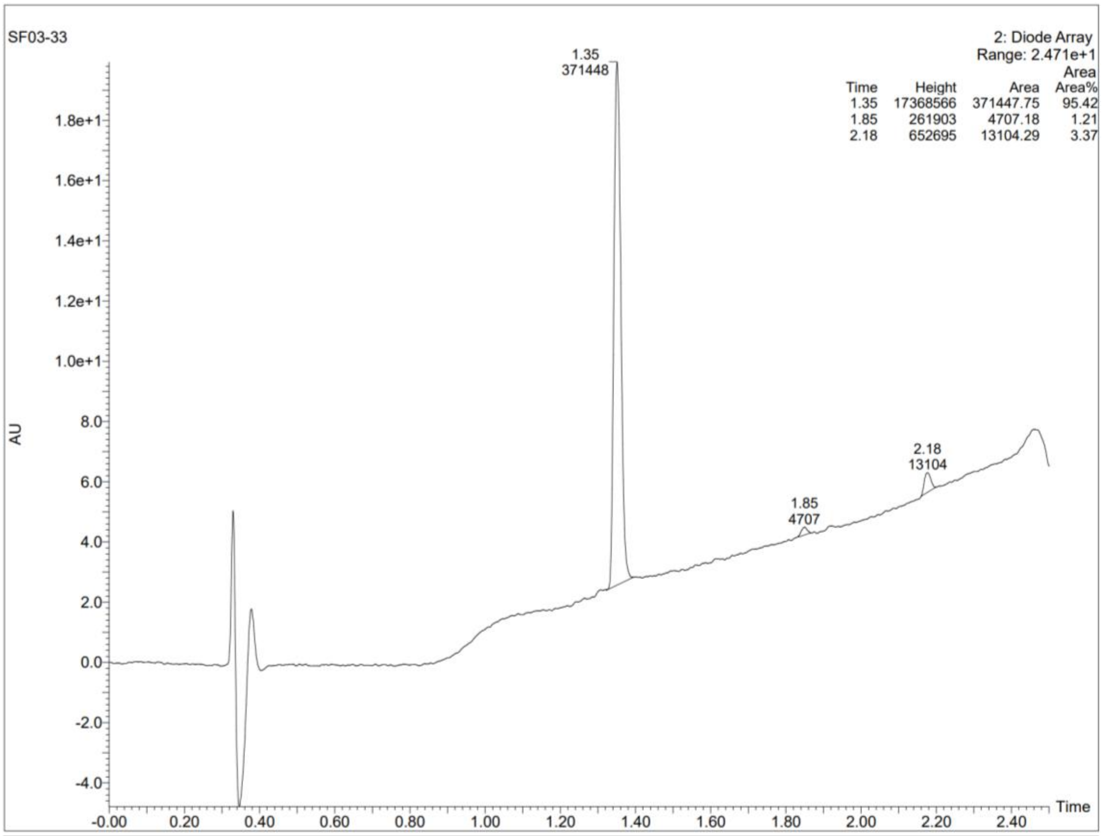

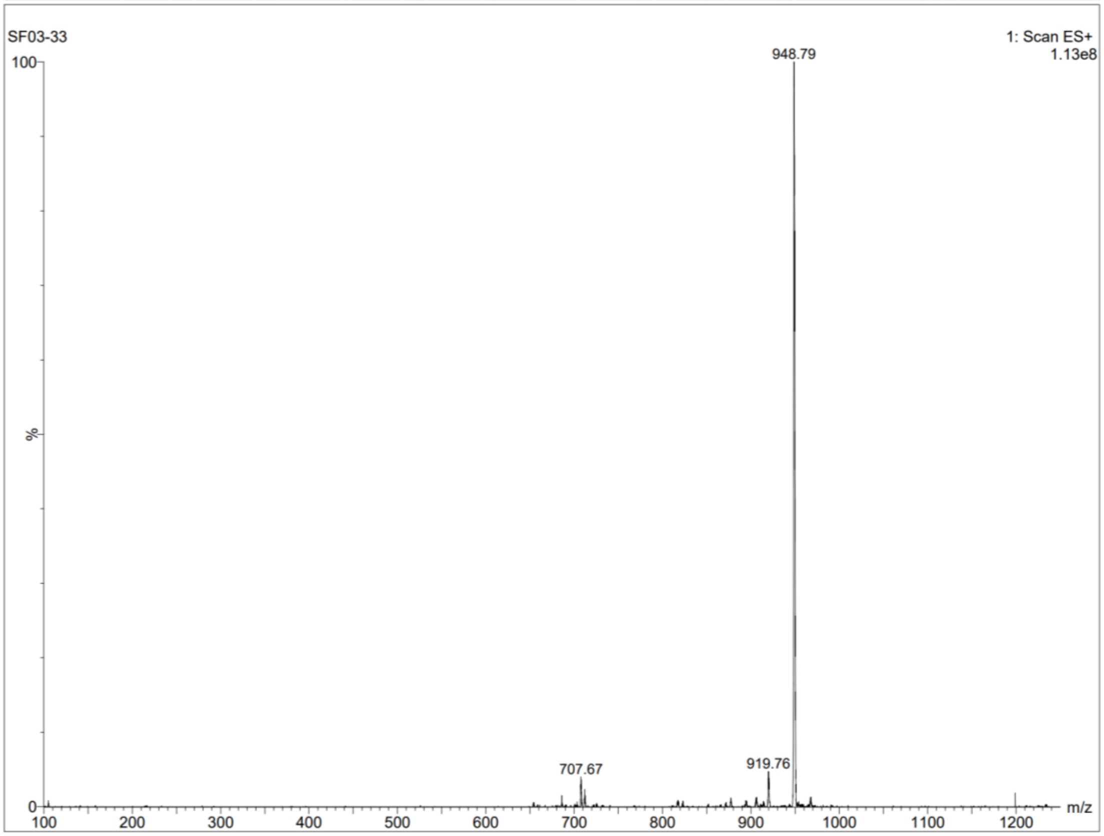

SF03-35: UPLC-MS

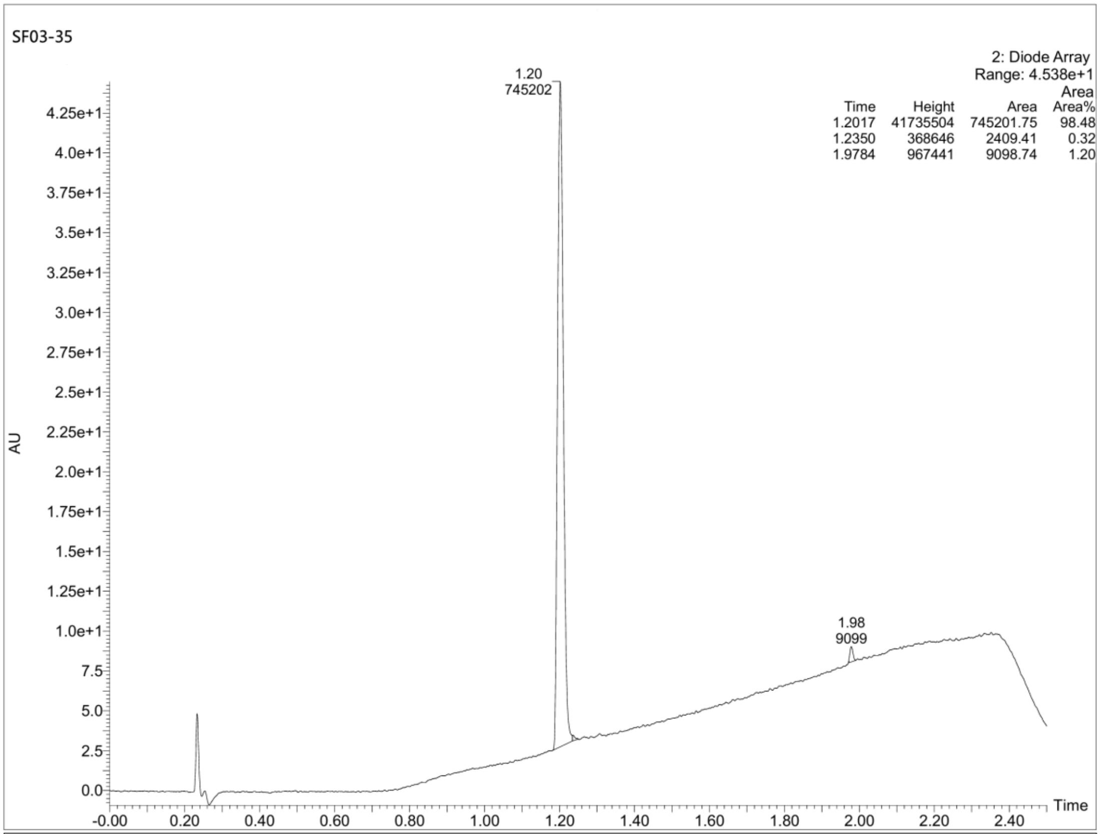

Time	Height	Area	Area Area%
1.2017	41735504	745201.75	98.48
1.2350	368646	2409.41	0.32
1.9784	967441	9098.74	1.20

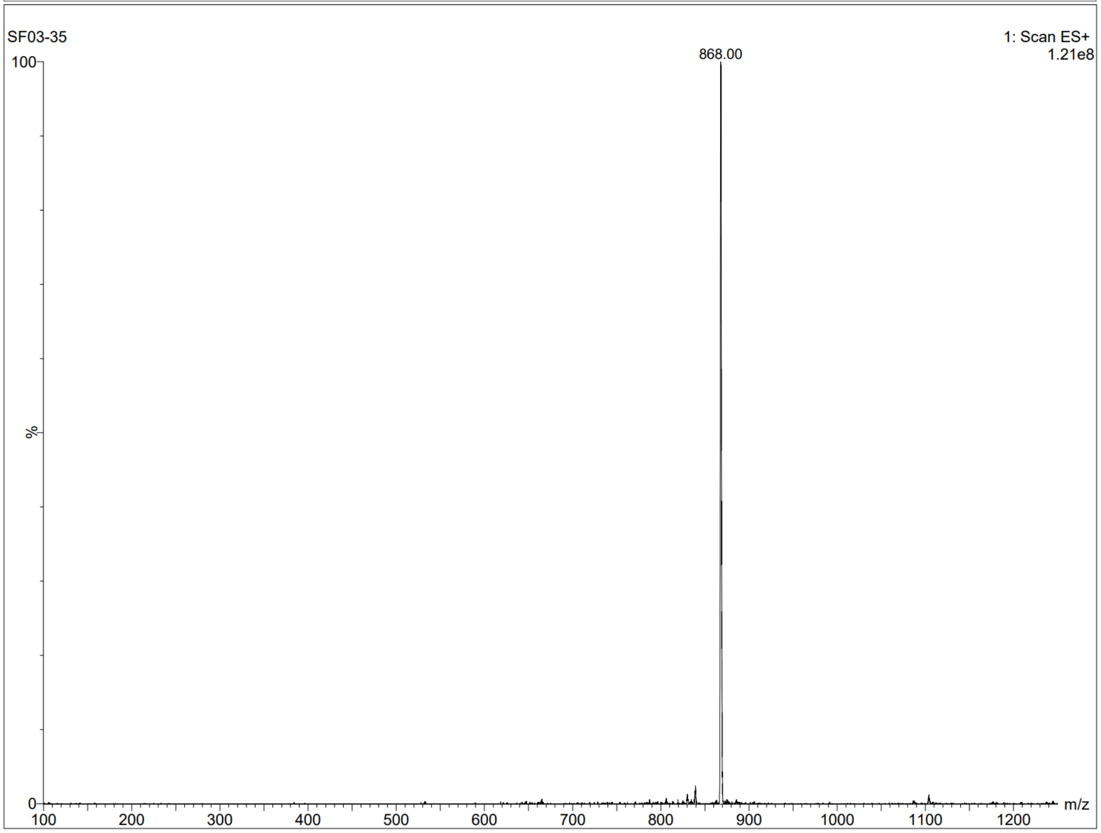

SF03-37: UPLC-MS

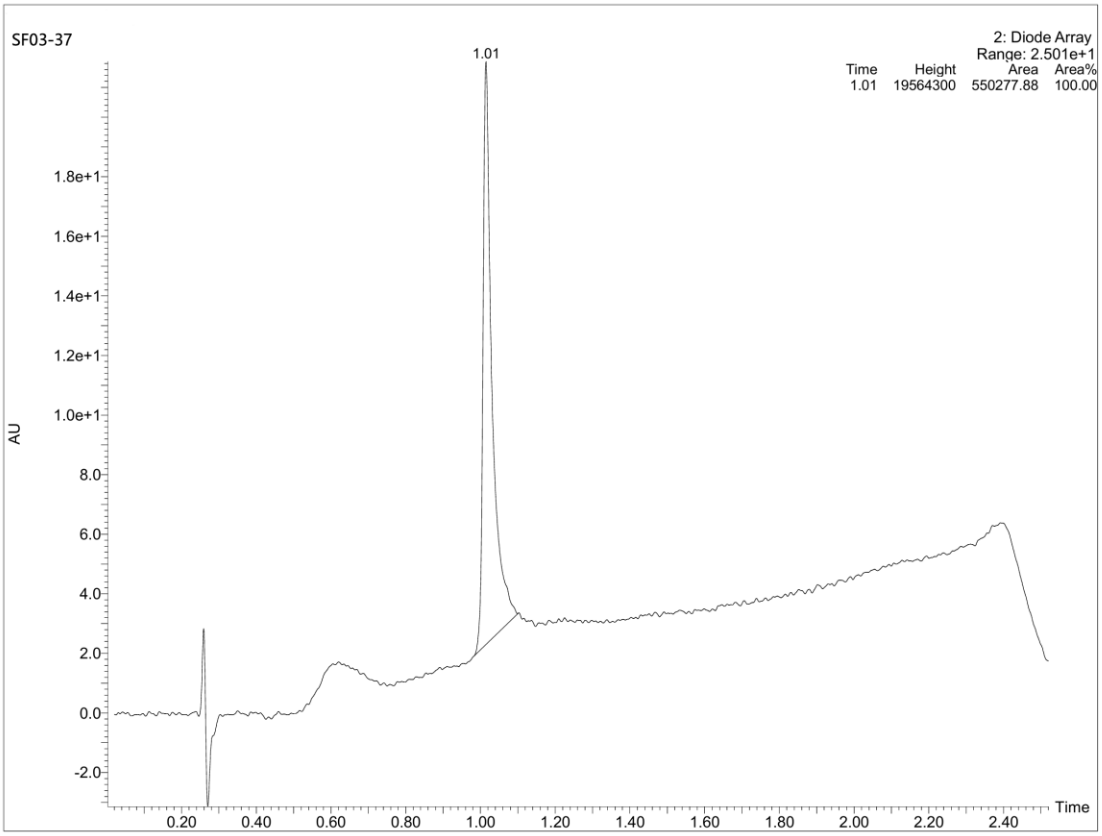

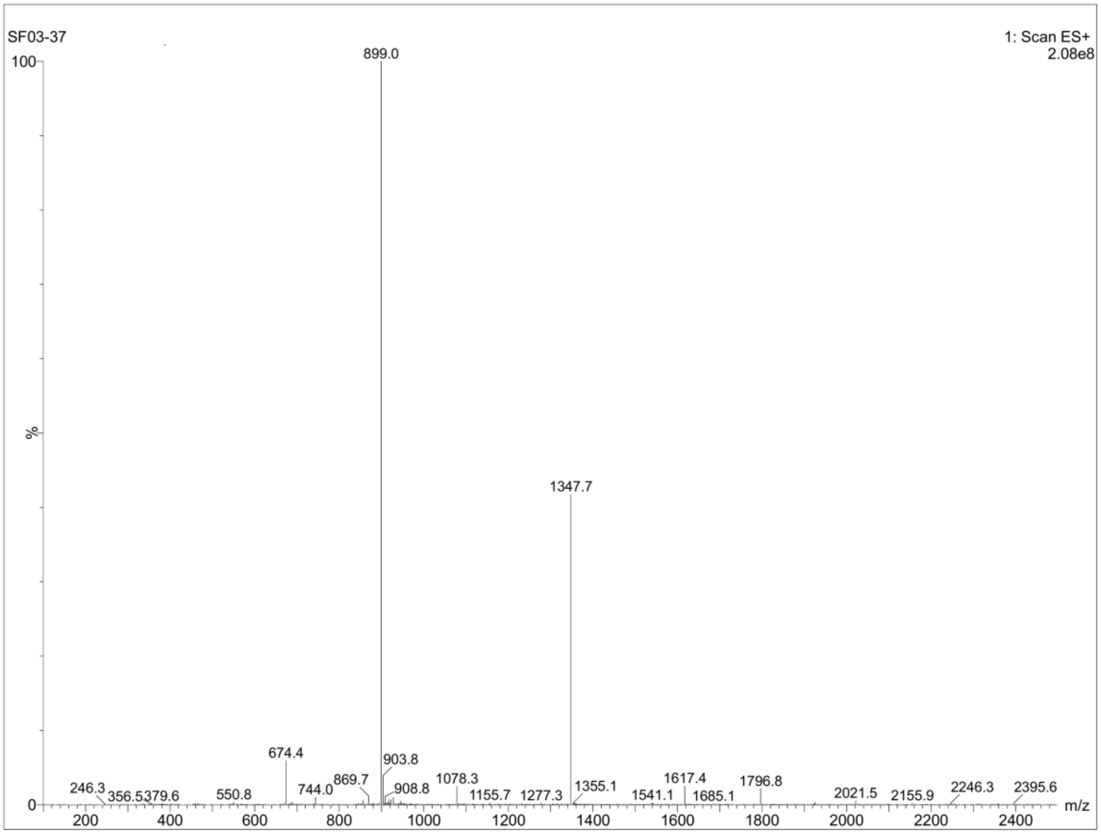

SF03-34: UPLC-MS

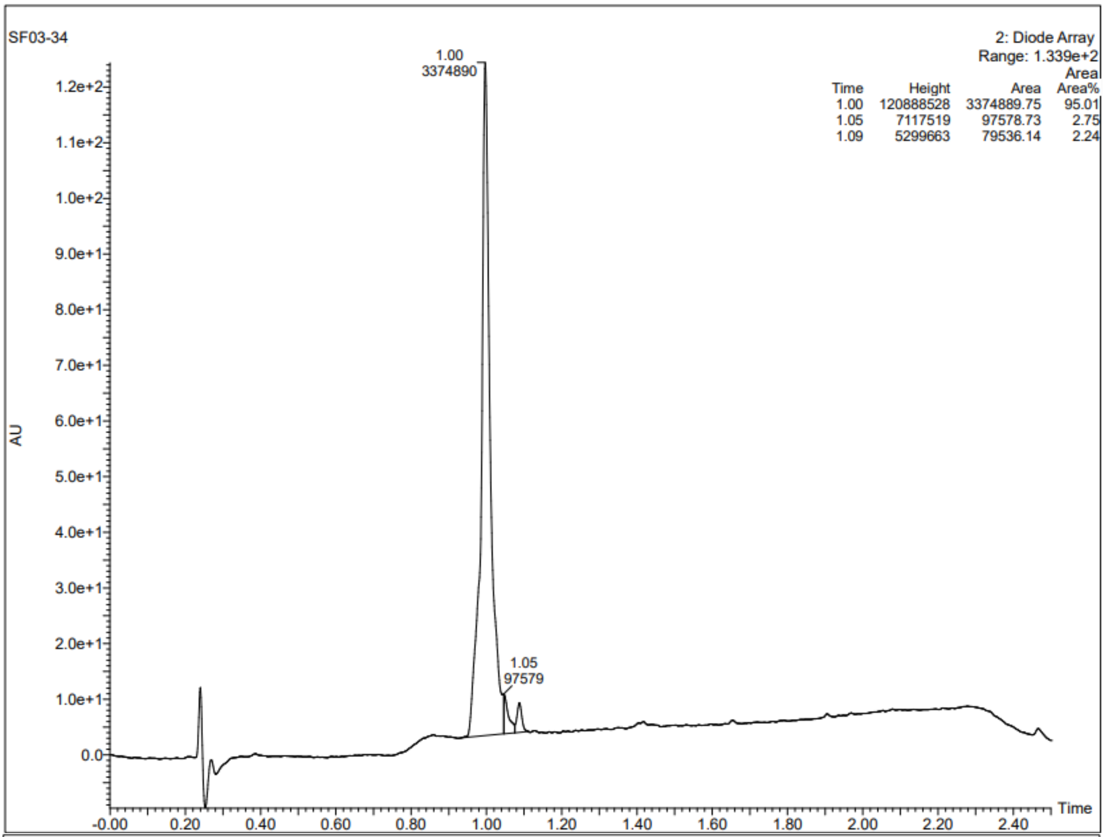

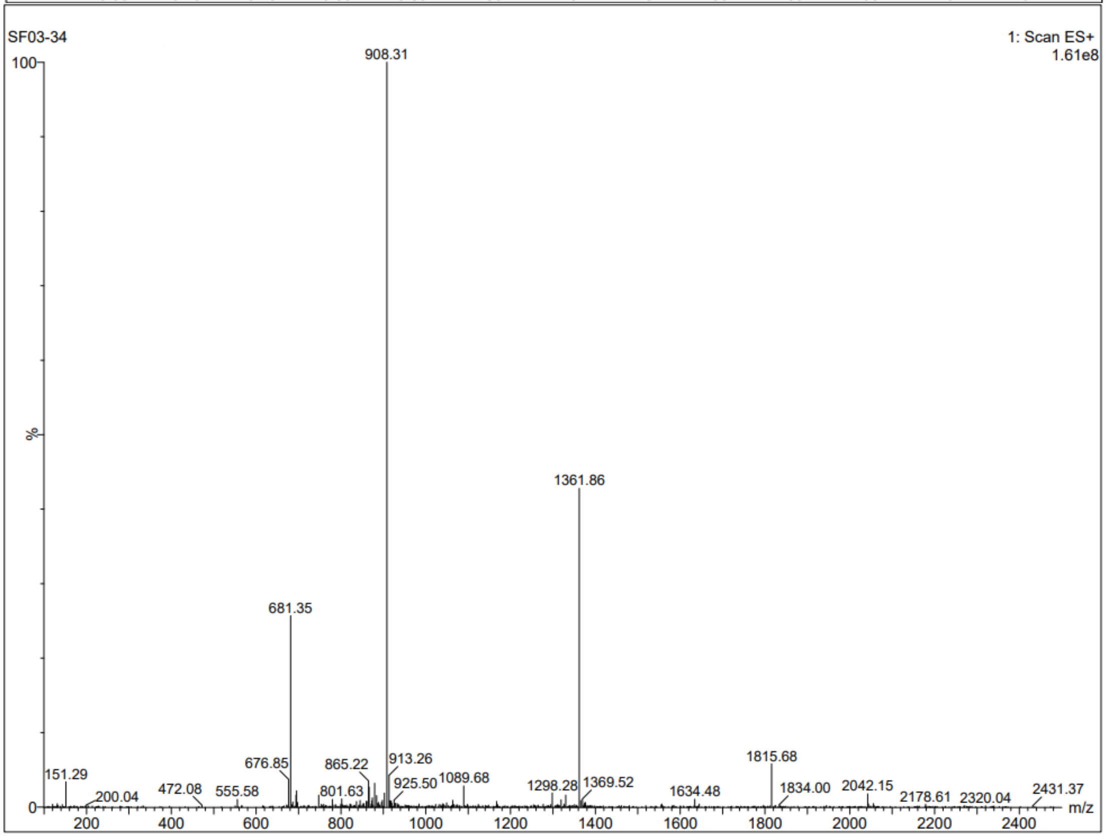

SF03-38: UPLC-MS

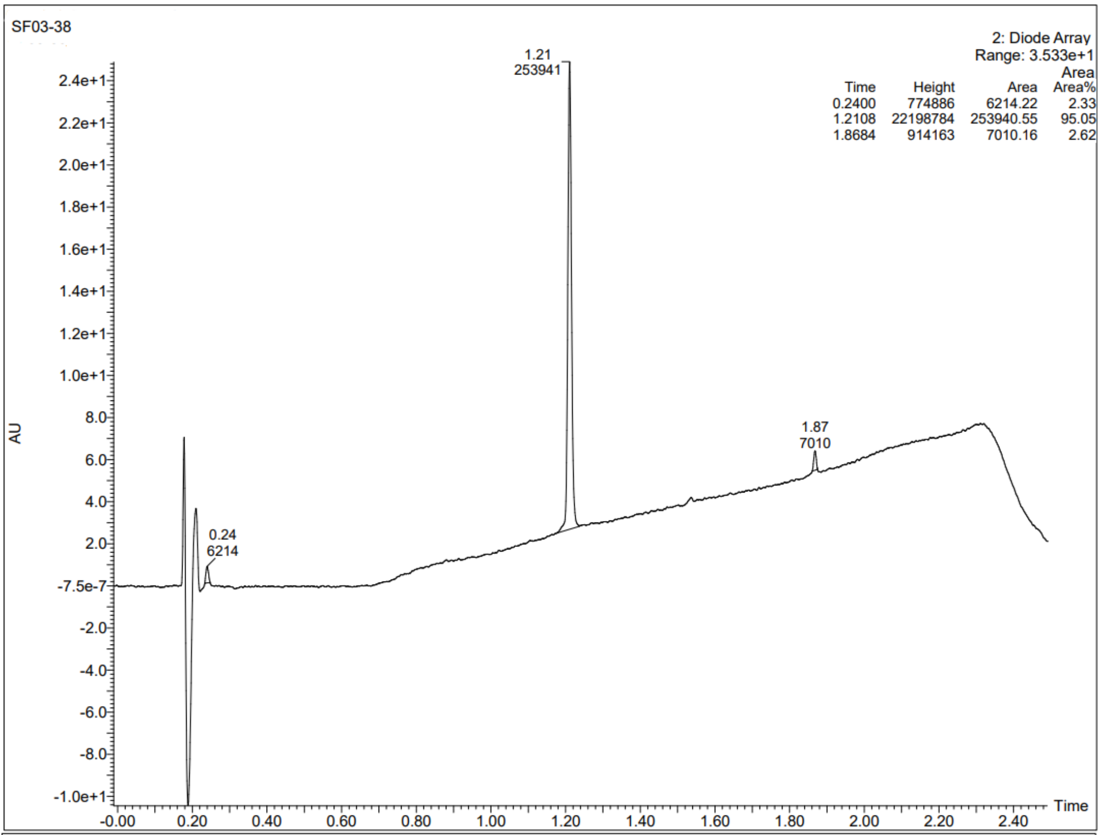

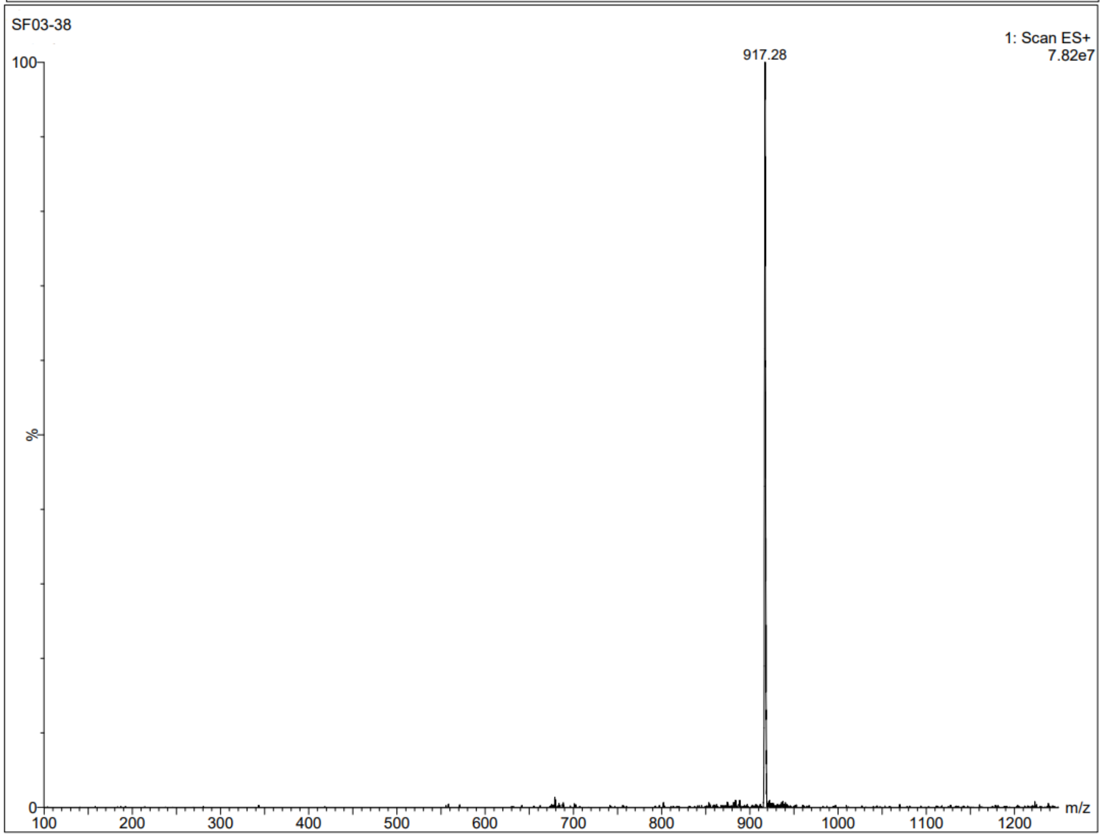

SF03-68: UPLC-MS

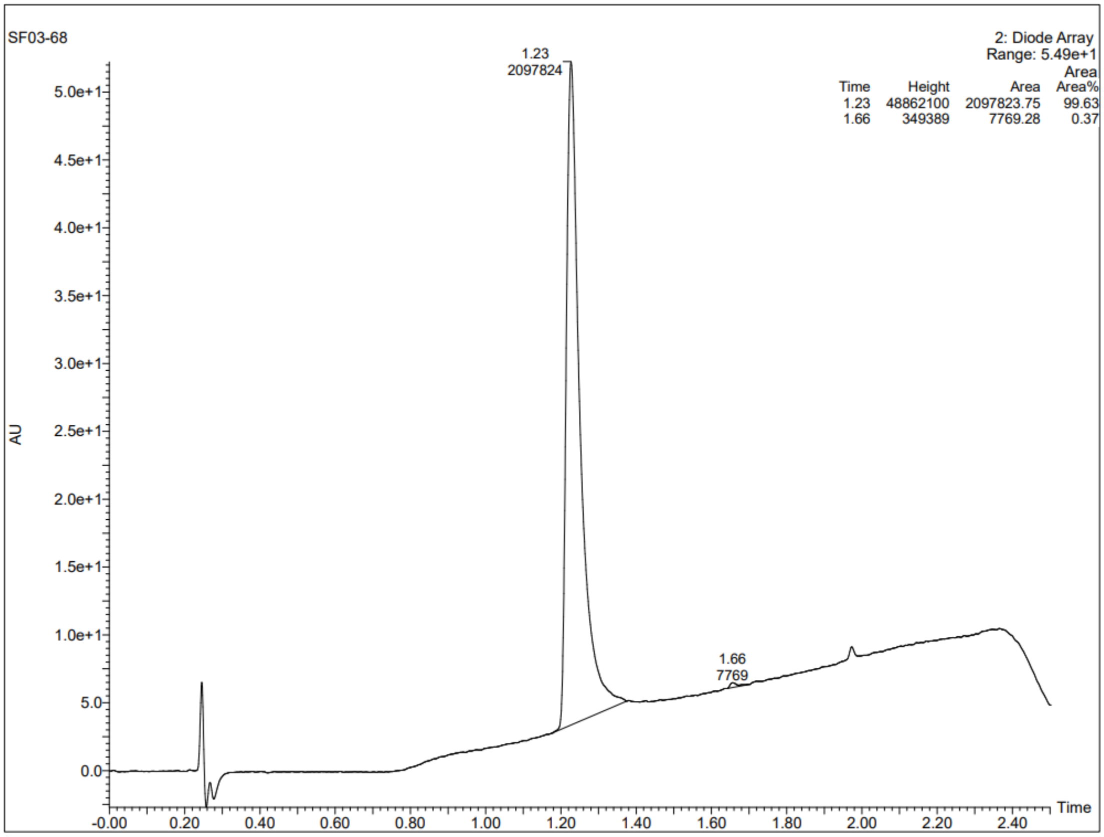

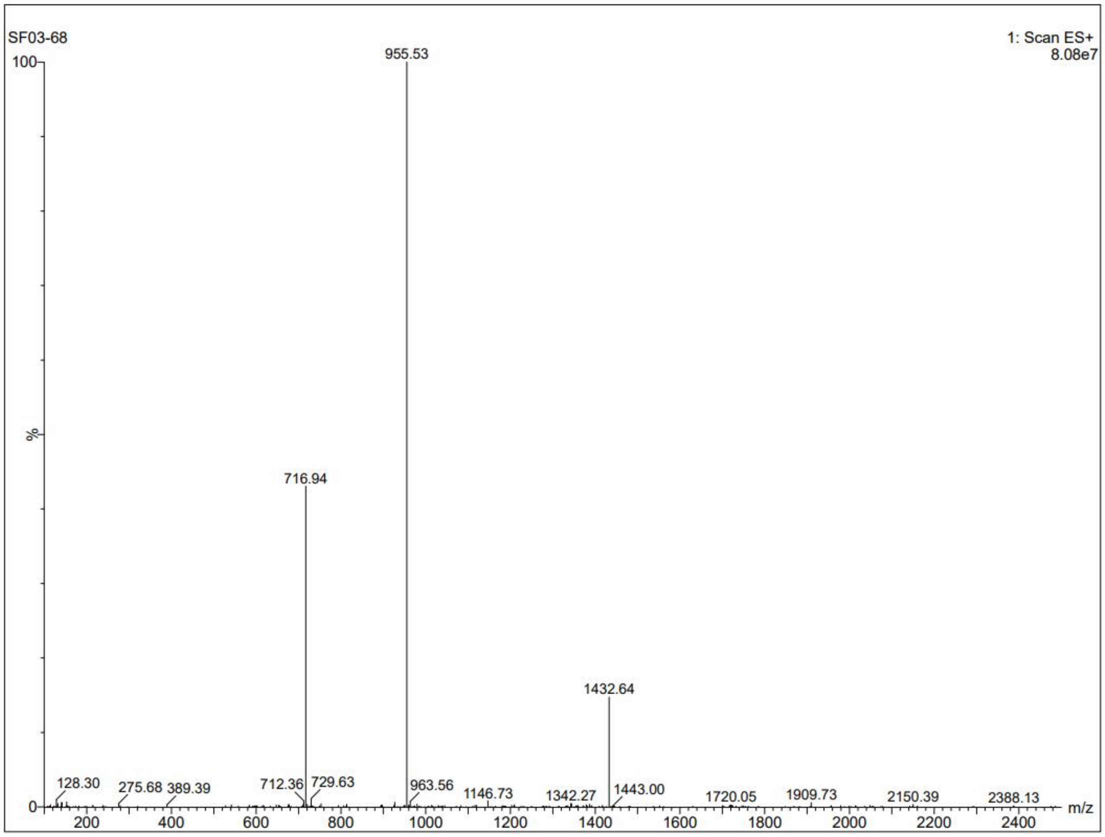

SF03-52: UPLC-MS

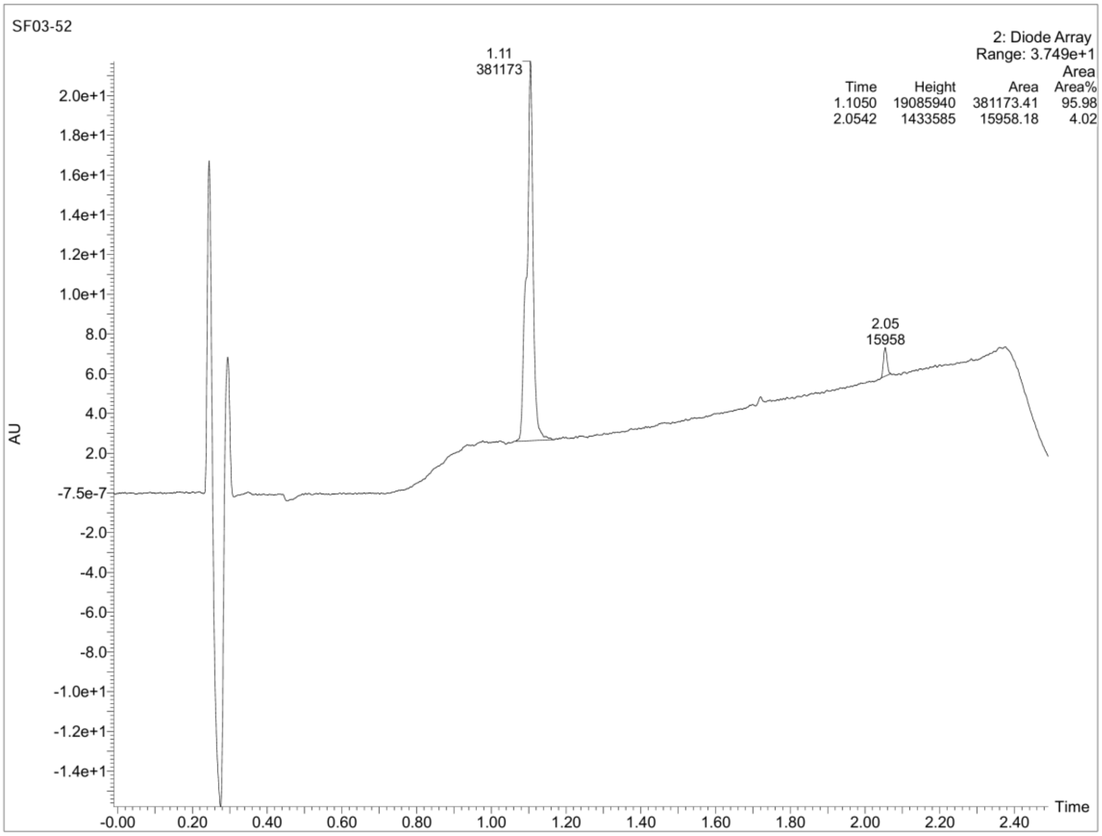

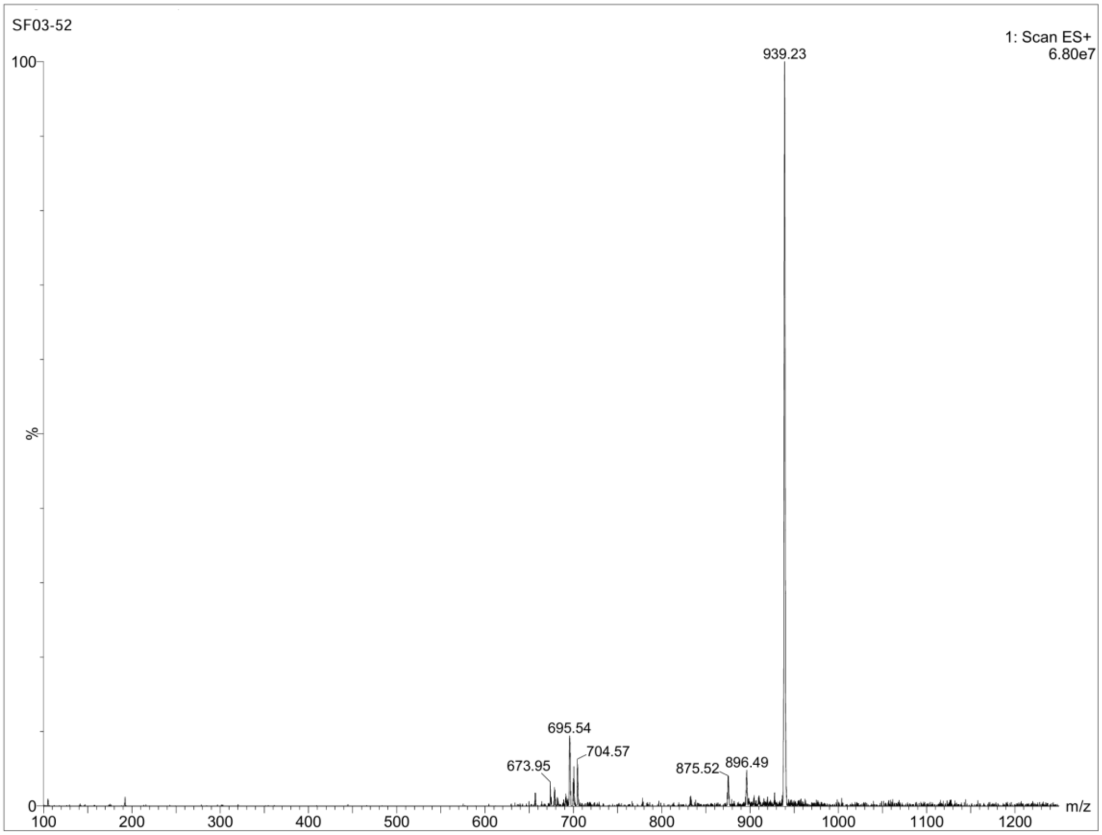

SF04-01: UPLC-MS

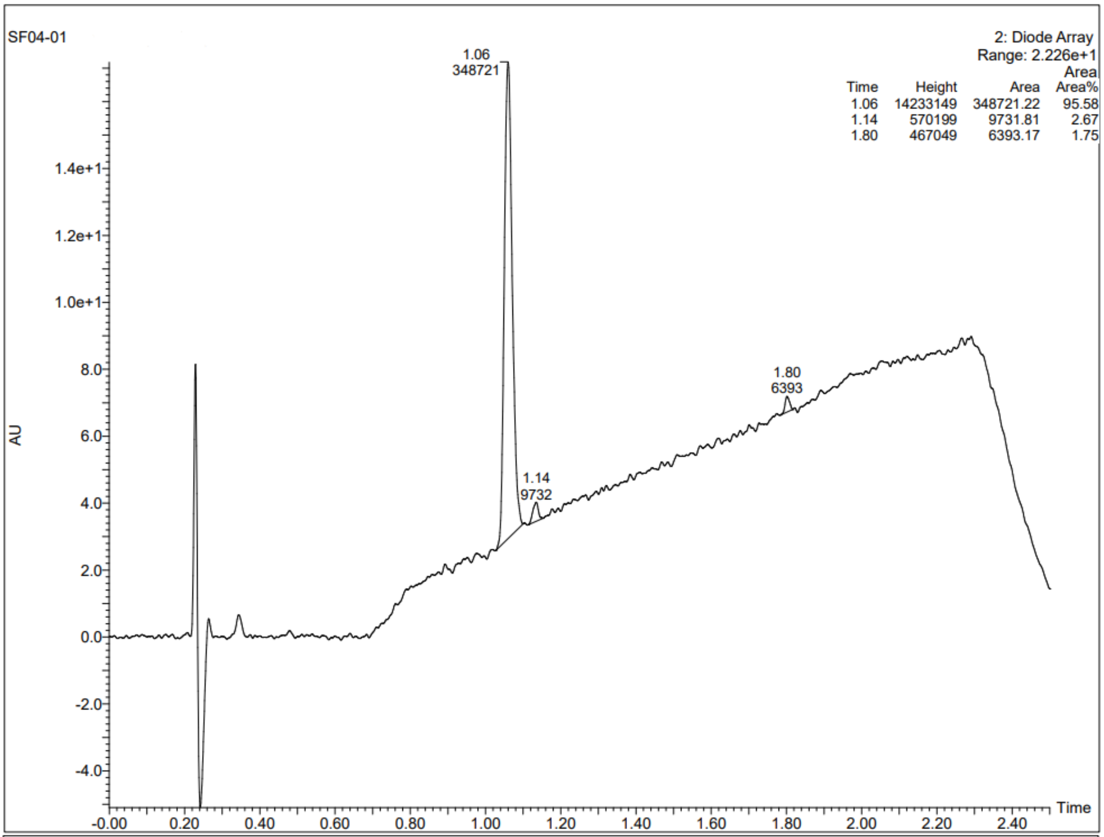

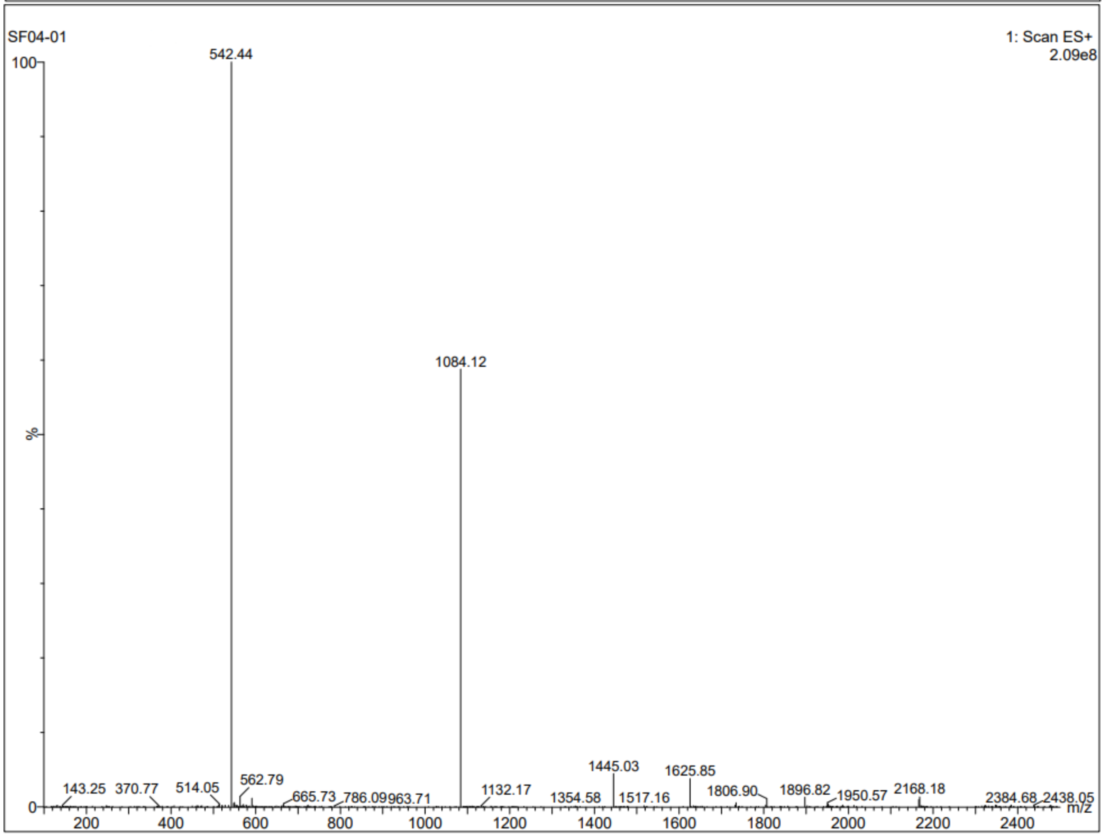

SF04-02: UPLC-MS

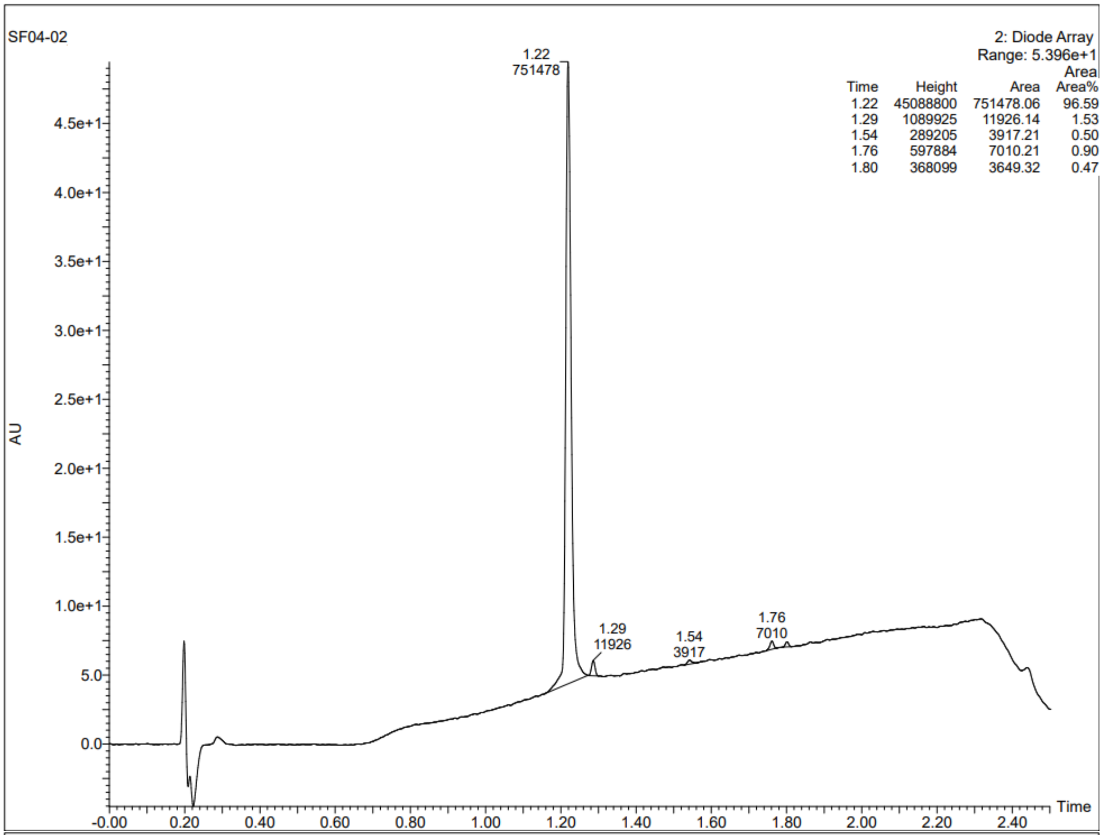

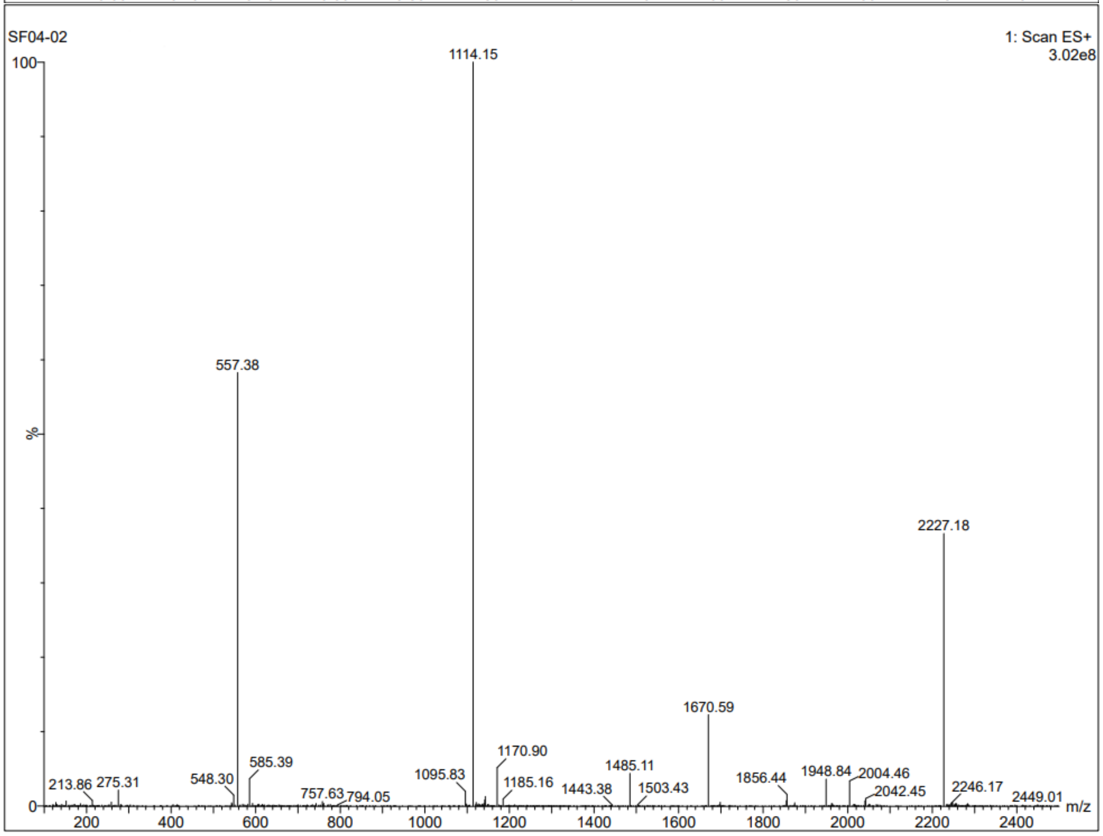

SF04-03: UPLC-MS

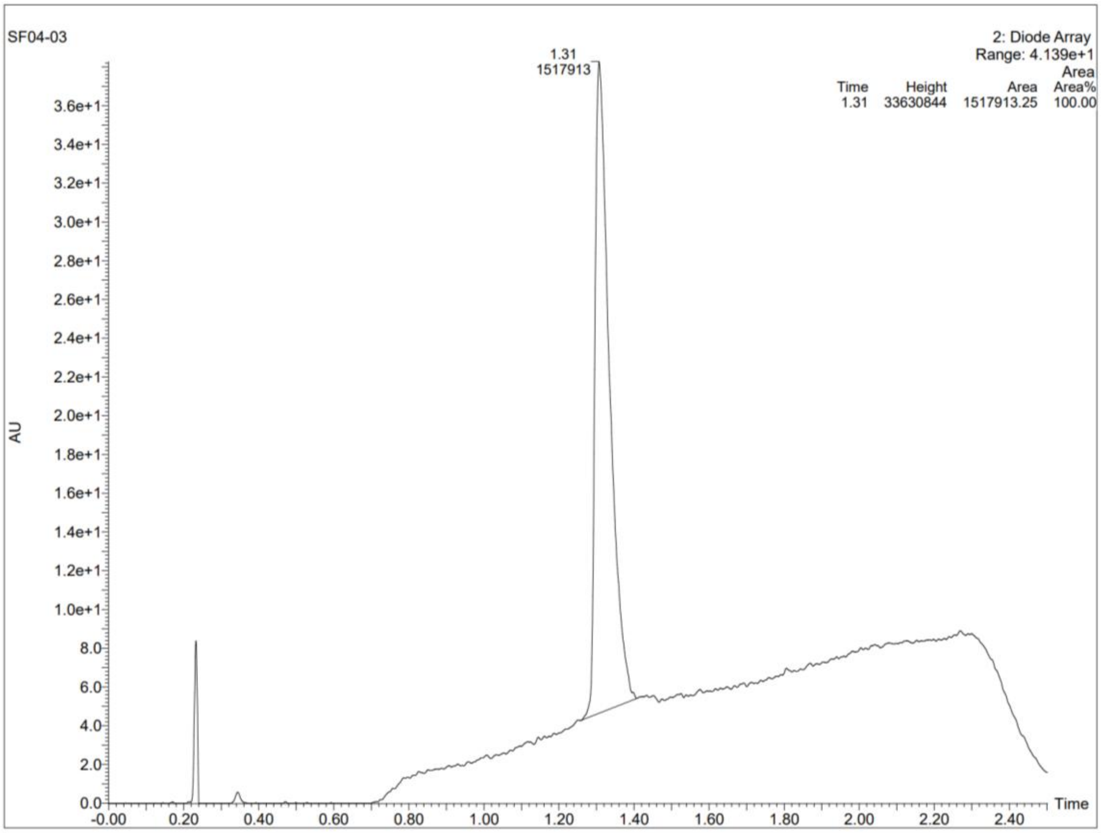

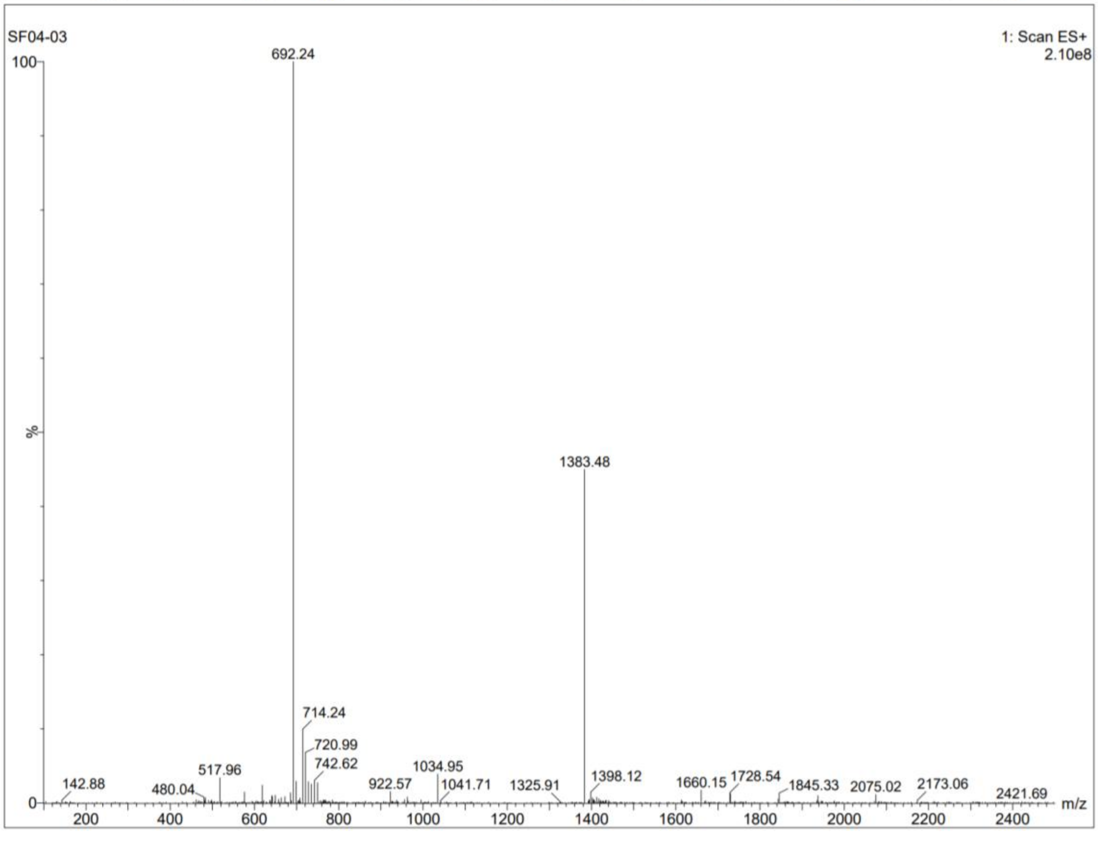

SF04-04: UPLC-MS

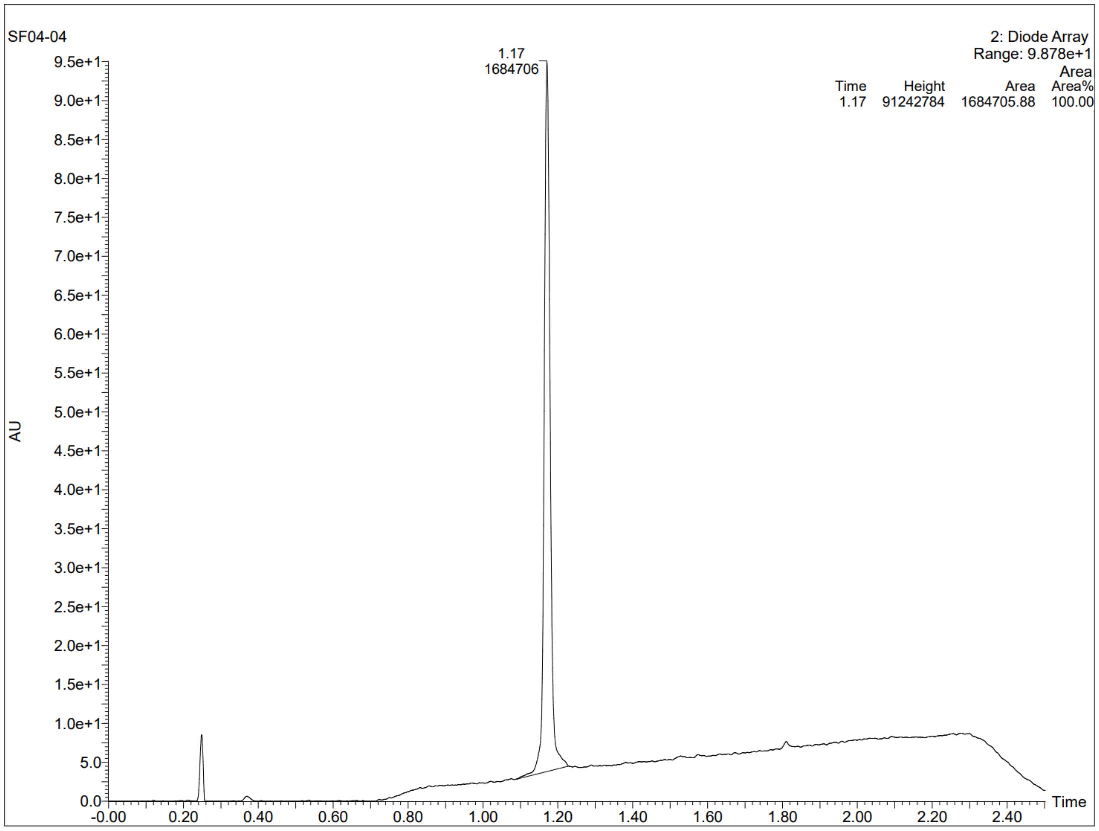

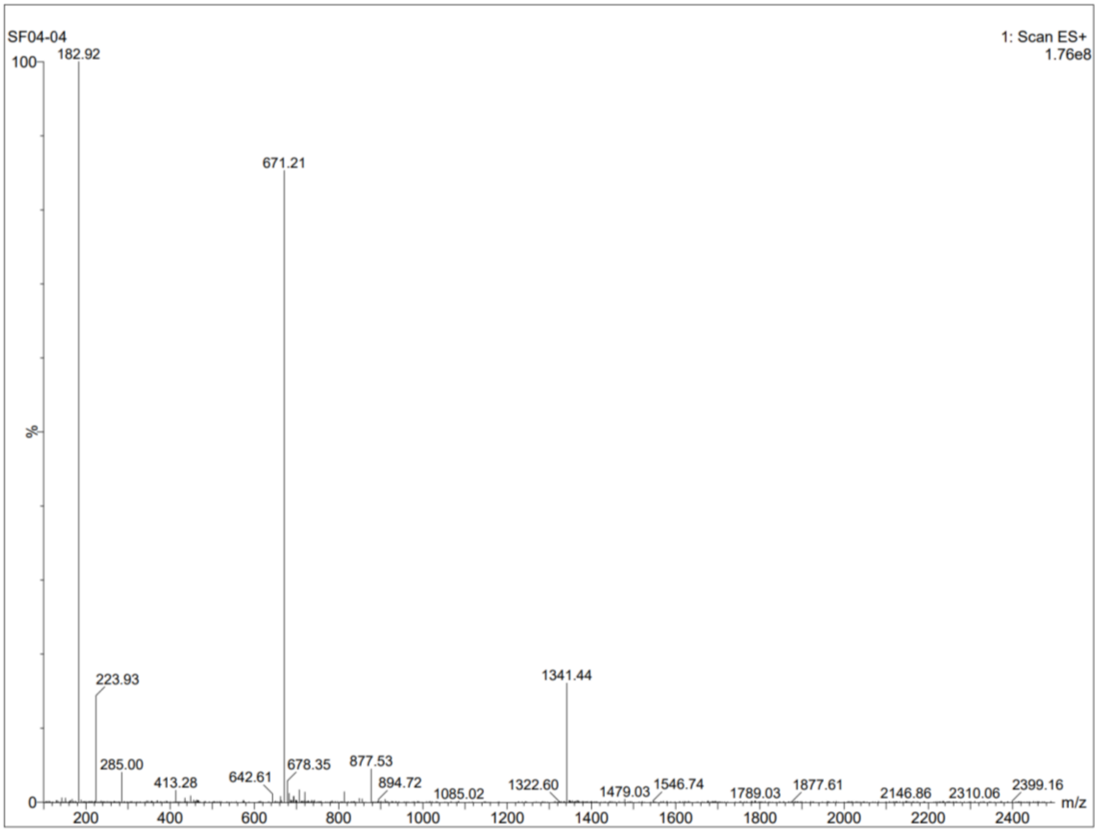

SF04-07: UPLC-MS

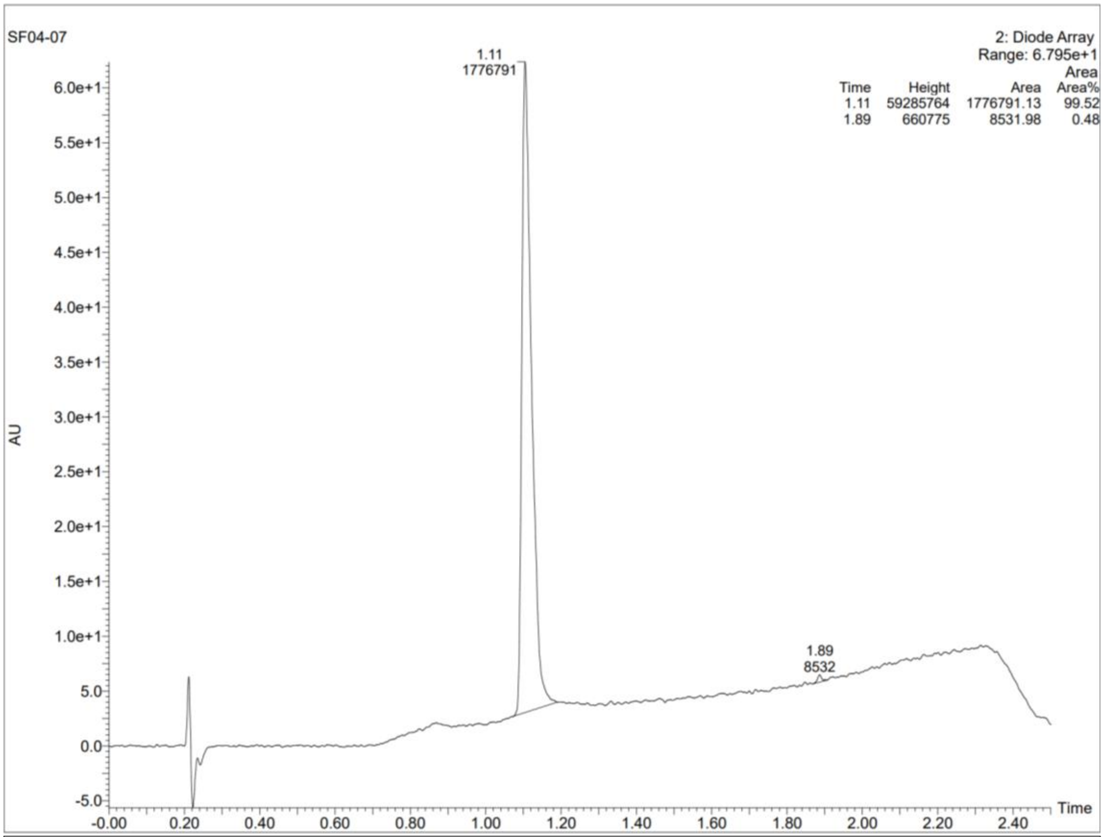

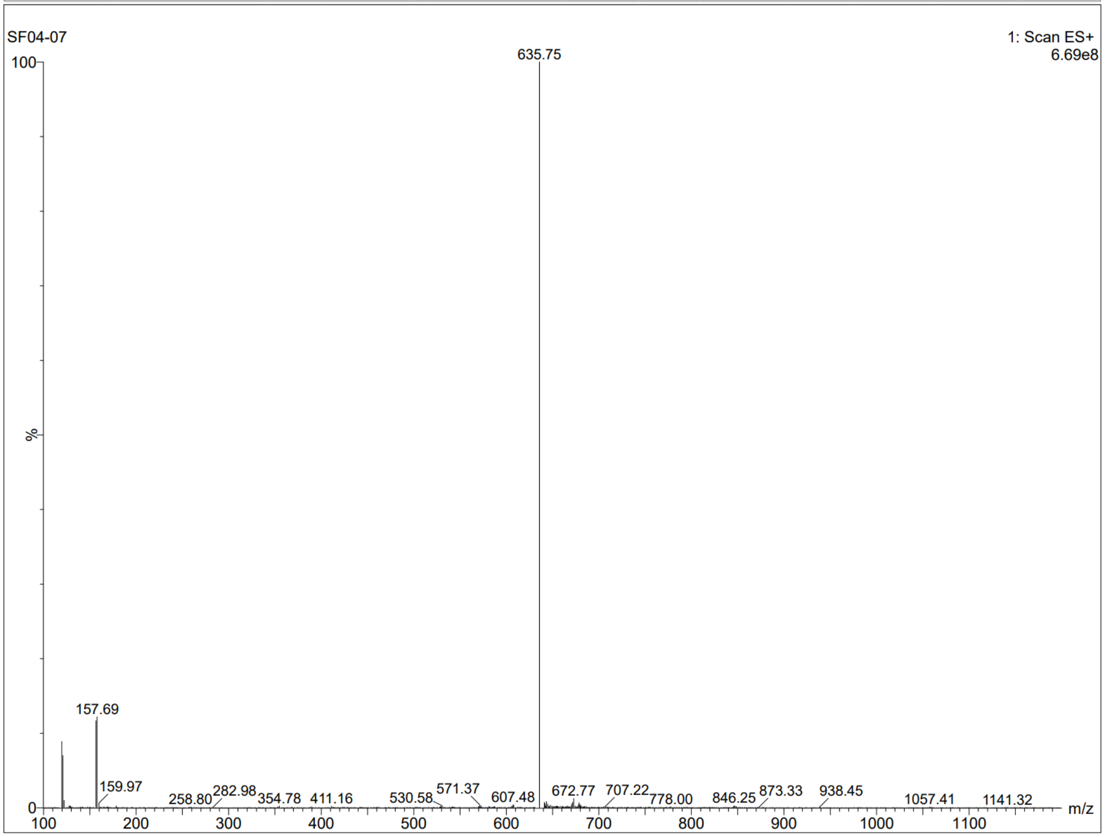

SF04-08: UPLC-MS

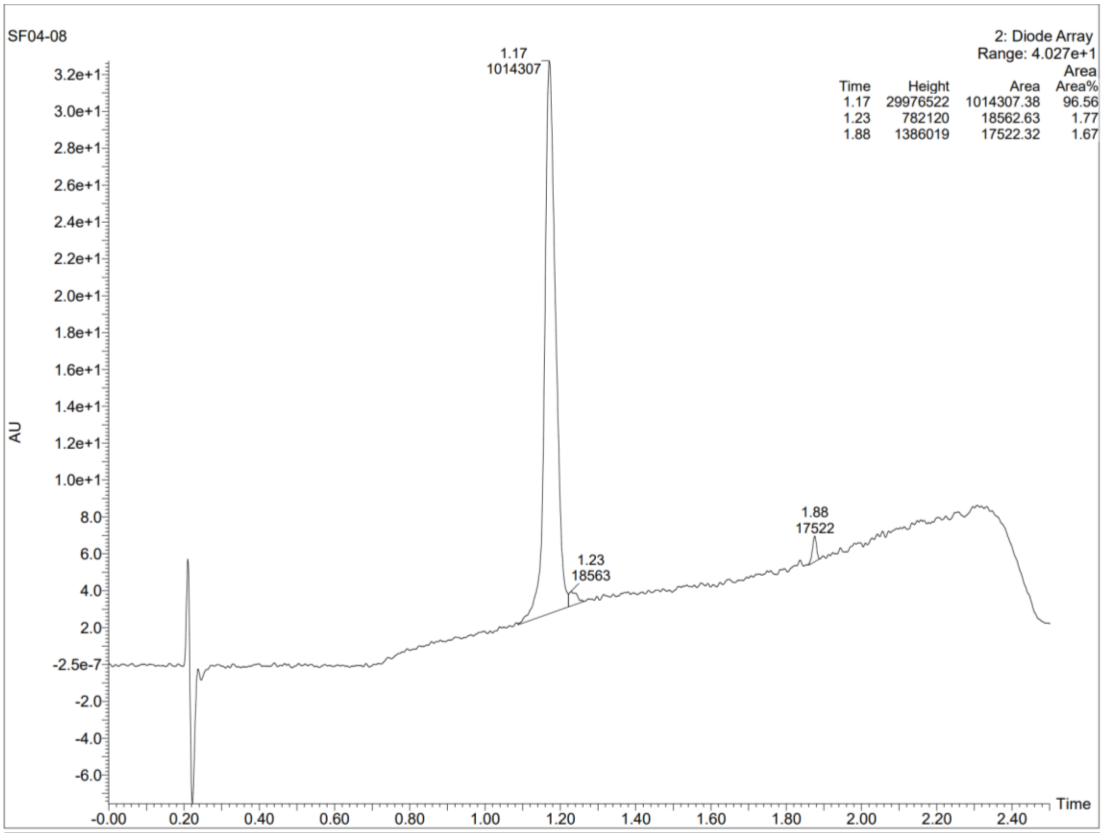

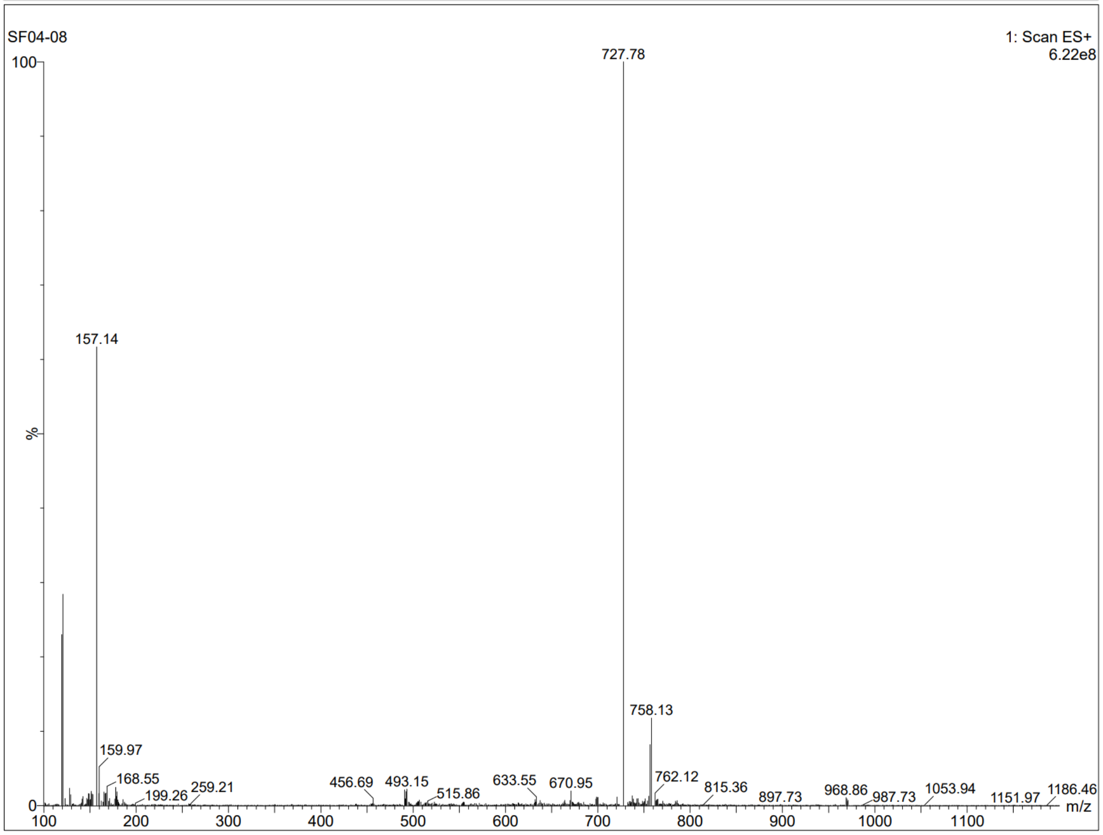

SF04-09: UPLC-MS

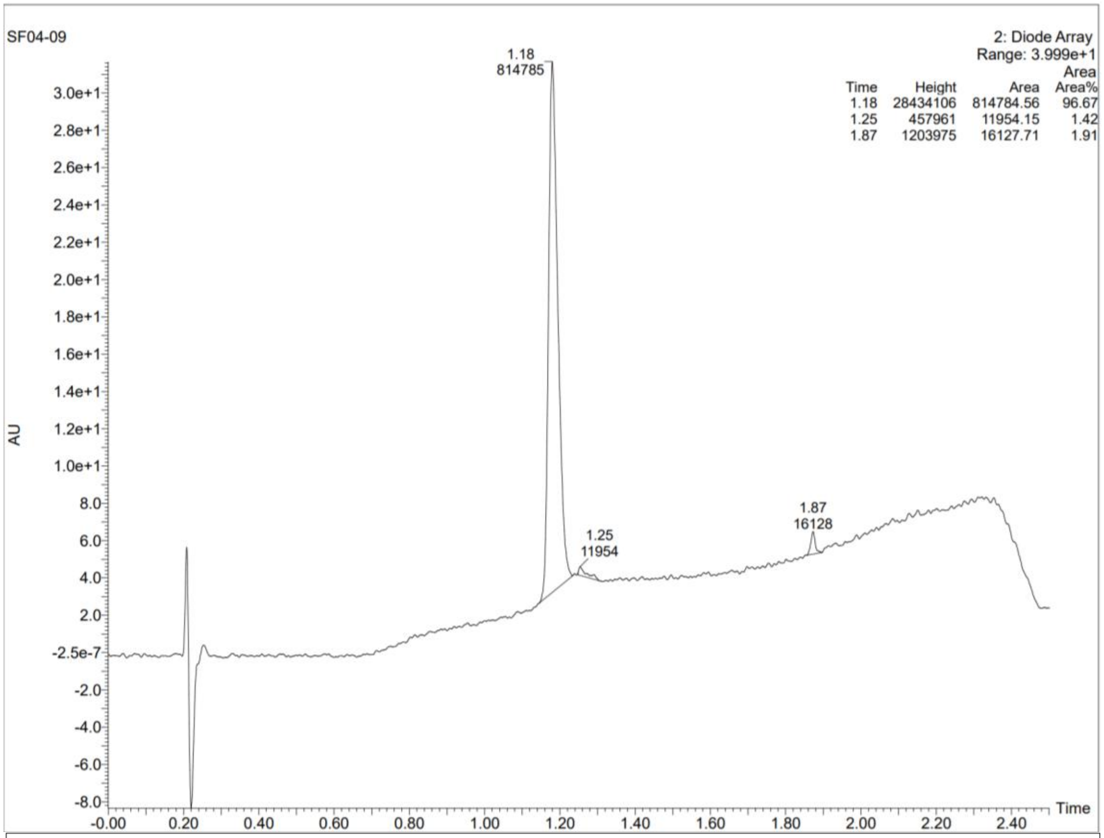

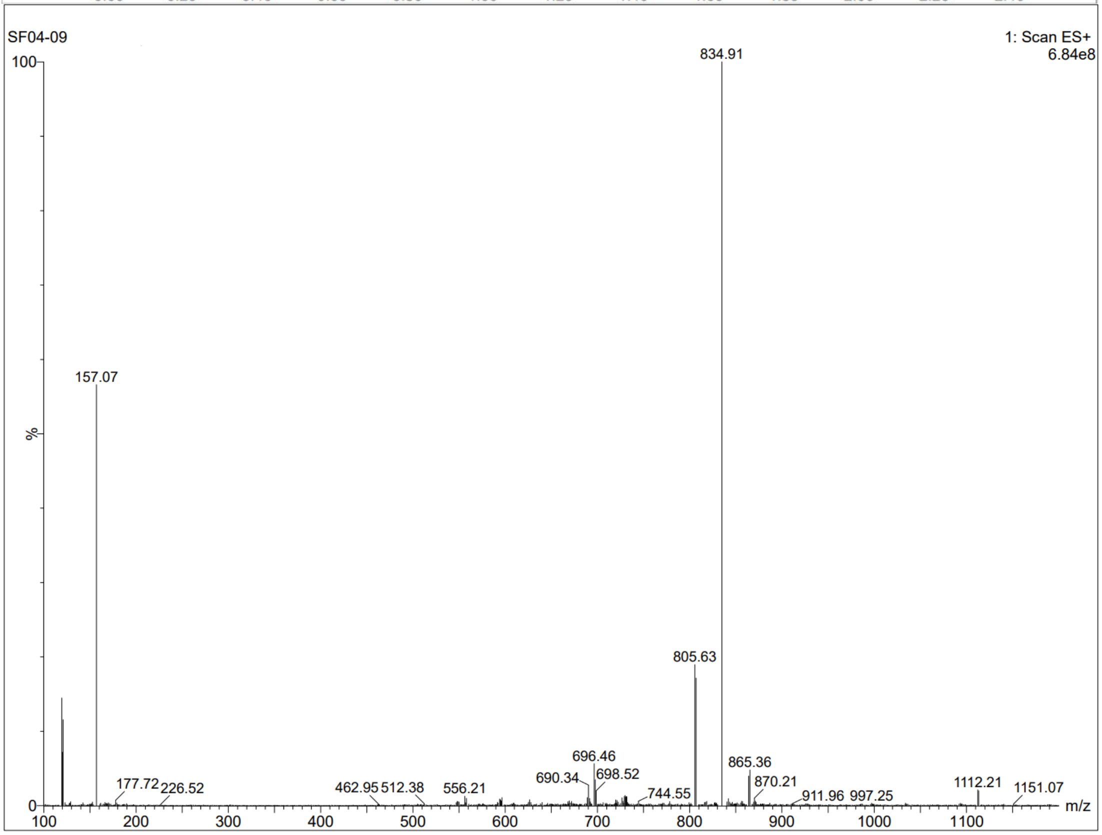

SF04-10: UPLC-MS

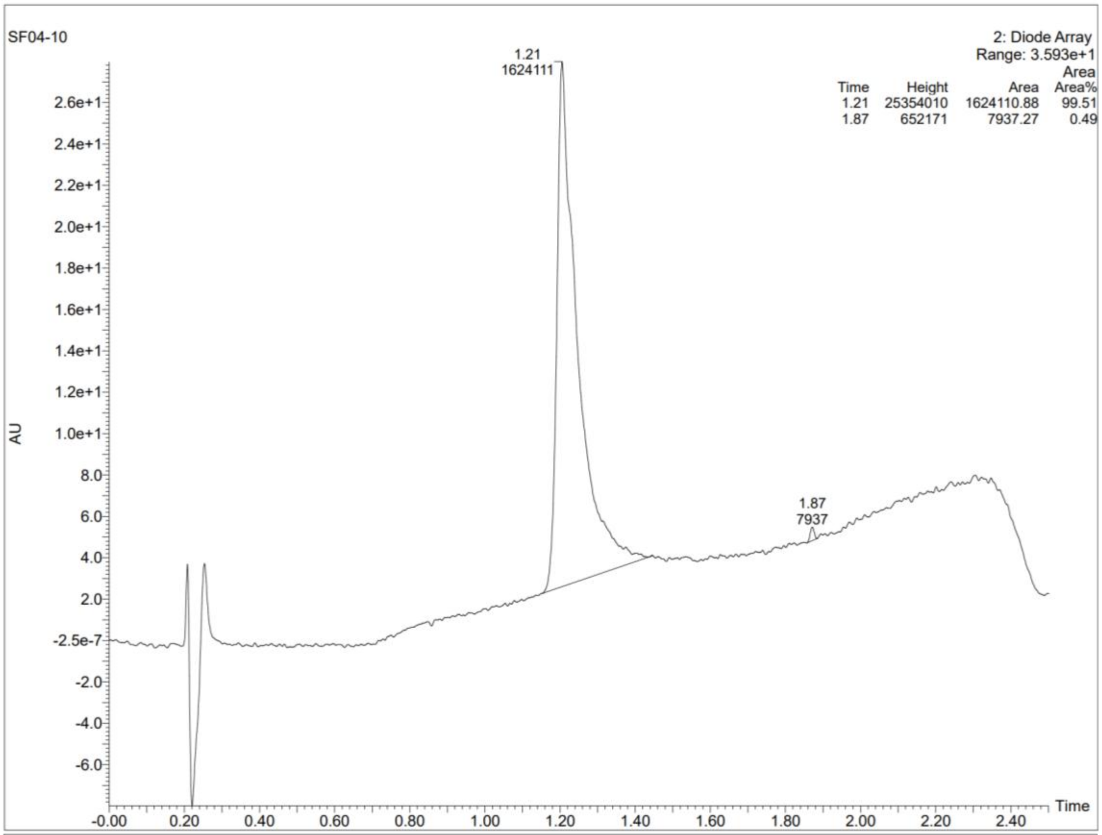

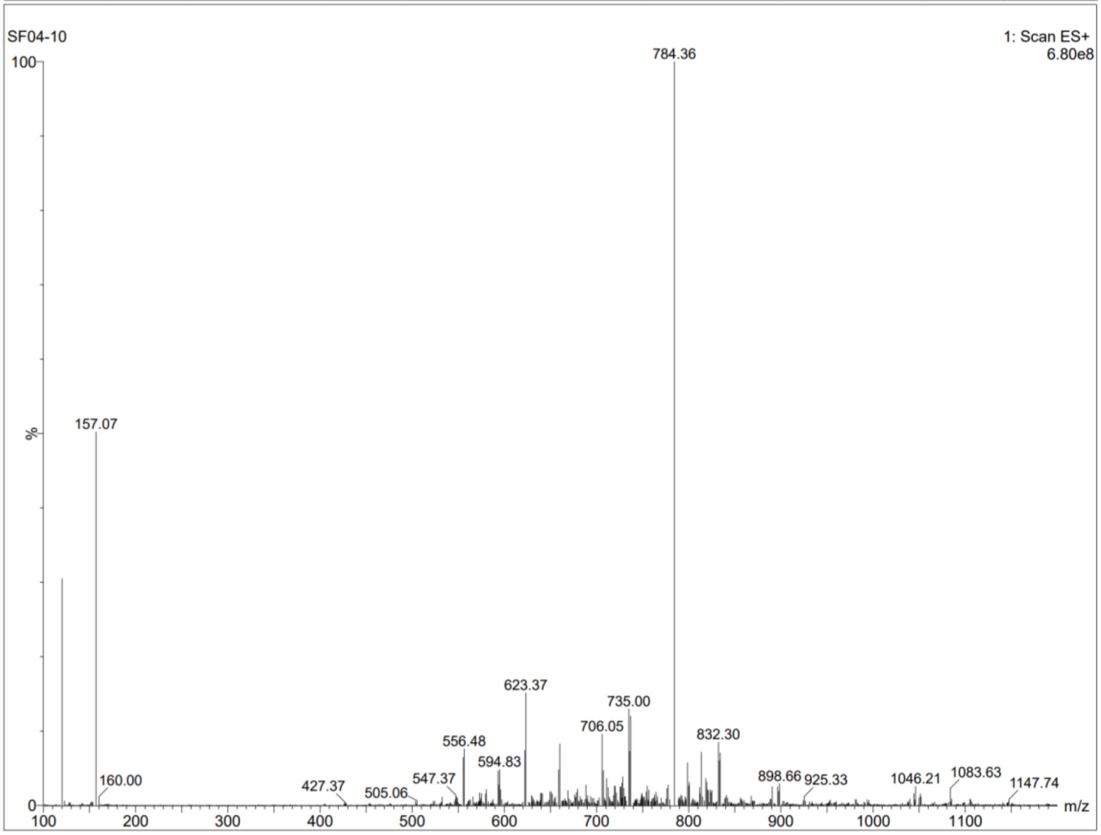

SF04-11: UPLC-MS

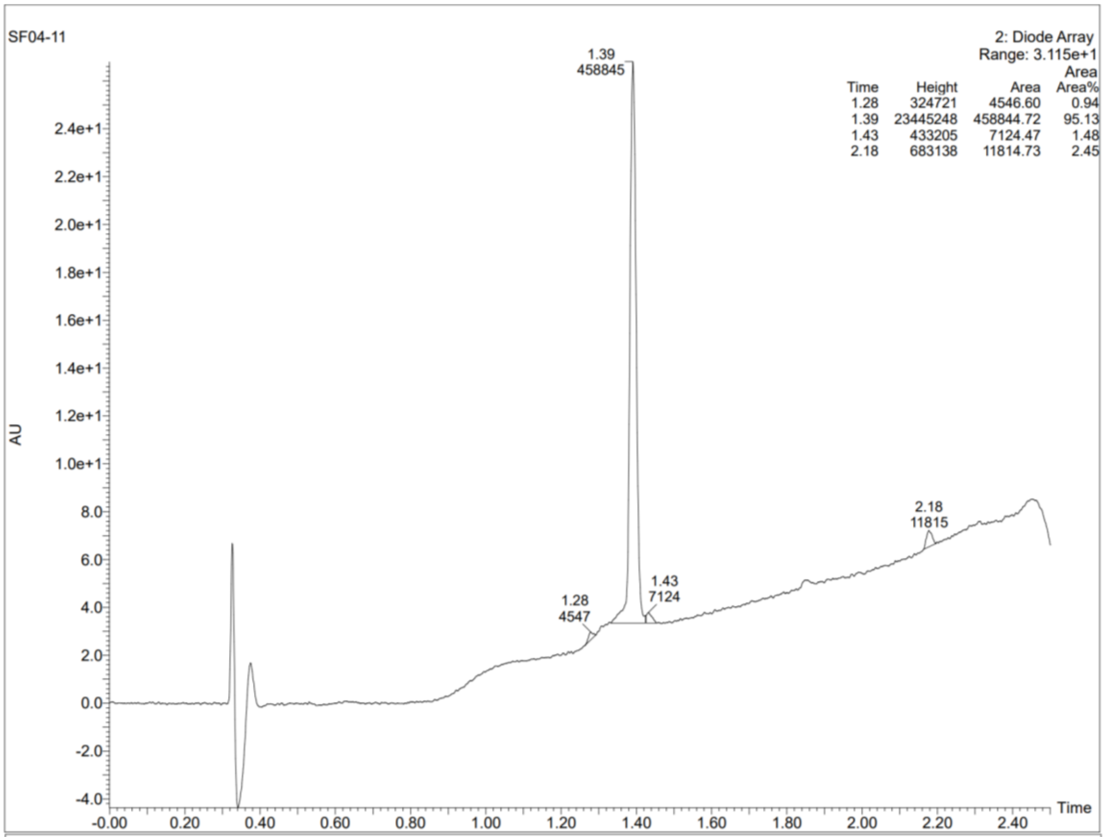

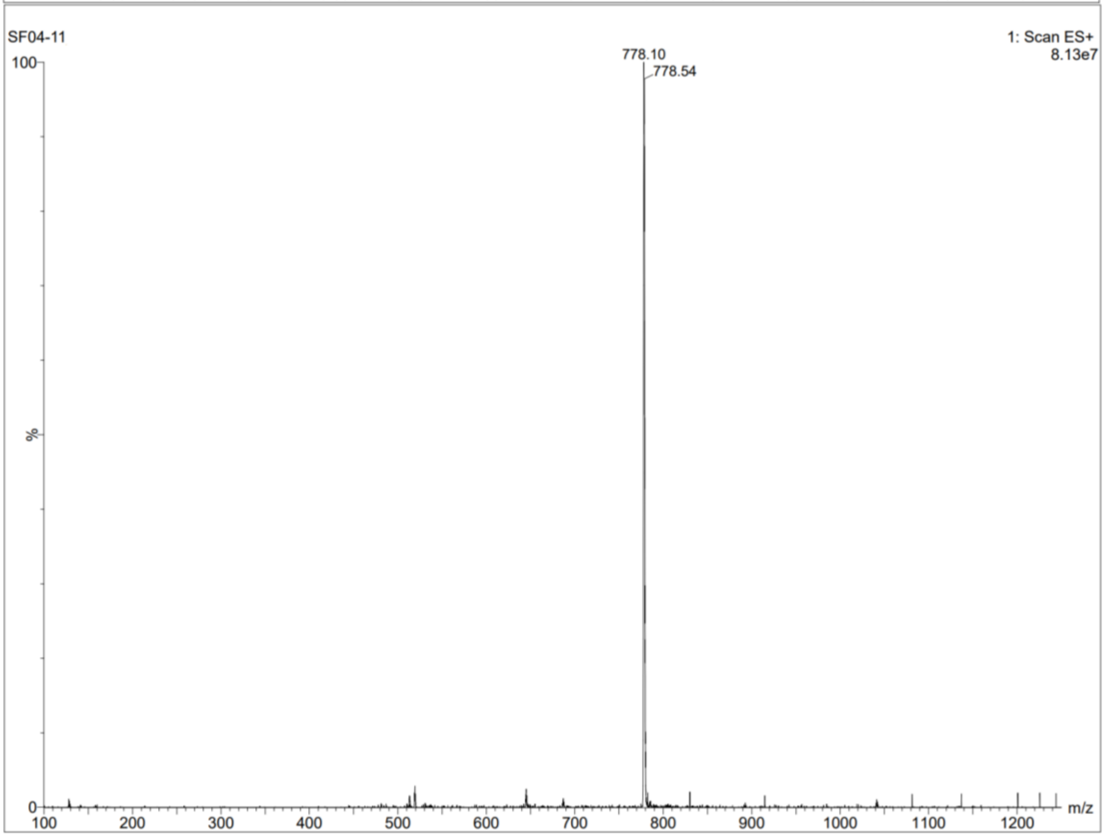

SF03-45: UPLC-MS

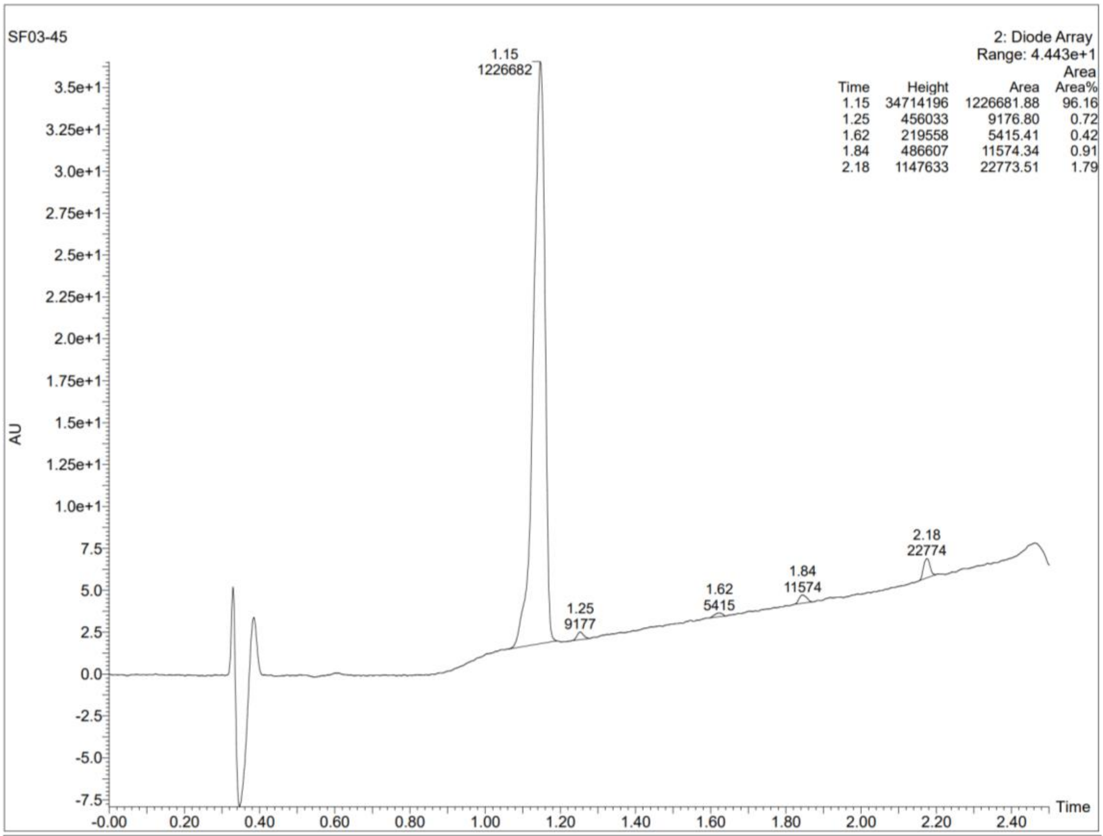

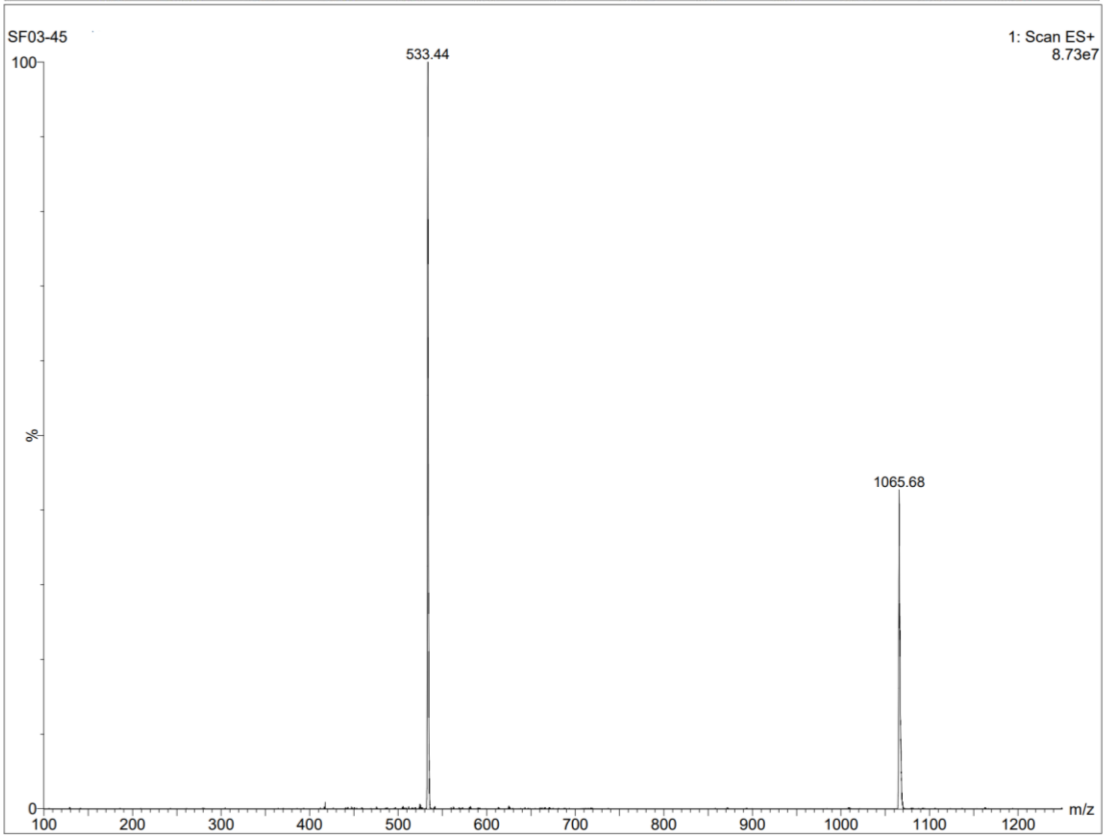

SF04-05: UPLC-MS

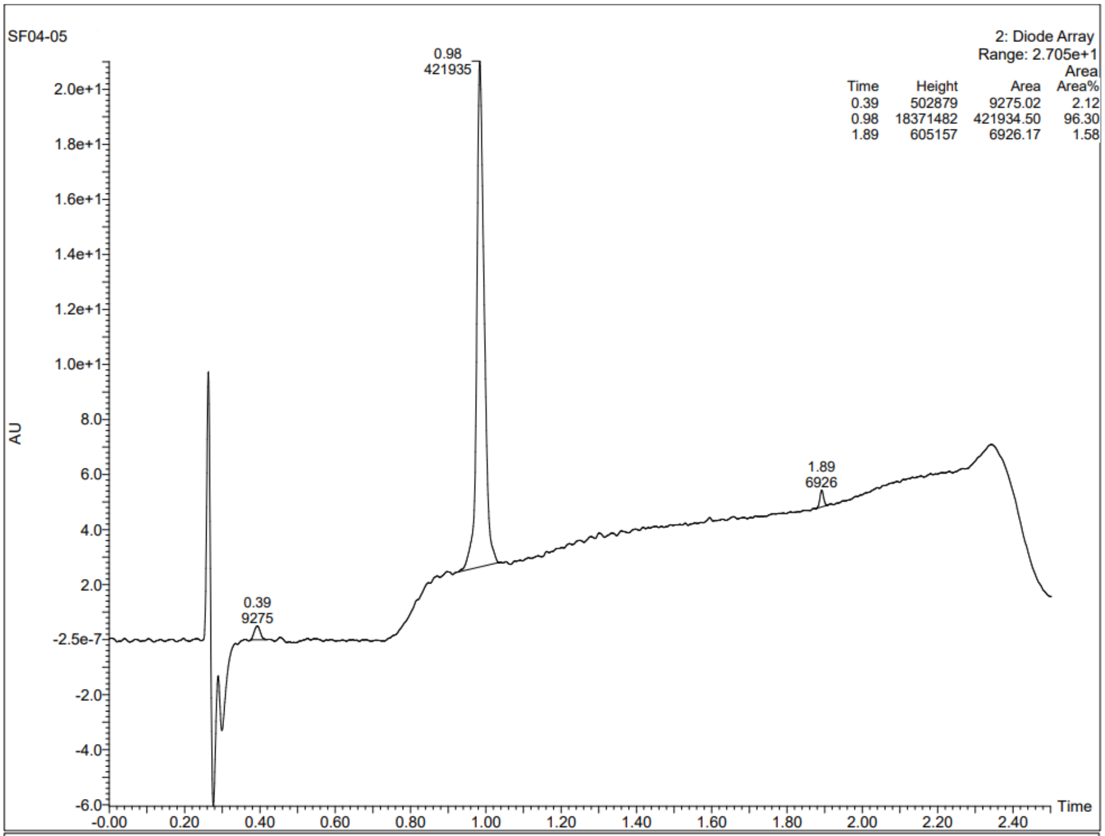

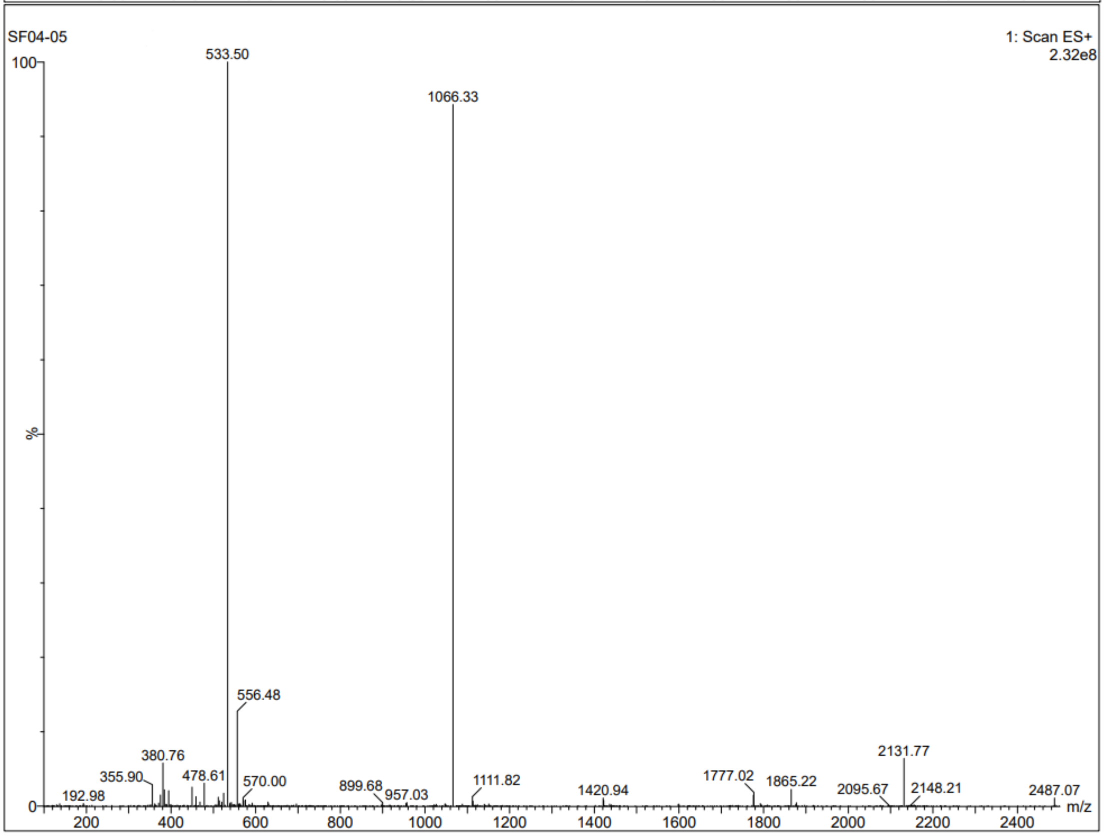

SF04-06: UPLC-MS

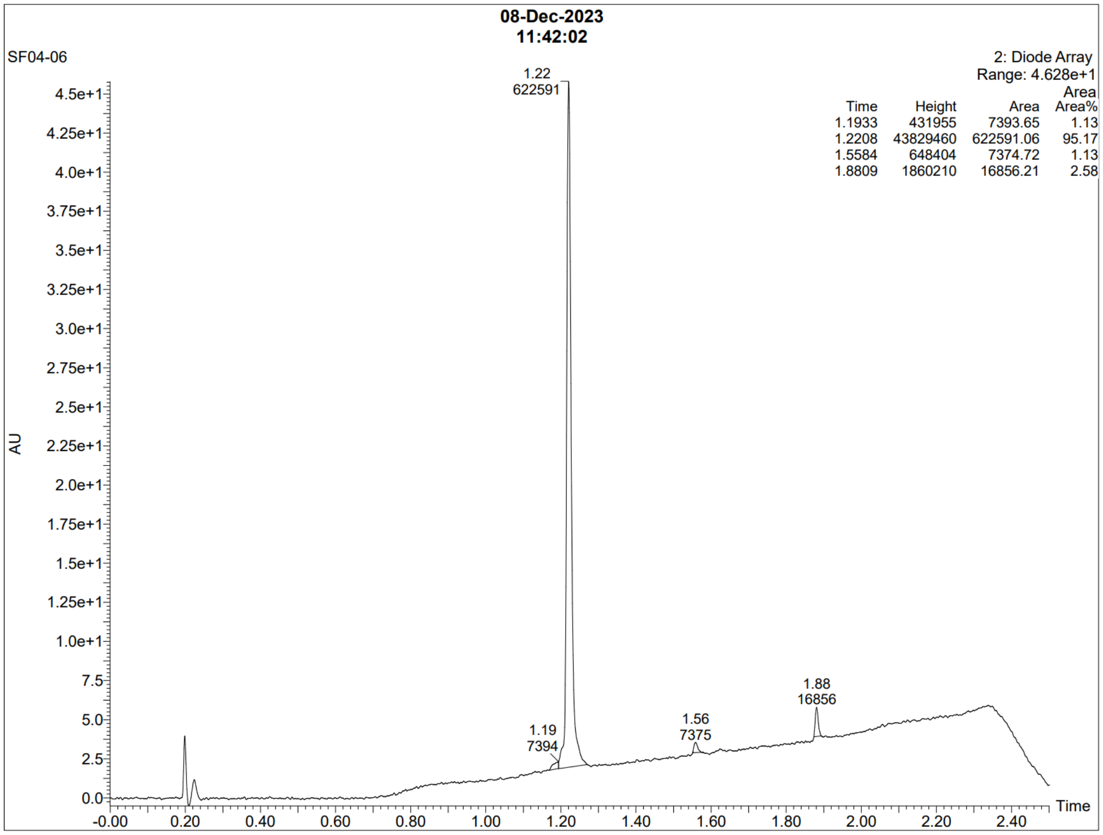

Time	Height	Area	Area Area%
1.1933	431955	7393.65	1.13
1.2208	43829460	622591.06	95.17
1.5584	648404	7374.72	1.13
1.8809	1860210	16856.21	2.58

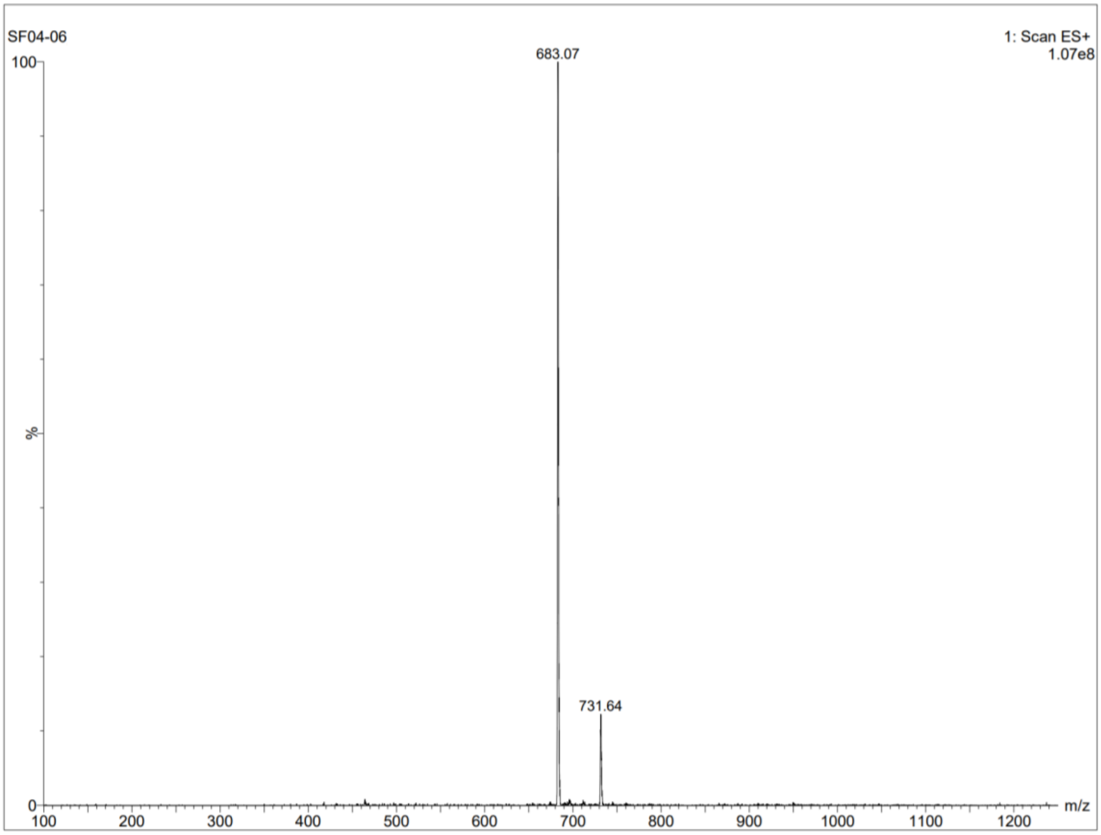

SF04-12: UPLC-MS

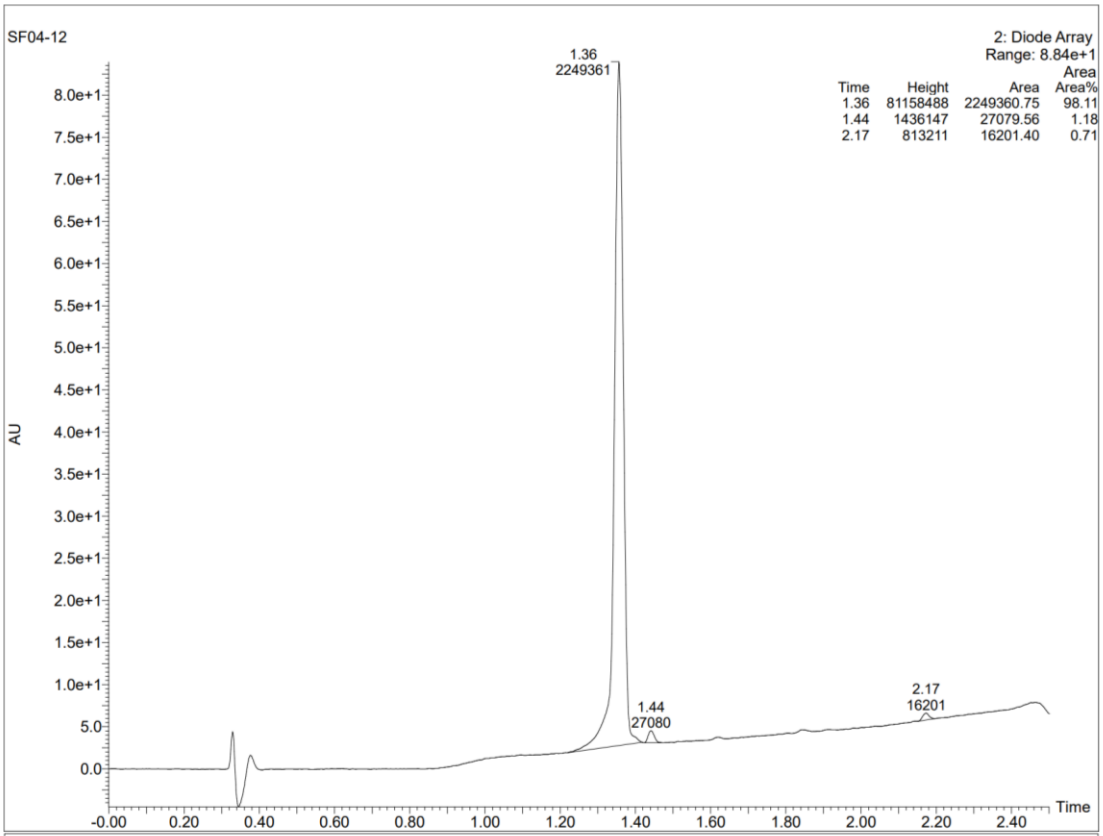

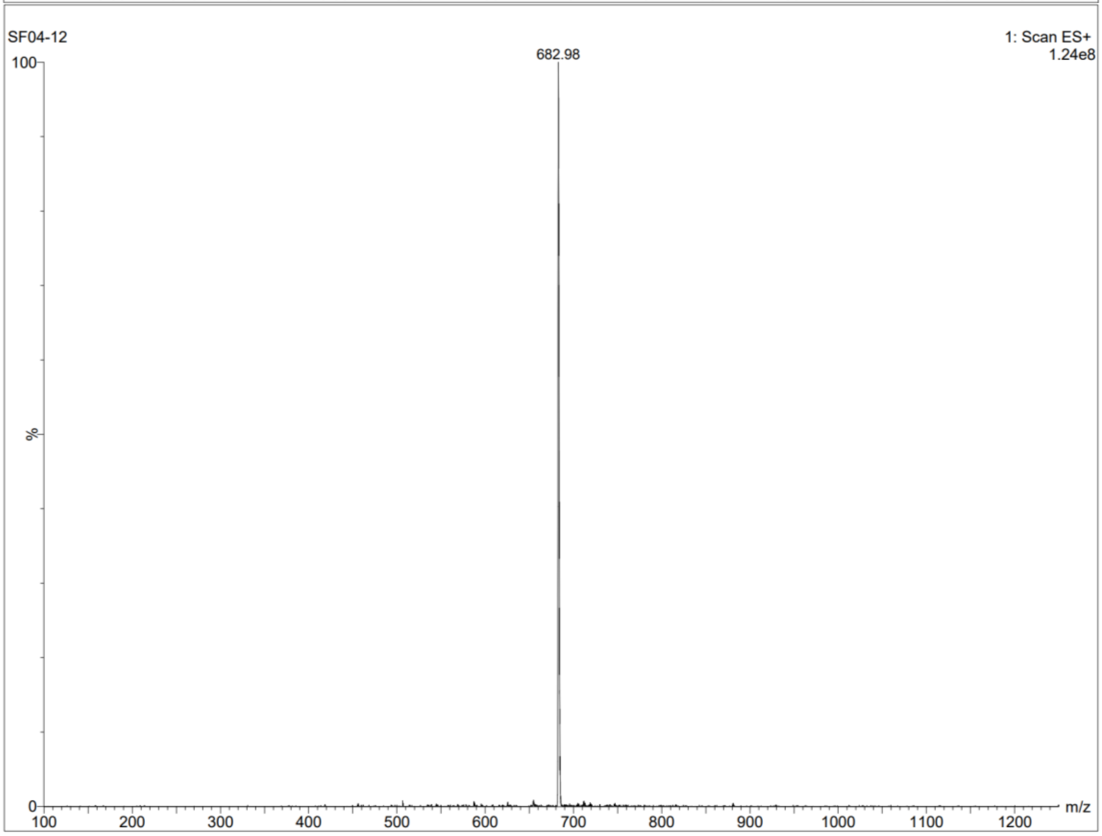

SF03-46: UPLC-MS

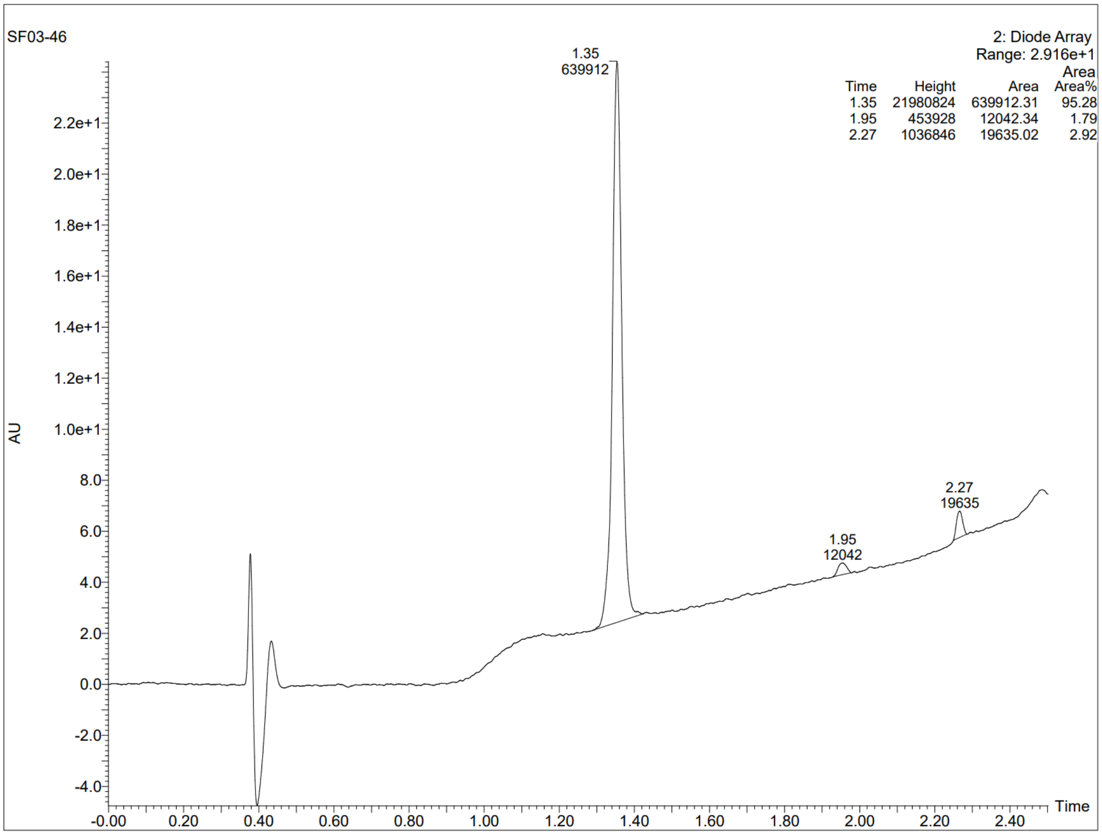

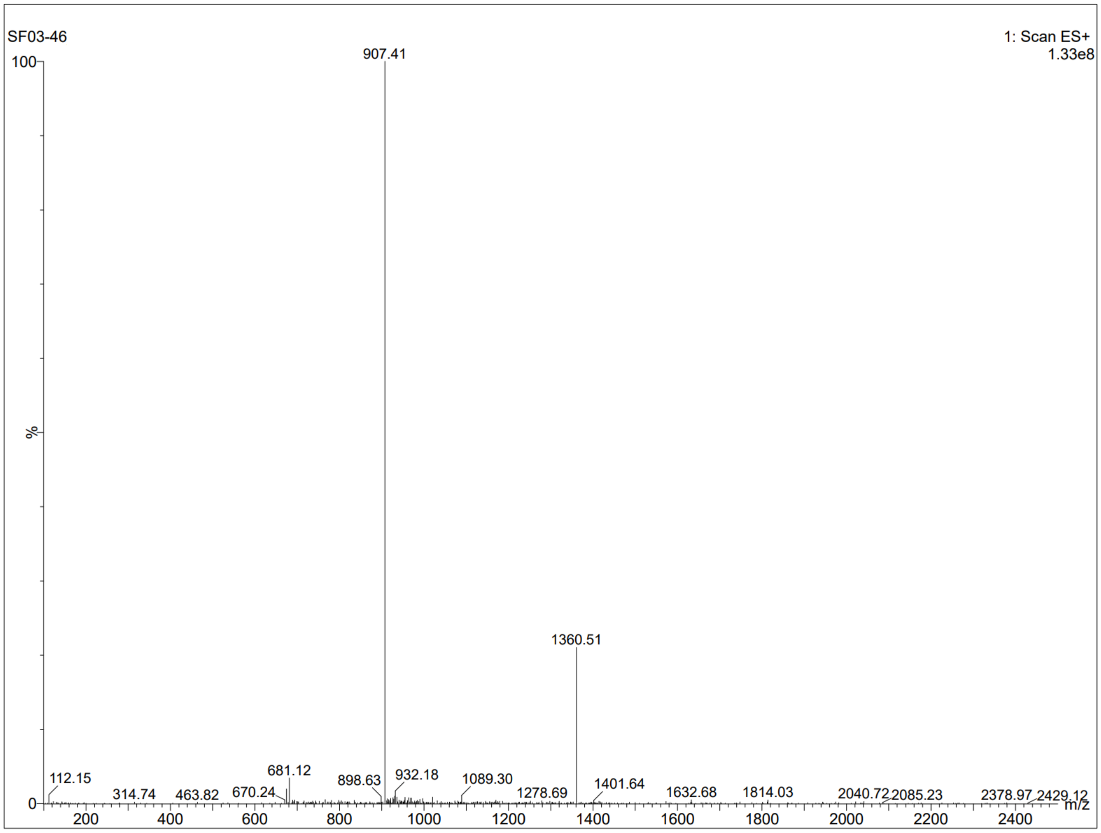

SF03-47: UPLC-MS

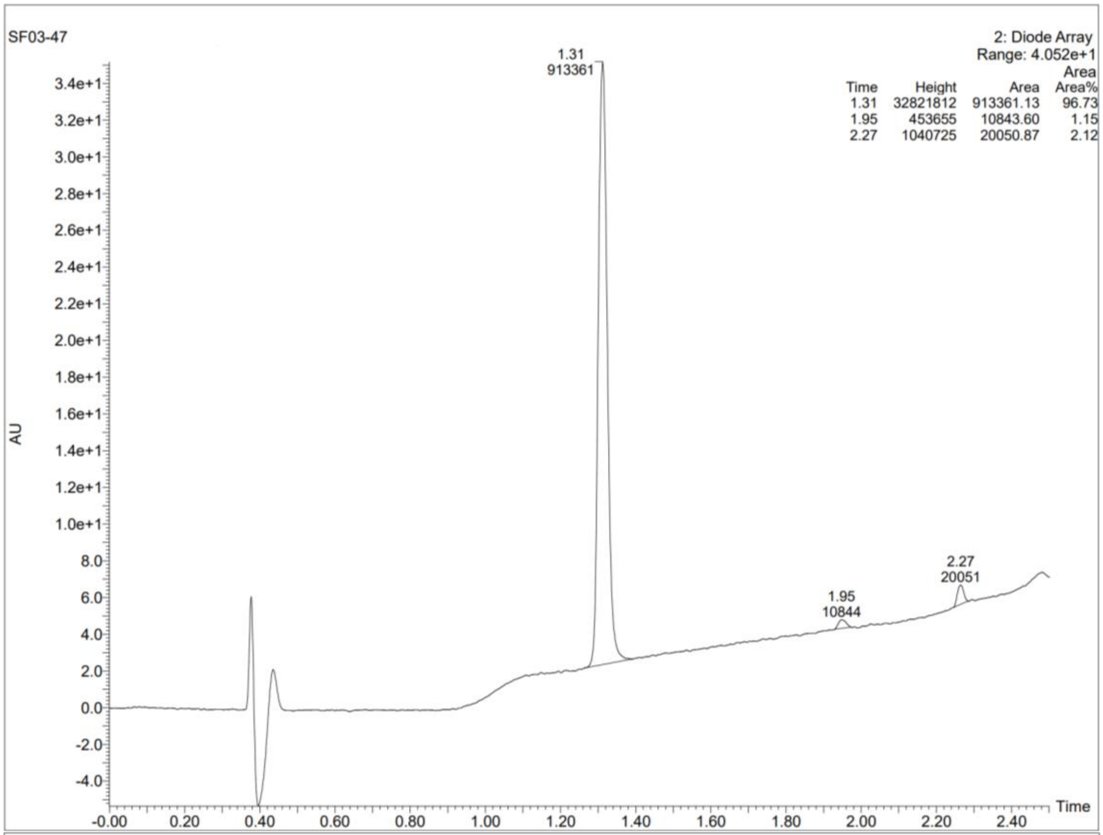

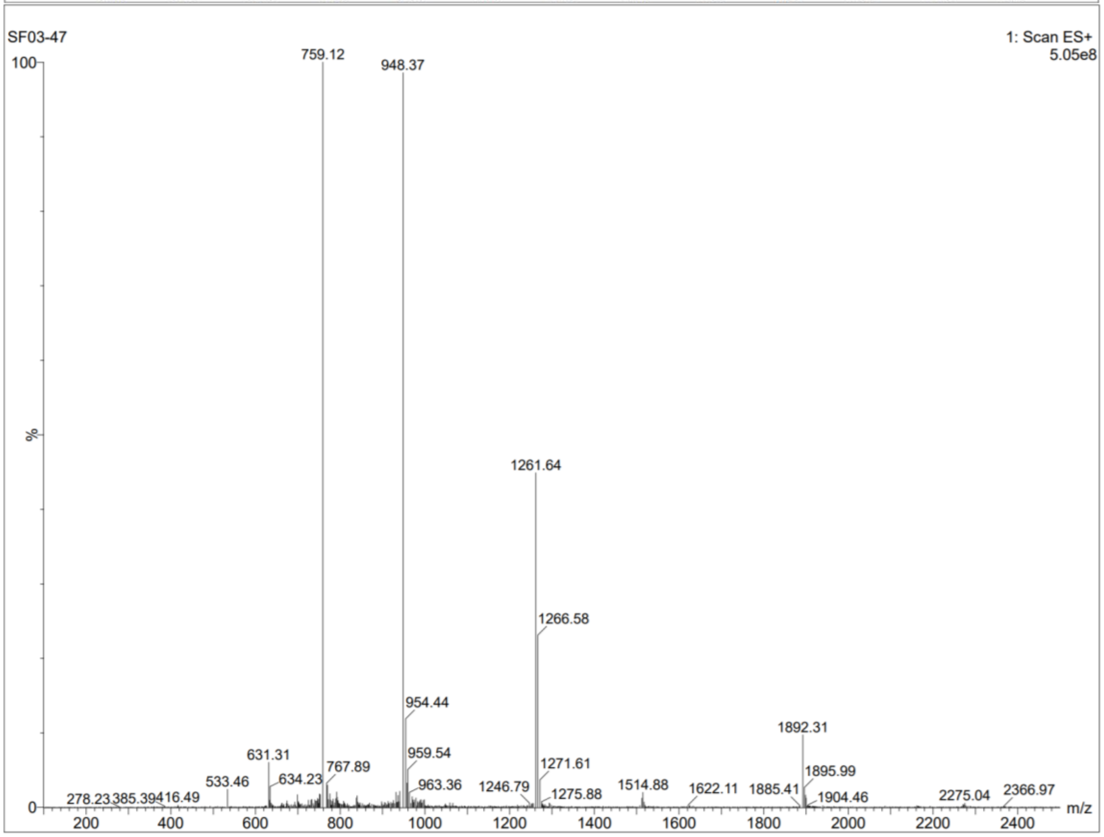

SF03-47 Scrambled: UPLC-MS

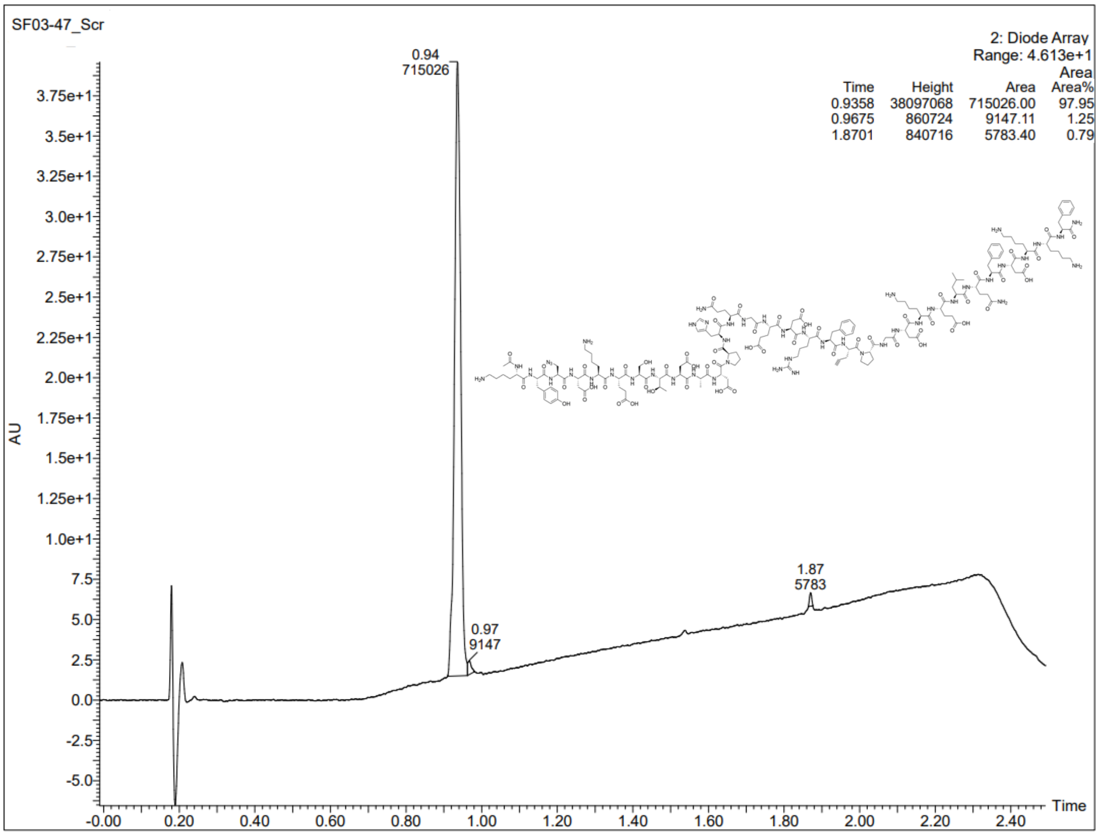

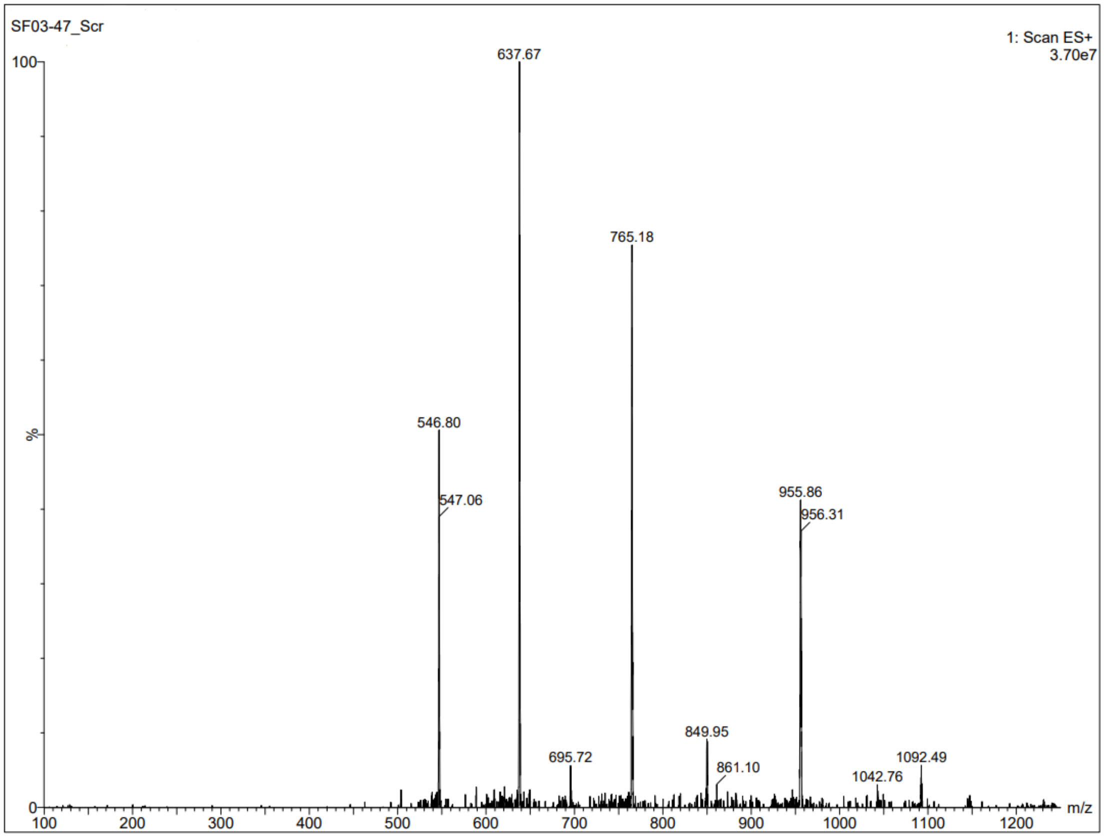

2.2 Supplementary NMR spectra

SF03-45: 1D ^{1}H NMR spectrum

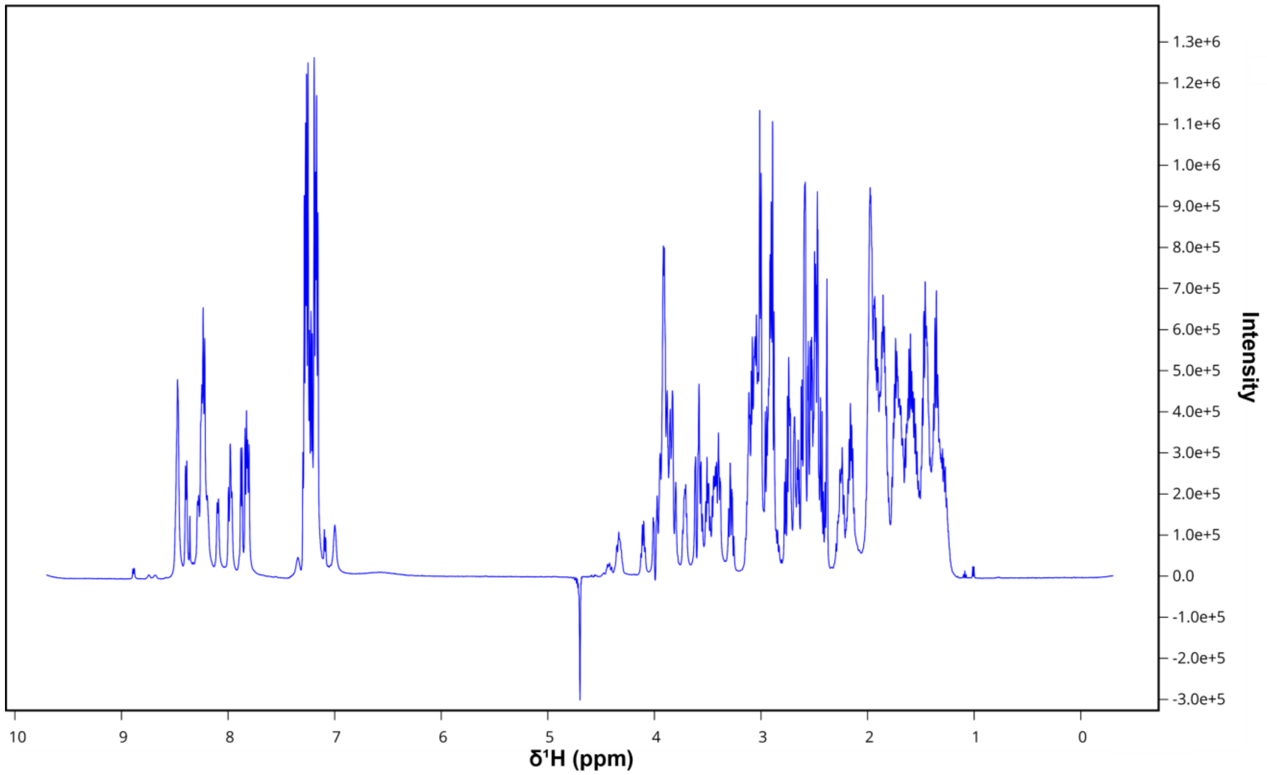

SF03-45: 2D TOCSY NMR spectrum

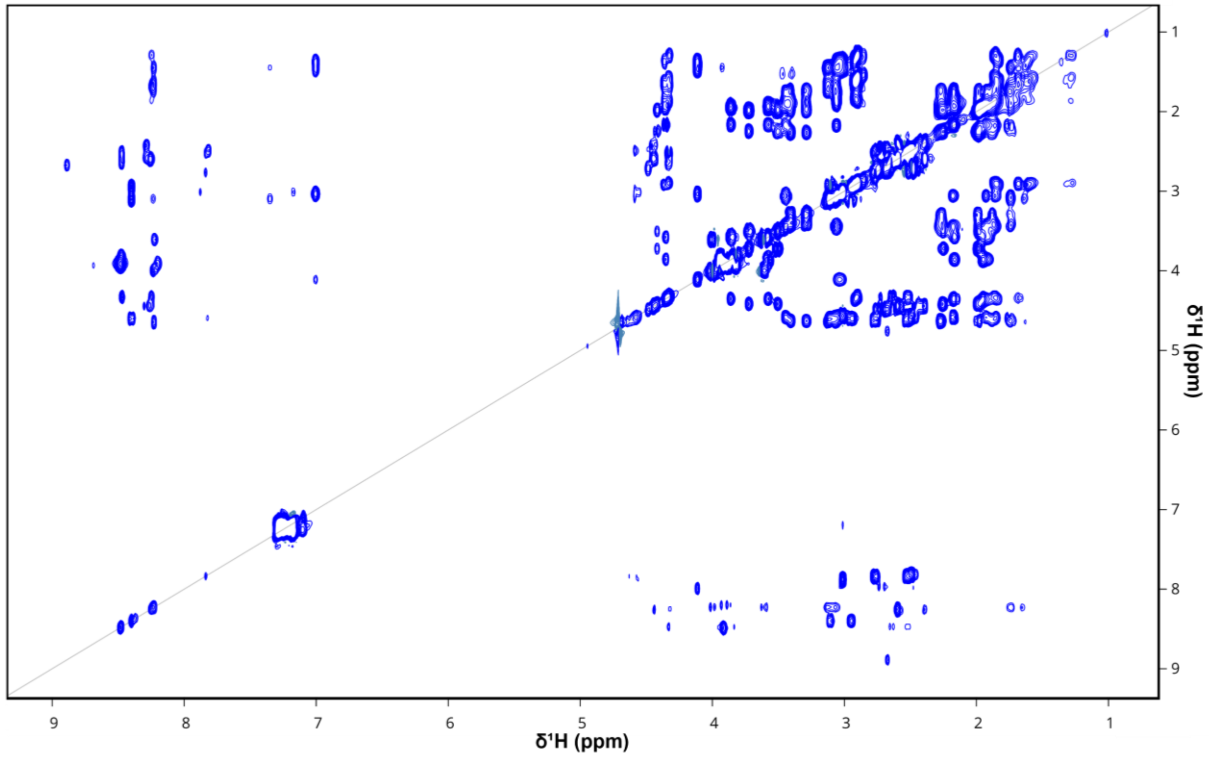

Supplement: Supplementary file 1 [file jm5c03450_si_001.pdf]
